# ADAMTS12 promotes fibrosis by restructuring extracellular matrix to enable activation of injury-responsive fibroblasts

**DOI:** 10.1172/JCI170246

**Published:** 2024-09-17

**Authors:** Konrad Hoeft, Lars Koch, Susanne Ziegler, Ling Zhang, Steffen Luetke, Maria C. Tanzer, Debashish Mohanta, David Schumacher, Felix Schreibing, Qingqing Long, Hyojin Kim, Barbara M. Klinkhammer, Carla Schikarski, Sidrah Maryam, Mathijs Baens, Juliane Hermann, Sarah Krieg, Fabian Peisker, Laura De Laporte, Gideon J.L. Schaefer, Sylvia Menzel, Joachim Jankowski, Benjamin D. Humphreys, Adam Wahida, Rebekka K. Schneider, Matthias Versele, Peter Boor, Matthias Mann, Gerhard Sengle, Sikander Hayat, Rafael Kramann

**Affiliations:** 1 Department of Medicine 2 (Nephrology, Rheumatology, Clinical Immunology and Hypertension), RWTH Aachen University, Medical Faculty, Aachen, Germany.; 2 Department of Pediatrics and Adolescent Medicine,; 3 Center for Biochemistry, Medical Faculty, and; 4 Center for Molecular Medicine Cologne, University of Cologne, Cologne, Germany.; 5 Cologne Center for Musculoskeletal Biomechanics (CCMB), Cologne, Germany.; 6 The Walter and Eliza Hall Institute of Medical Research, Melbourne, Victoria, Australia.; 7 Department of Medical Biology, University of Melbourne, Melbourne, Victoria, Australia.; 8 Max-Planck Institute of Biochemistry, Martinsried, Germany.; 9 Department of Anesthesiology, RWTH Aachen University, Aachen, Germany.; 10 Sequantrix GmbH, Aachen, Germany.; 11 Institute of Pathology, RWTH Aachen University, Aachen, Germany.; 12 CISTIM Leuven vzw, Leuven, Belgium.; 13 Institute for Molecular Cardiovascular Research, RWTH Aachen University, Medical Faculty, Aachen, Germany.; 14 Institute of Biochemistry and Molecular Biology, and; 15 Institute of Technical and Macromolecular Chemistry, RWTH Aachen University, Aachen, Germany.; 16 Institute of Applied Medical Engineering, Department of Advanced Materials for Medicine, University Hospital RWTH Aachen, Aachen, Germany.; 17 DWI-Leibniz Institute of Interactive Materials, Aachen, Germany.; 18 Division of Nephrology, Department of Medicine, Washington University in St. Louis, St. Louis, Missouri, USA.; 19 Institute of Metabolism and Cell Death, Helmholtz Zentrum München, Neuherberg, Germany.; 20 Department of Hematology, Erasmus MC Cancer Institute, Rotterdam, The Netherlands.; 21 Department of Cell Biology, Institute for Biomedical Technologies, RWTH Aachen University, Aachen, Germany.

**Keywords:** Cardiology, Nephrology, Fibrosis

## Abstract

Fibrosis represents the uncontrolled replacement of parenchymal tissue with extracellular matrix (ECM) produced by myofibroblasts. While genetic fate-tracing and single-cell RNA-Seq technologies have helped elucidate fibroblast heterogeneity and ontogeny beyond fibroblast to myofibroblast differentiation, newly identified fibroblast populations remain ill defined, with respect to both the molecular cues driving their differentiation and their subsequent role in fibrosis. Using an unbiased approach, we identified the metalloprotease *ADAMTS12* as a fibroblast-specific gene that is strongly upregulated during active fibrogenesis in humans and mice. Functional in vivo KO studies in mice confirmed that *Adamts12* was critical during fibrogenesis in both heart and kidney. Mechanistically, using a combination of spatial transcriptomics and expression of catalytically active or inactive ADAMTS12, we demonstrated that the active protease of ADAMTS12 shaped ECM composition and cleaved hemicentin 1 (HMCN1) to enable the activation and migration of a distinct injury-responsive fibroblast subset defined by aberrant high JAK/STAT signaling.

## Abstract 2

Catalytically active ADAMTS12 controls fibrosis by restructuring ECM to enable activation and migration of a distinct injury-responsive fibroblast subset after injury.

## Graphical abstract

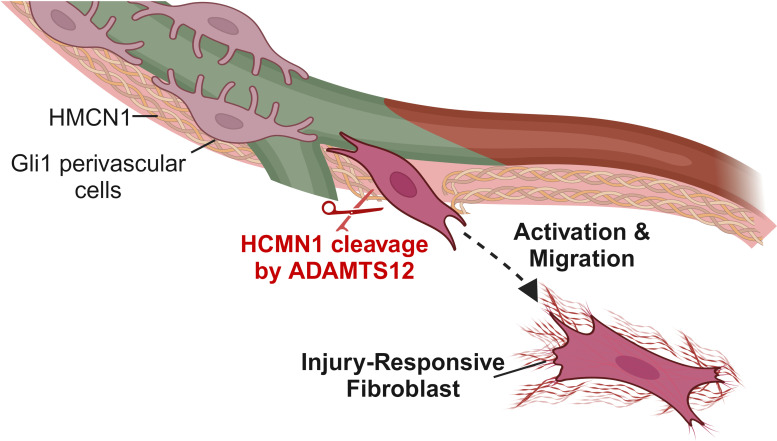

## Introduction

Fibrosis represents the uncontrolled replacement of parenchymal tissue by extracellular matrix (ECM) (1). Whereas deposition of ECM sustains tissue integrity immediately after injury, continued deposition of ECM by myofibroblasts drives maladaptive tissue remodeling and organ failure. Representing a pressing and unmet clinical need, fibrosis constitutes the common final path of injury across virtually all organs and is considered to be accountable for up to 45% of deaths in the industrialized world (1).

Myofibroblasts, which expand after injury, are considered to be the major source of ECM during fibrogenesis. While lineage fate tracing and single-cell RNA-Seq (scRNA-seq) technologies have helped elucidate (myo)fibroblast ontogeny, they also uncovered a hitherto unexpected degree of fibroblast heterogeneity extending beyond the traditional fibroblast-to-myofibroblast differentiation paradigm (1–4). However, these newly identified fibroblast subsets remain ill defined, both with respect to the molecular cues driving their differentiation and their exact role during fibrogenesis.

We recently showed that a small perivascular cell population, marked by the transcription factor Gli1, is a major contributor to the myofibroblast pool across organs (5, 6). After injury, Gli1^+^ cells expand, migrating from the perivascular niche into the interstitium and differentiating into myofibroblasts. Interestingly, we observed that beyond Gli1^+^ myofibroblast differentiation, Gli1^+^ cell detachment and migration from the perivascular niche was an independent contributor to capillary destabilization perpetuating injury (7).

Here, using an unbiased approach to assess Gli1^+^ cell activation, we identified the metalloprotease *ADAMTS12* as a fibroblast-specific gene that was strongly upregulated during active fibrotic remodeling in mice and humans. Importantly, in vivo experiments confirmed that *Adamts12* controlled fibrogenesis and fibroblast expansion across organs. Mechanistically, by leveraging spatial transcriptomics, CRISPR/Cas9 gene editing and expression of catalytically active/inactive ADAMTS12, we reveal that catalytically active ADAMTS12 drove fibroblast activation toward an injury-responsive fibroblast cell state that was defined by higher cell migration via cleavage of the fibulin hemicentin 1 (HMCN1).

## Results

**Figure 1:**
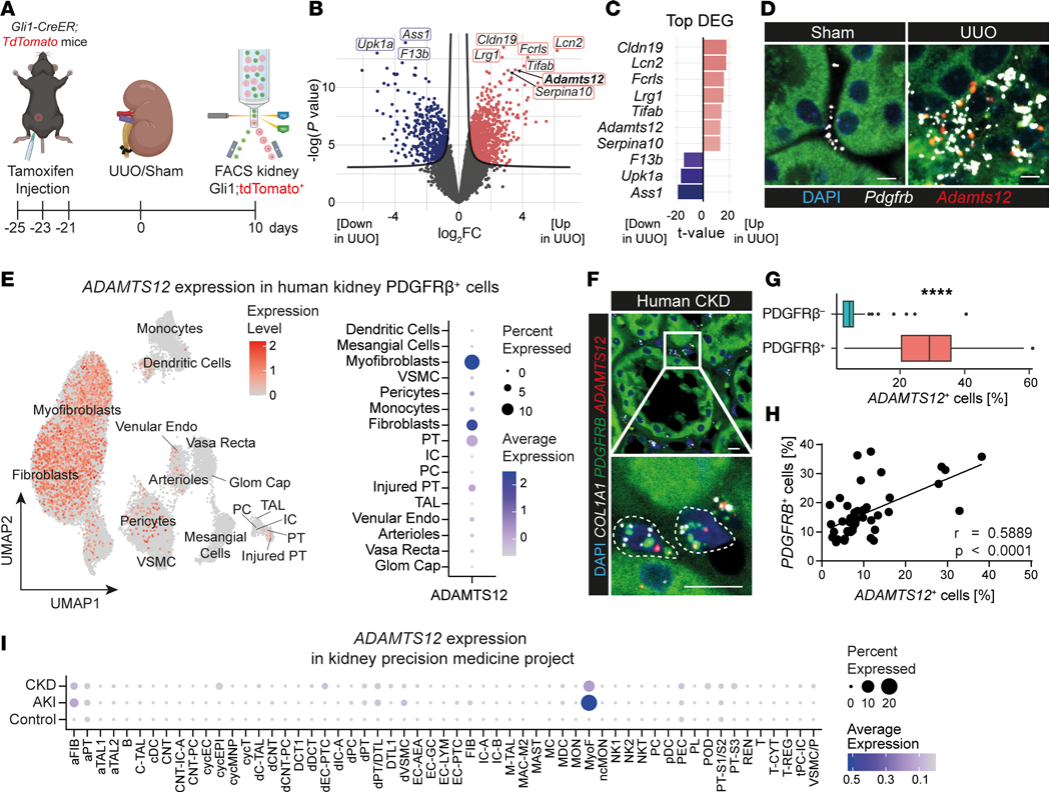
*Adamts12* is specifically upregulated in fibroblasts after injury.(A) Experimental design. The schematic drawing was created with BioRender (BioRender.com). (**B**) Volcano plot of DEGs in kidney Gli1^+^ cells after UUO versus sham surgery (*n* = 3 per group). (**C**) Top up- and downregulated genes, ordered by *t* value. (**D**) ISH for *Pdgfrb* and *Adamts12* in murine kidneys 10 days after UUO or sham surgery. Scale bars: 10 μm. (**E**) Feature and dot plot of *ADAMTS12* expression in a published scRNA-Seq dataset of human CKD (2). Labels refer to cell types (Supplemental Table 3). (**F**) Representative image of ISH stainings of *ADAMTS12*, *COL1A1*, and *PDGFRB* in human kidneys. Scale bars: 10 μm. (**G**) Quantification of the percentage of *ADAMTS12*^+^ cells in *PDGFRB*^+^ or *PDGFRB*^–^ cells (*n* = 43). ****P < 0.0001, by 2-tailed, paired *t* test. (**H**) Pearson’s correlation of the percentage of *ADAMTS12*^+^ cells with the percentage of *PDGFRB*^+^ cells in human nephrectomies. (**I**) Dot plot of *ADAMTS12* gene expression in a scRNA-Seq dataset published by the Kidney Precision Medicine Project (ref. 11). Labels refer to cell types (Supplemental Table 5). Up, upregulated; Down, downregulated.

To dissect the molecular cues that drive fibroblast activation and differentiation after injury, we isolated renal tdTomato^+^Gli1^+^ cells from tamoxifen-induced Gli1CreER^t2^ tdTomato mice after sham or unilateral ureter obstruction (UUO) surgery using FACS (Figure 1A). To investigate Gli1^+^ cell activation in an unbiased fashion, we subsequently measured transcriptome gene expression. After quality control and data normalization, principal component analysis (PCA) revealed clear separation of Gli1^+^ cells after either sham or UUO surgery along the first principal component (PC1) (Supplemental Figure 1, A and B; supplemental material available online with this article; https://doi.org/10.1172/JCI170246DS1). Differential expression analysis (UUO vs. sham) identified strong upregulation of injury-associated genes (*Lcn2*, *Fcrls*) in UUO Gli1^+^ cells (Figure 1B and Supplemental Table 1). In line with these findings, both PROGENy pathway and DoRothEA transcription factor analysis predicted increased activity of fibrosis-associated pathways (JAK/STAT, TGF-β) (Supplemental Figure 1C) and transcription factors such as Spi1 (8) (Supplemental Figure 1D) in UUO Gli1^+^ cells. Intriguingly, the metalloprotease ADAM metallopeptidase with thrombospondin Type 1 motif 12 (*Adamts12)* ranked among the top upregulated genes in Gli1^+^ cells after UUO (Figure 1C). While others have previously described a role for metalloproteases in fibrosis, the function of ADAMTS12 in fibrosis remains unclear (9).

To verify the upregulation of *Adamts12* expression in fibroblasts after renal injury, we performed *Adamts12* ISH after UUO surgery, kidney ischemia reperfusion injury (IRI), and adenine-induced kidney injury (Figure 1D and Supplemental Figure 1, E–J). Automated quantification confirmed an interstitial expression pattern for *Adamts12* with minimal expression in homeostasis as well as early after kidney injury (day 1), but strong upregulation between day 5 and day 10 after UUO surgery, day 14 after IRI, and a slight upregulation at day 7 in adenine-induced kidney injury, all of which are time points that coincide with fibroblast expansion, migration, and fibrogenesis (Supplemental Figure 1, E–J). To confirm mesenchyme-restricted *Adamts12* expression, we next analyzed a publicly available single-nucleus RNA-Sequencing (snRNA-Seq) time course of murine IRI and detected fibroblast- and pericyte-specific *Adamts12* expression, with the strongest upregulation by fibroblasts on day 2 after acute kidney injury (AKI) (Supplemental Figure 1, K and L) (10).

To translate our findings to human disease, we next analyzed *ADAMTS12* expression in recently published human kidney scRNA-Seq data on PDGFRβ^+^ cells (mesenchyme enriched, *n* = 8), CD10^–^ cells (proximal tubule depleted, *n* = 15), and a large kidney dataset of AKI and chronic kidney disease (CKD) (Kidney Precision Medicine Project KPMP], *n* = 47) (2, 11). *ADAMTS12* expression at the highest granularity of annotation in the PDGFRβ^+^ and CD10^–^ datasets confirmed an expression pattern restricted to mesenchymal cells, with the strongest expression detected in the myofibroblast subset 1, which was defined by high *COL1A1* and *POSTN* expression (Figure 1E and Supplemental Figure 2, A–F). Further stratification of *ADAMTS12* expression by disease in the KPMP dataset revealed a distinct upregulation of *ADAMTS12* expression in myofibroblasts and adaptive fibroblasts during AKI and, to a lesser extent, CKD (Figure 1I and Supplemental Figure 2F).

To validate our findings, we next performed multiplex ISH for *COL1A1*, *PDGFRB*, and *ADAMTS12* in human kidney tissue (*n* = 43), which demonstrated that *ADAMTS12* expression was indeed specific to *PDGFRB*^+^ and *COL1A1*^+^ cells (Figure 1, F and G, and Supplemental Figure 2G). More important, ISH confirmed that the number of *ADAMTS12*^+^ cells correlated with both the number of *PDGFRB*^+^ cells as well as the overall severity of fibrosis as quantified by Picrosirius red stainings (Figure 1H and Supplemental Figure 2, H and I). In summary, we demonstrate a strong upregulation of *ADAMTS12* in mesenchymal cells after injury in both murine and human kidneys, suggesting a potentially conserved role for ADAMTS12 in fibrosis.

Next, we aimed to identify the upstream signaling pathways controlling *ADAMTS12* expression in fibroblasts. Here, we subsetted fibroblasts from a publicly available multiomics SNARE-Seq kidney dataset (11) for gene regulatory network analysis (Supplemental Figure 2, J and K). ArchR trajectory inference confirmed the upregulation of myofibroblast markers *COL1A1*, *POSTN*, and *DCN*, as well as *ADAMTS12* along pseudotime of the myofibroblast-specific embedding (Supplemental Figure 2L). Subsequent gene regulatory network analysis based on fibroblast trajectories identified 2 transcription factors that significantly correlated with *ADAMTS12* expression: *BACH1* and *JUNB* (Supplemental Figure 2M and Supplemental Table 6). Both transcription factors have been previously identified as drivers of fibrosis, indicating that *ADAMTS12* might act as a downstream effector gene of the latter (12–14).

**Figure 2:**
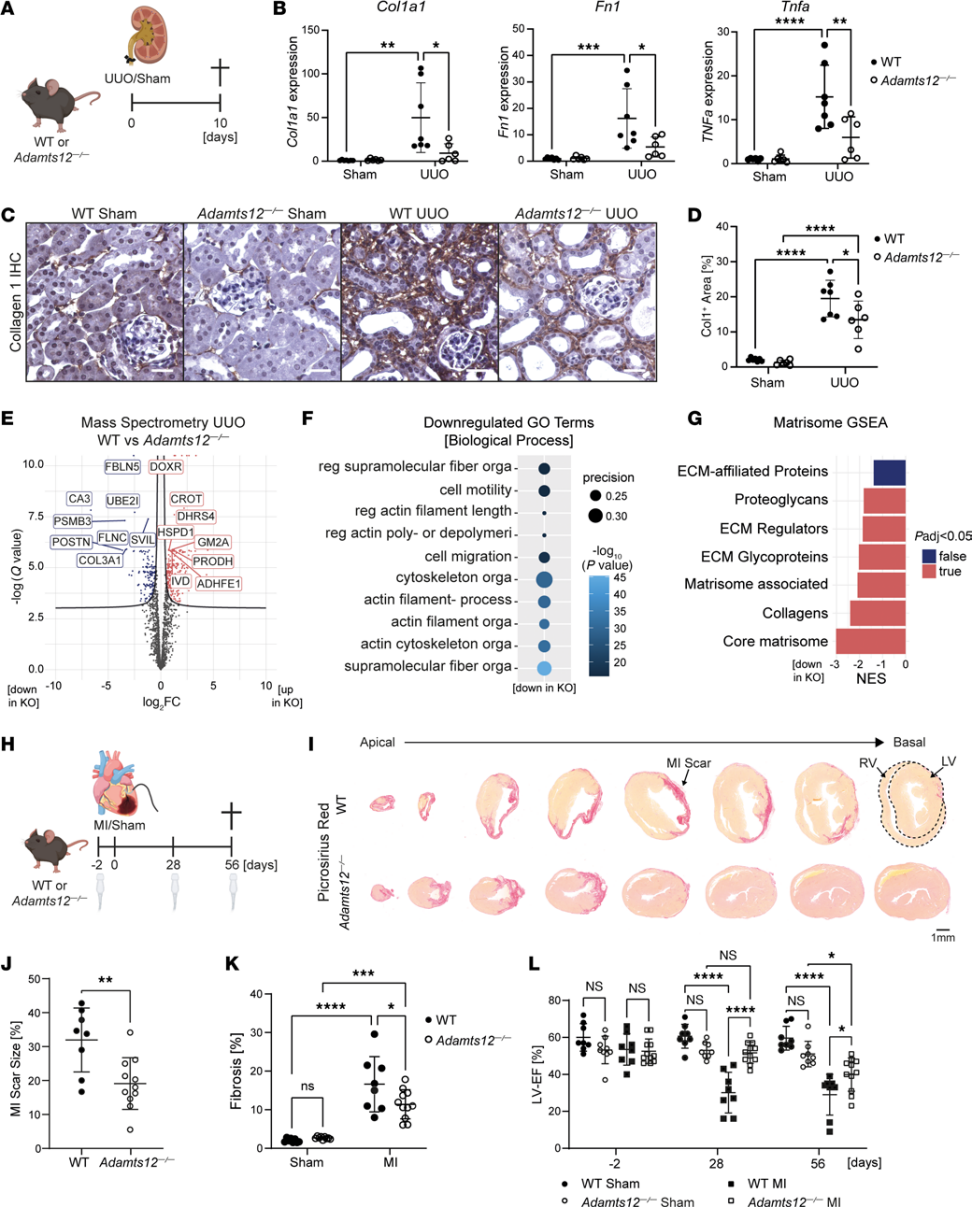
Genetic loss of *Adamts12* ameliorates fibrosis in kidney and heart. (**A**) Experimental design for UUO surgery (*n* = 7 WT, *n* = 6 *Adamts12^–/–^*). (**B**) RT-qPCR for *Col1a1* (*P*_WT_ _UUO_ _vs._ _Adamts12–/– UUO_ = 0.0127), *Fn1* (*P*_WT_ _UUO_ _vs._ _Adamts12–/–_ _UUO_ = 0.0228), and *Tnfa* (*P*_WT_ _UUO_ _vs. *Adamts12*–/–_ _UUO_=0.0068) in kidneys from WT or *Adamts12^–/–^* mice after UUO surgery. (**C**) Representative images of collagen 1 IHC. Scale bars: 25 μm. (**D**) Quantification of Col1^+^ area (in percentage) based on the immunohistochemical stainings shown in **C** (*P*_WT UUO_ _vs._ _Adamts12–/–_ _UUO_ = 0.0398). (**E**) Volcano plot of differentially expressed proteins in UUO kidneys from WT versus *Adamts12^–/–^* mice. log_2_FC, log_2_ fold change. (**F**) Top enriched Biological Process GO terms based on downregulated proteins in *Adamts12^–/–^* mice. reg, regulation of; orga,organization; poly- or depolymeri, polymerization or depolymerization; filament-, filament-based. (**G**) Matrisome GSEA based on MS data on UUO kidneys from WT versus *Adamts12^–/–^* mice. *P*adj, adjusted *P* value; NES, normalized enrichment score. (**H**) Experimental design for MI surgery (WT = 8 for each condition, *Adamts12*^–/–^ sham *n* = 9, *Adamts12*^–/–^ MI *n* = 11). (**I**) Representative images of Picrosirius red staining in WT and *Adamts12*^–/–^ mice after MI. Scale bar: 1 μm. RV, right ventricle; LV, left ventricle. (**J**) Quantification of MI scar size (*P*_WT_ _MI_ _vs._ _Adamts12—/—_ _MI_ = 0.0044, unpaired *t* test). (**K**) Quantification of fibrosis determined by spectral thresholding analysis of red ECM (*P*_WT_ _MI_ _vs._ _Adamts12—/—_ _MI_ = 0.0385). (**L**) LV-EF measured by Simpson’s method at days –2, 28 (*P*_WT_ _MI_ _vs._ _Adamts12—/—_ _MI_ < 0.0001), and 56 (*P*_WT_ _MI_ _vs._ _Adamts12—/—_ _MI_ < 0.0166). *P < 0.05, **P < 0.01, ***P < 0.001, and ****P < 0.0001. Unless otherwise specified, all comparisons were performed by 2-way ANOVA with Tukey’s post hoc test. The schematic drawings in **A** and **H** were created with BioRender (BioRender.com).

So far our data pointed toward a strong upregulation of *Adamts12* during fibrosis but left a void regarding its functional relevance. Thus, we tested the hypothesis that ADAMTS12 has a functional effect on fibrosis by performing UUO or sham surgery on WT and *Adamts12^–/–^* mice (Figure 2A and Supplemental Figure 3A). Ten days after UUO, reverse transcription quantitative PCR (RT-qPCR) revealed a markedly reduced expression of fibrosis- and inflammation-related genes such as *Col1a1*, *Fn1*, *Tgfb*, and *Tnfa* as well as a trend for reduced *Acta2* and *Il6* expression in *Adamts12^–/–^* mice (Figure 2B and Supplemental Figure 3A). In accordance, loss of *Adamts12* significantly reduced fibrosis as determined by quantification of collagen 1 IHC stainings and α smooth muscle actin (αSMA) Western blots (Figure 2, C and D, and Supplemental Figure 3, B and C). Further PDGFRβ immunofluorescence staining confirmed that loss of *Adamts12* not only mitigated collagen 1 deposition, but also the expansion of PDGFRβ^+^ mesenchymal cells after injury (Supplemental Figure 3, D and E). As prior studies reported a relevant role for ADAMTS12 in inflammation and angiogenesis (15, 16), we additionally assessed angiogenesis via CD31 and myeloid cell infiltration via CD11b immunofluorescence staining. Here, both stainings showed no significant differences between *Adamts12^–/–^* and WT mice after UUO surgery, indicating that *Adamts12* did not primarily affect myeloid cell infiltration or angiogenesis in the context of fibrosis (Supplemental Figure 3, F–I).

Importantly, fibrosis was insufficiently captured by analysis of singular proteins. For a comprehensive assessment of ECM remodeling, we therefore performed mass spectrometry (MS) of UUO kidneys from WT and *Adamts12^–/–^* mice. Differential analysis confirmed loss of fibrosis-defining proteins, such as COL3A1 and POSTN, in *Adamts12^–/–^* mice (Figure 2E and Supplemental Table 7). Next, Gene Ontology Biological Process (GO-BP) enrichment analysis based on significantly downregulated proteins identified cell motility, migration, and supramolecular fiber organization as enriched GO-BP terms (Figure 2F). Gene set enrichment analysis (GSEA) based on consensus matrisome gene sets (13), which stratify ECM proteins, revealed a loss of all ECM proteins, with the strongest loss of collagens in *Adamts12^–/–^* mice (Figure 2G). These findings not only confirm decreased fibrosis, but also highlight an altered ECM remodeling with decreased deposition of fibrosis-defining collagens following loss of *Adamts12*.

To assess whether the profibrotic effect of *Adamts12* is conserved across organs, we next performed myocardial infarction (MI) surgery or sham surgery in WT and *Adamts12^–/–^* mice (Figure 2H). Picrosirius red staining of serial heart sections confirmed that *Adamts12*-KO ameliorated both fibrosis and the overall scar size after MI (Figure 2, I–K). More important, *Adamts12^–/–^* mice exhibited a preserved left ventricular ejection fraction (LV-EF) at days 28 and 56 after MI (Figure 2L and Supplemental Video 1). In summary, we demonstrate that loss of *Adamts12* ameliorated fibrosis across organs and preserved cardiac function after injury.

**Figure 3:**
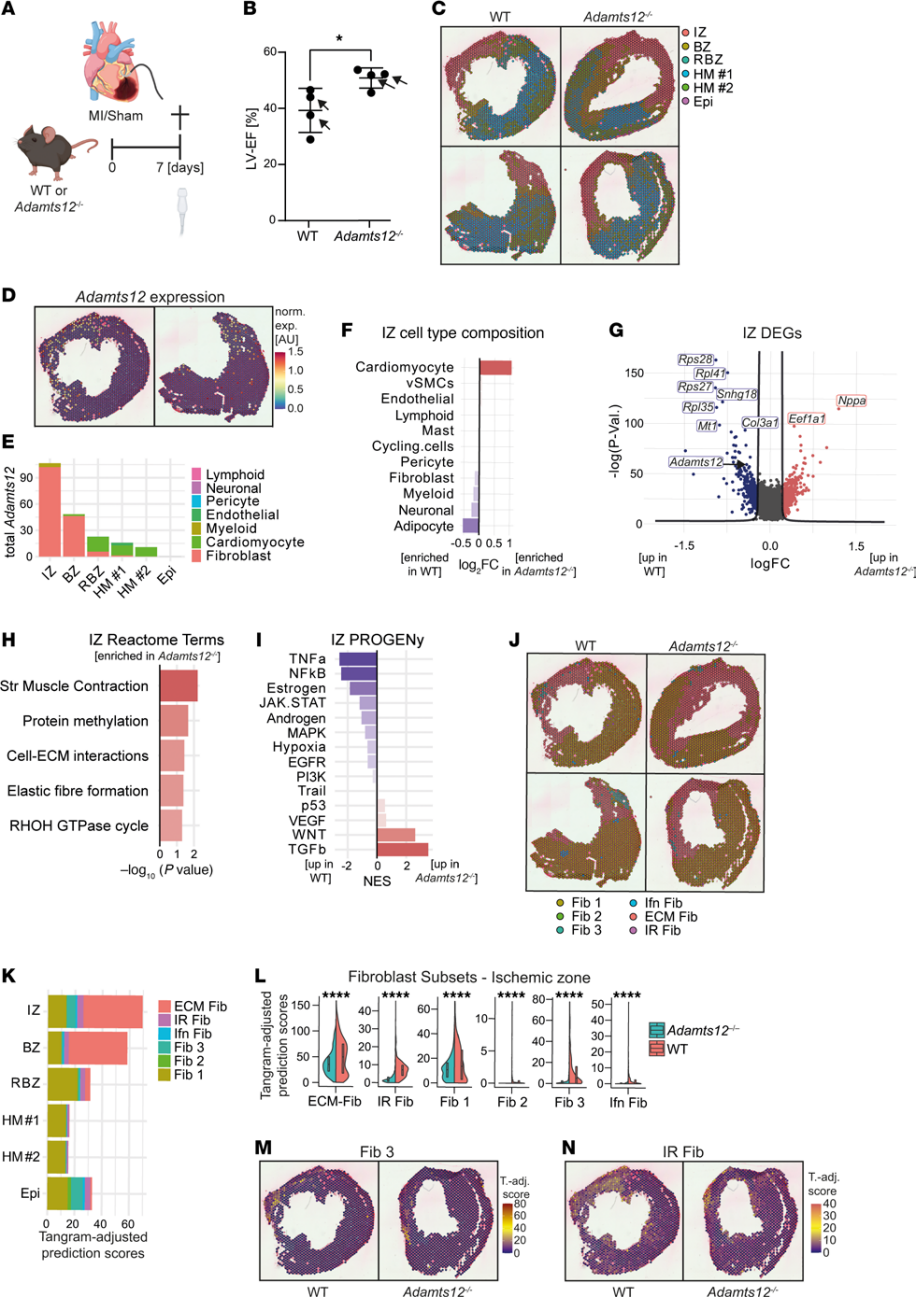
Visium spatial transcriptomics of WT and *Adamts12^–/–^* mice after MI. (**A**) Experimental design of MI surgeries for Visium spatial transcriptomics. The schematic drawing was created with BioRender (BioRender.com). (**B**) LV-EF of WT and *Adamts12^–/–^* mice 7 days after MI (*n* = 4 per group). *P < 0.05 (*P* = 0.037), by unpaired *t* test. Selected mice for spatial transcriptomics are marked by arrows. (**C**) Spatial niches in spatial transcriptomic data of WT and *Adamts12^–/–^* mice (*n* = 2 per group). (**D**) Spatial expression of *Adamts12* in WT mice. norm. exp., normalized expression. (**E**) Total *Adamts12* expression stratified by zone and cell type. (**F**) Bar plot of Tangram prediction scores in IZs of WT versus *Adamts12^–/–^* mice after normalization via log_2_ transformation. (**G**) DEGs in WT versus *Adamts12^–/–^* mice in IZ. (**H**) Top enriched Reactome pathways in IZs of *Adamts12*^—/—^ mice based on the DEGs shown in **G**. Str Muscle Contraction, striated muscle contraction. (**I**) PROGENy pathway analysis based on the DEGs shown in **G**. (**J**) Fibroblast subset prediction scores adjusted for the initially imputed Tangram fibroblast prediction score. Spots show the fibroblast subtype with the highest prediction score. Fib, fibroblast; Ifn Fib, interferon fibroblasts; IR Fib, *Atf3*^+^ injury-responsive fibroblasts. (**K**) Tangram-adjusted fibroblast subset prediction scores stratified by zone. (**L**) Tangram-adjusted fibroblast subset prediction scores within the IZ stratified by genotype. ****P < 0.0001, by unpaired *t* test. (**M**) Spatial FeaturePlot of fibroblast 3 (Fib 3) Tangram-adjusted prediction (T.-adj.) scores in WT sample 1 and *Adamts12^–/–^* sample 1. (**N**) Spatial FeaturePlot of IR fibroblast (IR Fib) Tangram-adjusted prediction scores in WT sample 1 and *Adamts12^–/–^* sample 1.

In order to dissect the cellular and molecular cues via which ADAMTS12 controls fibrosis, we next performed spatial transcriptomics (10x, Visium) of WT and *Adamts12^–/–^* mice. On the basis of an ISH time course of *Adamts12* expression in WT mice after MI (Supplemental Figure 4A), we decided to perform spatial transcriptomics of cardiac cross-sections from *Adamts12^–/–^* and WT mice 7 days after MI (Figure 3A). Echocardiography on day 7 again confirmed a preserved EF in *Adamts12^–/–^* mice (Figure 3B). After quality control and Harmony integration (17) (Supplemental Figure 4, B and C), clustering of spatial transcriptomic datasets identified 6 distinct spatial zones, consisting of an ischemic zone (IZ), border zone (BZ), remote border zone (RBZ), healthy myocardium (HM 1, HM 2), and epicardium (Epi) (Figure 3C and Supplemental Figure 4, D and E). As expected, estimation of cell-type composition for each spot using Tangram indicated strong enrichment of fibroblasts and myeloid cells in IZs and BZs, whereas cardiomyocytes and endothelial cells were the predominant cell type in HM (Supplemental Figure 4, F and G). Mapping *Adamts12* revealed a distinct spatial expression pattern localized to IZs and BZs with more than 85% of *Adamts12* expression being assigned to fibroblasts (Figure 3, D and E).

Consequently, focusing on the cellular composition of the IZ highlighted that *Adamts12* deficiency mitigated both cardiomyocyte loss and coinciding fibroblast and myeloid cell expansion (Figure 3F and Supplemental Figure 4G). In line with these findings, differential gene expression analysis (WT vs. *Adamts12^–/–^*, IZ) revealed that loss of *Adamts12* led to downregulation of the fibrosis-defining collagen *Col3a1* gene (Figure 3G and Supplemental Table 8). GO and reactome enrichment analysis further pointed toward improved ventricular remodeling within the IZ of *Adamts12^–/–^* mice with an enrichment of cardiac muscle contraction–associated terms (Reactome striated muscle contraction; GO-BP muscle structure development), ECM remodeling–associated terms (Reactome cell-ECM interactions and elastic fiber formation) (Figure 3H and Supplemental Figure 4H), and RHOH GTPase cycle, a pathway critical in cell polarization and migration (18). Further analysis of IZ pathway activity using PROGENy (19) revealed decreased inflammatory (TNF-α, NF-κB) and JAK/STAT pathway activity in *Adamts12^–/–^* mice (Figure 3I). Reflecting the essential role of *Adamts12* in fibroblast activation and fibrosis, DoRothEA transcription factor analysis confirmed loss of Spi1 transcription factor activity within the IZ of *Adamts12^–/–^* mice (Supplemental Figure 4I), which we initially identified as one of the top perturbed profibrotic transcription factors (8) (rank = 2) in activated Gli1^+^ cells after kidney injury (Supplemental Figure 1D). To our surprise, *Adamts12* deficiency led to increased TGF-β pathway activity, a hallmark pathway in fibrosis (Figure 3I). Supporting these findings, analysis of differentially expressed genes (DEGs) within the IZ did not detect downregulation of common myofibroblast marker genes, such as *Col1a1*, *Fn1*, or *Postn* (3) in *Adamts12^–/–^* mice, suggesting that *Adamts12* did not directly affect myofibroblast differentiation.

On the basis of this observation, we aimed to deconvolute fibroblast activation states in our spatial transcriptomics data. To this end we predicted fibroblast activation states and subsets using a recently published scRNA-Seq fibroblast framework of murine heart failure as a reference (3) and corrected these spatial prediction scores for the initially imputed fibroblast Tangram prediction values. As expected, the homeostatic fibroblast subset 1 (Fib 1) was the most abundantly predicted fibroblast subset within HM 1 and HM 2, while ECM fibroblasts (ECM Fib, commonly referred to as myofibroblasts) were strongly expanded in IZs and BZs (Figure 3, J and K). The fibroblast 3 (Fib 3) subset, which most closely corresponded to epicardial fibroblasts (3), was most commonly predicted to be within the epicardium (Epi). Stratifying fibroblast subset prediction scores within the IZ by genotype revealed that, while loss of *Adamts12* mitigated the expansion of ECM Fib after injury, it predominantly abolished the expansion of *Atf3^+^* injury-responsive fibroblasts (IR Fib) and epicardial Fib 3 within the IZ (Figure 3, L–N, and Supplemental Figure 4, J and K). Supporting the notion that loss of *Adamts12* mitigates early fibroblast activation, we found that the genes *Mt1*, *Mt2*, *S100a8*, and *Serpina3n*, which Forte et al. independently identified as markers of a distinct injury-response fibroblast subset in murine MI (4), were among the top downregulated genes within the IZ of *Adamts12^–/–^* mice (Figure 3G and Supplemental Table 8). In summary, our findings indicate that *Adamts12* controlled epicardial Fib 3 and IR Fib expansion after injury.

Next, we aimed to estimate changes in cellular crosstalk using CrossTalkeR on MI spatial transcriptomic data. In line with our hypothesis that *Adamts12* primarily controls autocrine fibroblast activation, PageRank analysis (WT vs. *Adamts12^–/–^*) of cell types confirmed the strongest downregulation of fibroblast and pericyte PageRank scores (marker of cell-type importance) in *Adamts12^–/–^* mice, while cell-cell interaction analysis corroborated that Adamts12 KO led to a strong downregulation in autocrine fibroblast crosstalk (Supplemental Figure 4, L and M). Focusing on the top differentially expressed ligand-receptor interactions where fibroblasts act as the receptor cell confirmed downregulation of autocrine fibroblast crosstalk via *COL1A1* and *FN1* to ITGAV*ITGB8, but also myeloid-fibroblast crosstalk via *COL1A1* to ITGAV*ITGB8 and *CD36* (Supplemental Figure 4N). Interestingly, we observed an upregulation of fibroblast (ligand) – myeloid (receptor) crosstalk in *Adamts12^–/–^* mice (Supplemental Figure 4M), in line with previous reports that pinpointed *Adamts12* as an important regulator of the innate immune response (15, 16). Further assessment of ligand-receptor interaction by PageRank analysis revealed downregulation of fibroblast *CD200* and myeloid *CD200R* in *Adamts12^–/–^* mice, a critical pathway for regulating excessive immune responses (Supplemental Figure 4O) (20).

**Figure 4:**
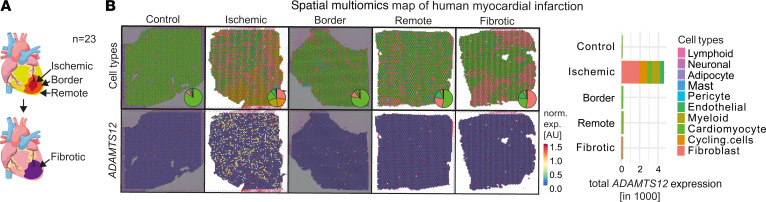
*ADAMTS12* expression in spatial multiomics map of human MI. (**A**) Previously published dataset of human MI with schematic of spatial niche definition. Schematic drawing was created with BioRender.com. (**B**) Cell-type distribution and *ADAMTS12* expression in representative images of each zone. Total *ADAMTS12* expression stratified by zone and predicted cell type.

To extend our findings to human disease, we assessed *ADAMTS12* expression in our recently published spatial transcriptomics dataset of human MI (*n* = 23) (21) (Figure 4, A and B, and Supplemental Figure 5A). Mapping *ADAMTS12* expression across MI zones clearly revealed specific expression in IZs, with fibroblasts being the predominant cell type attributed to *ADAMTS12* expression (Figure 4B).

**Figure 5:**
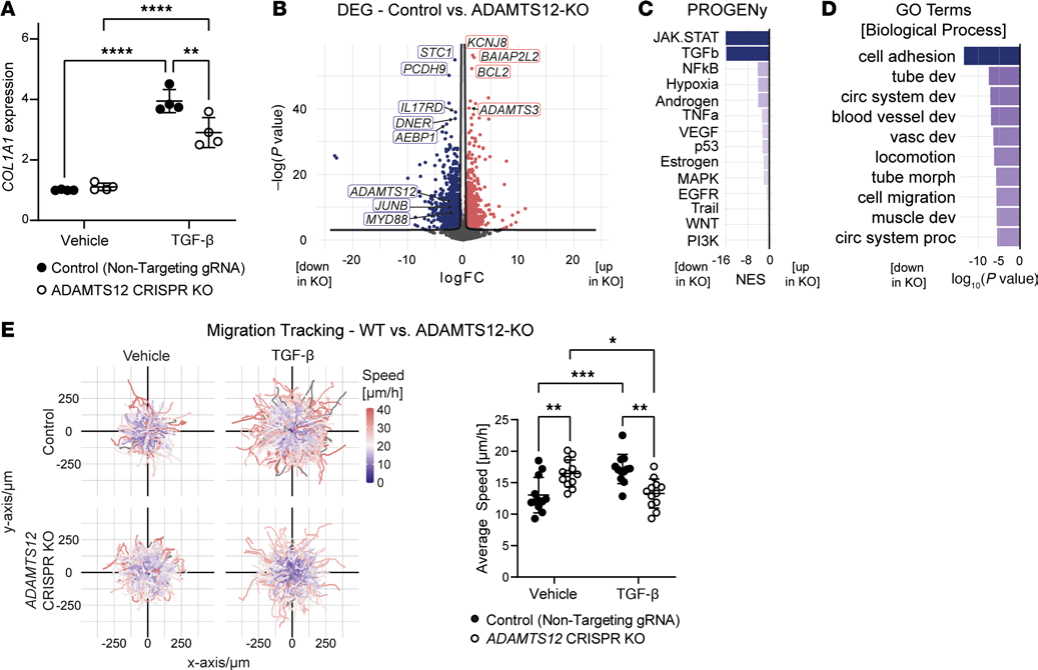
CRISPR/Cas9 KO of *ADAMTS12* in human PDGFRβ^+^ cells. (**A**) *COL1A1* RT-qPCR (*P*_NTG_ _TGF-β_ _vs._ _ADAMTS12-KO_ _TGF-β_ = 0.003) in human PDGFRβ^+^ kidney cells with either CRISPR/Cas9-induced ADAMTS12-KO or NTG RNA transduction after treatment with TGF-β or vehicle (*n* = 4 per group). Results were reproduced in 3 independent experiments. (**B**) Volcano plot of DEGs in WT versus *ADAMTS12-*KO PDGFRβ^+^ cells (*n* = 4 per group). (**C**) PROGENy pathway analysis of the DEGs shown in **B**. (**D**) Top enriched biological process GO terms based on the top downregulated genes in *ADAMTS12-*KO cells shown in **B**. (For abbreviations, see Supplemental Table 10.) (**E**) Trajectory maps of the migration of WT and *ADAMTS12*-KO PDGFRβ^+^ cells after treatment with vehicle or TGF-β. Quantification of the average speed per field of view (*P*_NTG_ _TGF-β_ _vs._ _ADAMTS12-KO_ _TGF-β_ = 0.0016). Results were reproduced in 3 independent experiments. *P < 0.05, **P < 0.01, ***P < 0.001, and ****P < 0.0001, by 2-way ANOVA with Tukey’s post hoc test.

To better understand the signaling pathways via which ADAMTS12 relays fibroblast activation and expansion, we induced *ADAMTS12* KO in immortalized human PDGFRβ^+^ kidney cells using CRISPR/Cas9 gene editing. Successful gene editing was confirmed on a genomic and transcriptomic level (Supplemental Figure 6, A and B). While KO of *ADAMTS12* did not affect *COL1A1* expression at baseline, it blunted *COL1A1* expression in response to TGF-β (Figure 5A). To more comprehensively assess the effect of *ADAMTS12* on fibroblast activation, we next performed bulk RNA-Seq of *ADAMTS12*-KO and control (nontargeting sgRNA–transduced) cells. After quality control, PCA showed clear separation of control and *ADAMTS12*-KO samples (Supplemental Figure 6, C and D). Subsequent DEG analysis validated our earlier findings within the IZ of *Adamts12^–/–^* mice, demonstrating downregulation of previously identified profibrotic genes, i.e., *JUNB* (13, 14) and *MYD88* (22, 23) (Figure 5B and Supplemental Table 9). Further corroborating our earlier findings, analysis of PROGENy pathway activity revealed JAK/STAT signaling as the top downregulated pathway after *ADAMTS12* KO, emphasizing the effect of *ADAMTS12* on fibroblast JAK/STAT signaling (Figure 5C). To better understand the functional consequences of a loss of *ADAMTS12* in PDGFRβ^+^ cells, we next performed GO-BP enrichment analysis. Interestingly, enrichment analysis identified cell adhesion, cell migration and locomotion among the top downregulated GO terms in *ADAMTS12-*KO cells (Figure 5D). Fibroblast migration is deemed a crucial step in fibroblast activation that enables relocation to sites of tissue injury (24). As our previous data had indicated an abrogated expansion of IR Fib in *Adamts12^–/–^* mice (Figure 3, L–N, and Supplemental Figure 4, J and K), we questioned whether *ADAMTS12* controls fibroblast migration. To investigate cell migration, we performed live cell imaging of control and *ADAMTS12*-KO cells over 24 hours (Supplemental Figure 6E and Supplemental Video 2). While *ADAMTS12*-KO cells migrated faster at baseline, loss of *ADAMTS12* severely blunted the migratory response to TGF-β, validating ADAMTS12 as a crucial autocrine checkpoint for migration of activated fibroblasts (Figure 5E).

**Figure 6:**
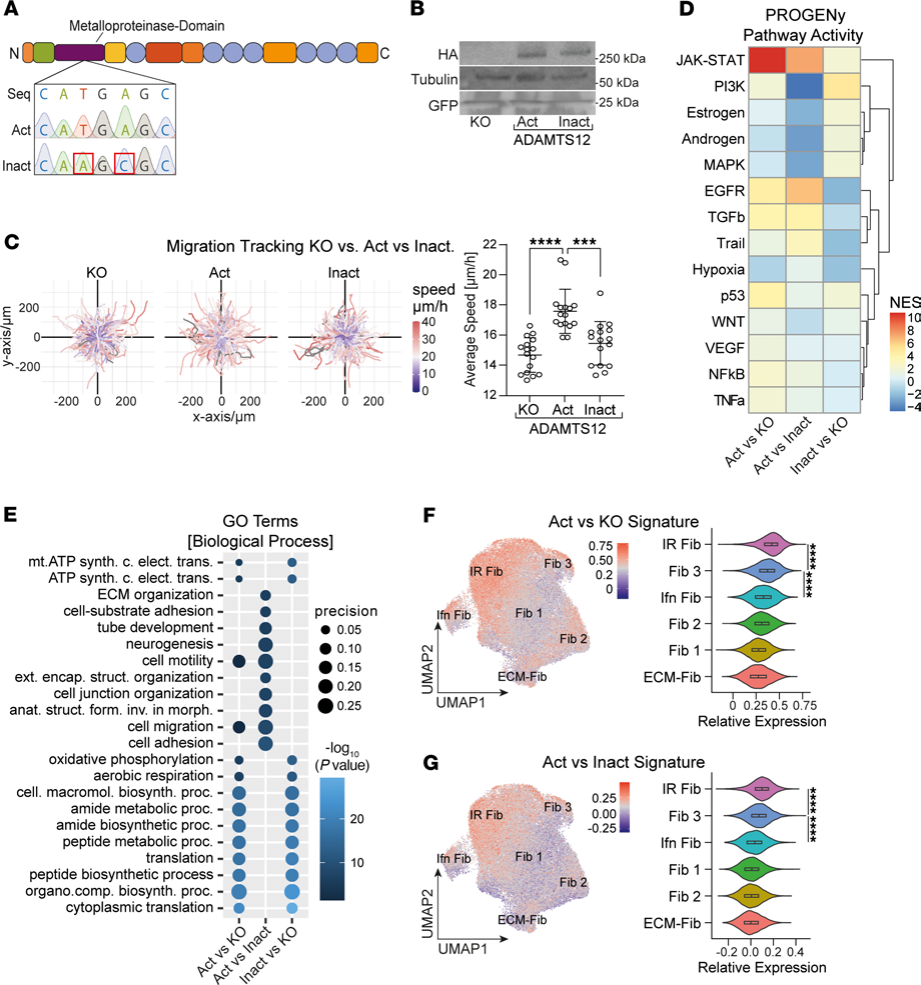
Rescue of *ADAMTS12* KO by overexpression of catalytically active or inactive ADAMTS12. (**A**) Sequencing of *ADAMTS12* expression plasmids with a WT active (Act) or a mutated inactive (Inact) catalytic domain. Seq, original *ADAMTS12* sequence; Act, active catalytic domain; Inact, inactive catalytic domain. (**B**) Western blot for HA, tubulin, and GFP in *ADAMTS12*-KO, active or inactive ADAMTS12-expressing PDGFRβ^+^ cells. (**C**) Trajectory maps and quantification of the average migration speed of *ADAMTS12*-KO and active and inactive ADAMTS12-expressing PDGFRβ^+^ cells (*n* = 16 per group, p_KO_ _vs._ _Act_< 0.0001, *P*_Act_ _vs. Inact_ = 0.0002, by ordinary 1-way ANOVA). Results were reproducible in 3 independent experiments. (**D**) PROGENy pathway analysis based on the DEGs shown in Supplemental Figure 6, G–I. ActvsKO, catalytically active ADAMTS12 expression versus *ADAMTS12*-KO; ActvsInact, catalytically active versus inactive ADAMTS12 expression; InactvsKO, catalytically inactive ADAMTS12 expression versus *ADAMTS12*-KO. (**E**) Top enriched biological process GO terms based on the top upregulated genes shown in Supplemental Figure 6, G–I. Comparisons are described in **D** (for abbreviations, see Supplemental Table 14). (**F**) ADAMTS12 active versus KO signature (Act vs. KO) in a scRNA-Seq framework of murine cardiac fibroblasts in heart failure. (**G**) ADAMTS12 active versus inactive signature (Act vs. Inact) in the above dataset. ***P < 0.001 and ****P < 0.0001. For **C**, **F**, and **G**, a 1-way ANOVA with Tukey’s post hoc test was performed.

To determine whether catalytically active ADAMTS12 affects cell migration as a consequence of cleaving ECM, we decided to rescue *ADAMTS12-*KO in PDGFRβ^+^ cells by inducing expression of either HA-tagged catalytically active ADAMTS12 (Act) or inactive ADAMTS12 (Inact, H465Q-E466A, ref. 25) using a retroviral expression system (Figure 6A). RT-qPCR confirmed silencing of Cas9 (Supplemental Figure 7A), excluding the possibility of de novo CRISPR/Cas9 gene editing, whereas HA Western blotting confirmed ADAMTS12 expression (Figure 6B). In addition, catalytically active or inactive ADAMTS12 expression was validated in an in vitro digestion assay with the ADAMTS12-substrate cartilage oligomeric matrix protein (COMP) (Supplemental Figure 7, B and C). Corroborating our previous findings, assessment of the migratory capacity in Matrigel ECM demonstrated that expression of active, but not inactive, ADAMTS12 rescued cell migration (Figure 6C).

To again profile the effect of ADAMTS12 in a comprehensive manner, we performed bulk RNA-Seq of *ADAMTS12*-KO, active ADAMTS12–, and inactive ADAMTS12–expressing PDGFRβ^+^ cells. Quality control and PCA revealed 1 outlier (Inact 4), which was excluded from further analysis (Supplemental Figure 7, D–F). Reflecting our prior observations, differential gene expression analysis again found upregulation of previously identified profibrotic genes (*JUNB, MYD88*) in active ADAMTS12–expressing cells, both in comparison with ADAMTS12-KO and inactive ADAMTS12–expressing cells (Supplemental Figure 7, G–I, and Supplemental Tables 11–13). Of note, analysis of metalloprotease gene expression detected no compensatory upregulation of *ADAMTS7*, the closest homolog to ADAMTS12 (Supplemental Figure 7J). Further validating our prior findings, PROGENy pathway analysis identified JAK/STAT as the top upregulated pathway activity in active ADAMTS12–expressing cells, both in comparison with *ADAMTS12*-KO and inactive ADAMTS12–expressing cells, confirming that catalytically active ADAMTS12 drives JAK/STAT signaling (Figure 6D). Similarly, enrichment analysis again found migration-associated terms (cell motility, migration, and adhesion) and ECM organization terms (cell-substrate adhesion, ECM organization) as the top enriched GO-BP terms in active versus inactive ADAMTS12–expressing cells, confirming that catalytically active ADAMTS12 shapes ECM composition and drives cell migration (Figure 6E).

Finally, we questioned whether expression of catalytically active ADAMTS12 in PDGFRβ^+^ cells drives IR Fib polarization. To this end, we scored fibroblasts from the previously used scRNA reference dataset of fibroblasts in murine heart failure (3) based on their expression of genes upregulated in active ADAMTS12–expressing cells (in comparison with ADAMTS12-KO or inactive ADAMTS12–expressing cells). This comparison revealed the strongest enrichment of both Act versus KO and Act versus Inact signatures in IR Fib, followed by Fib 3, reiterating that ADAMTS12 drives IR Fib polarization (Figure 6, F and G).

To validate our findings on JAK/STAT signaling in vivo, we next performed Western blotting of STAT proteins in *Adamts12^–/–^* and WT mice after sham or UUO surgery. Indeed, densitometry confirmed that loss of *Adamts12* led to significantly decreased STAT1 and STAT2 protein levels and a trend for decreased STAT3 protein levels after UUO (Supplemental Figure 8, A–D), consistent with the notion that *ADAMTS12* drives JAK/STAT signaling in vitro and in vivo. Similarly, PROGENy pathway activity inference in interstitial cells of the human KPMP scRNA-Seq kidney dataset revealed that increased *ADAMTS12* expression in myofibroblasts and adaptive fibroblasts after AKI and CKD was associated with increased JAK/STAT pathway activity (Supplemental Figure 8E).

Next, we tested whether a broad-spectrum MMP inhibitor, Batimastat, could inhibit the effect of ADAMTS12 on IR Fib activation. We reasoned that inhibition of MMPs should abrogate gene expression driven by compensatory upregulation of MMPs. However, Batimastat did not have a significant effect on *ATF3*, *FOS*, or *DHRS7* expression (markers of IR Fib) in ADAMTS12-expressing cells (Supplemental Figure 8F). These findings indicate that ADAMTS12 has a unique effect on IR Fib activation that cannot be targeted by broad-spectrum MMP inhibition.

**Figure 7:**
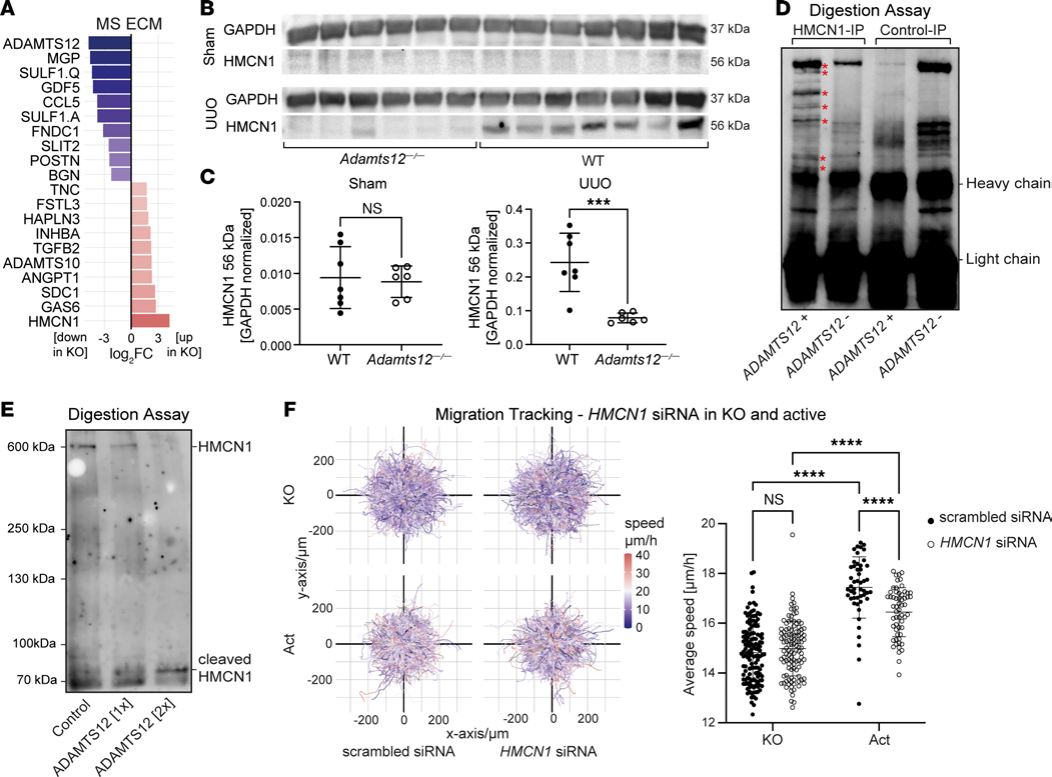
HMCN1 is a substrate of ADAMTS12 that facilitates ADAMTS12-induced migration. (**A**) log_2_FC of the top up- and downregulated proteins in ECM of WT versus *ADAMTS12*-KO PDGFRβ^+^ cells (*n* = 3 per group). (**B**) Western blot of lower-weight HMCN1 peptides (56 kDa) in kidneys from WT and *Adamts12^–/–^* mice after sham or UUO surgery (*Adamts12^–/–^* *n* = 6, WT *n* = 7). (**C**) Quantification of band density via a 2-tailed unpaired *t* test. (**D**) Digestion of HMCN1 or control IP lysates with ADAMTS12 or vehicle and subsequent detection of HMCN1 via Western blotting. (**E**) Digestion of supernatant from HMCN1-expressing RPE cells with vehicle or 2 concentrations of ADAMTS12 (1× = 90 ng, 2× = 180 ng). Detection of HMCN1 by Western blotting. (**F**) Trajectory maps of the migration of *ADAMTS12*-KO and active ADAMTS12-overexpressing PDGFRβ^+^ cells treated with scrambled or HMCN1 siRNA. Quantification of the average speed per field of view (KO/scrambled siRNA *n* = 136, KO/HMCN1 siRNA *n* = 112, Act/scrambled siRNA *n* = 48, Act/HMCN1 siRNA *n* = 57, *P*_KO_ _scrambled_ _siRNA_ _vs._ _KO_ _HMCN1_ _siRNA_ = 0.69, *P*_Act_ _scrambled siRNA_ _vs._ _Act_ _HMCN1_ _siRNA_<0.0001, by 2-way ANOVA with Tukey’s post hoc test). ***P < 0.001 and ****P < 0.0001.

Last, we aimed to identify the substrate through which ADAMTS12 controls fibroblast migration, activation, and, ultimately, fibrosis. In vivo, Western blotting detected a distinct loss of the known substrates CTGF and COMP in *Adamts12^–/–^* mice after UUO (Supplemental Figure 9, A–D), whereas the substrates COMP, CTGF, ACAN, and NCAN were not detected in the MS UUO data. These results suggest that neither CTGF nor COMP is a relevant substrate of ADAMTS12 in fibrosis, as the latter should be associated with an enrichment in *Adamts12^–/–^* mice. On the basis of these results, we hypothesized that ADAMTS12 controls fibroblast migration and activation via a hitherto unknown substrate. To assess ADAMTS12 substrates in activated fibroblasts in an unbiased manner, we therefore performed MS analysis of ECM secreted by TGF-β–stimulated WT or *ADAMTS12*-KO PDGFRβ^+^ cells (Figure 7A and Supplemental Table 15). In line with bulk RNA-Seq data, enrichment analysis based on significantly downregulated proteins revealed strong enrichment of the GO-BP terms “cell migration,” “adhesion,” and “motility” (Supplemental Figure 9E). In contrast, we did not detect significant shifts in ECM composition (Supplemental Figure 9F). However, as we isolated ECM and depleted cells for MS, these data were only suitable for quantifying shifts in ECM composition, but not overall ECM abundance. Our assessment of known ADAMTS12 substrates in ECM MS data again detected no significant differences in CTGF, COMP, ACAN, or NCAN levels (Supplemental Table 13). Interestingly, the most abundant protein in *ADAMTS12*-KO ECM was the fibulin HMCN1 (Figure 7A). HMCN1 has been previously identified as a critical part of basement membranes, expressed inter alia in kidney vasculature, that serves as a tethering point for cells to membranes (26, 27). Although HMCN1 Western blotting of WT and *Adamts12^–/–^* mice showed no differences after sham surgery, we found a strong enrichment of a smaller-sized HMCN1 fragment (~56 kDa) in WT mice after UUO, suggestive of differential cleavage in fibrosis (Figure 7, B and C). Of note, given the large size of uncleaved HMCN1 (~600 kDa), we were not able to detect the larger-sized, uncleaved HMCN1 in Western blots.

On the basis of these findings, we hypothesized that ADAMTS12-mediated cleavage of HMCN1 initiates fibroblast migration and activation. To first confirm cleavage of HMCN1 by ADAMTS12, we performed HMCN1 IP of *ADAMTS12*-KO cells using an HMCN1 antibody or an isotype rabbit antibody as a control, and subsequently incubated IP lysates with active ADAMTS12 or vehicle for 12 hours. HMCN1 Western blotting detected distinct lower-weight HMCN1 bands after ADAMTS12 digestion of HMCN1 IP lysates, but not with vehicle digestion, and also not in control IP lysates (Figure 7D), indicative of HMCN1 cleavage by ADAMTS12. As we were unable to detect full-length HMCN1 (~600 kDa), we next aimed to confirm our results in a second assay. Here, we digested the supernatant of HMCN1-expressing retinal pigment epithelial (RPE) cells with either control or 90 ng or 180 ng ADAMTS12. As a control, we confirmed the digestion of recombinant COMP by ADAMTS12 (Supplemental Figure 9H). Western blotting confirmed loss of uncleaved 600 kDa HMCN1 and the concomitant emergence of cleaved HMCN1 peptides at approximately 70 kDa with increasing concentrations of ADAMTS12, corroborating the cleavage of HMCN1 by ADAMTS12 (Figure 7E).

Finally, we investigated whether cleavage of HMCN1 by ADAMTS12 mediates fibroblast migration. To this end, we inhibited *HMCN1* expression in vitro using siRNA in *ADAMTS12*-KO and active ADAMTS12–expressing cells. Successful *HMCN1* inhibition was confirmed in a parallel experiment in which cells were harvested for RT-qPCR (Supplemental Figure 8I). We hypothesized that (a) either uncleaved HMCN1 anchors mesenchymal cells in the perivascular niche, or (b) cleaved HMCN1 peptides induce migration and fibroblast activation. In both scenarios, cleavage of HMCN1 by ADAMTS12 would induce activation and migration of mesenchymal cells. Indeed, knockdown of HMCN1 in ADAMTS12-overexpressing cells significantly inhibited migration (Figure 7F). This observation is in line with the hypothesis that cleaved HMCN1 peptides facilitate mesenchymal cell migration and activation, as reduced HMCN1 expression leads to lower HMCN1 cleavage by ADAMTS12. Interestingly, we simultaneously detected a robust, but nonsignificant, acceleration of *ADAMTS12*-KO cells after HMCN1 knockdown, in line with the notion that HMCN1 anchors cells (Figure 7F). We hypothesize that uncleaved and cleaved HMCN1 might serve redundant roles as downstream mediators of ADAMTS12-induced cell migration. Taken together, our results pinpoint ADAMTS12 as a critical checkpoint in fibrosis that controls fibroblast migration via cleavage of a what we believe to be previously unknown substrate, HMCN1.

## Discussion

In this study we discovered a conserved upregulation of the metalloprotease *Adamts12* in fibroblasts during the active stages of fibrogenesis across organs and species. Strikingly, we found that genetic loss of *Adamts12* in mice mitigated renal and cardiac fibrosis and preserved cardiac function after injury. Leveraging spatial transcriptomics in murine MI, we identified *Adamts12* as a driver of IR fibroblast activation and expansion. By generating catalytic active and inactive ADAMTS12 mutants in vitro, we confirmed that catalytically active ADAMTS12 drives fibroblast migration and polarization toward an IR fibroblast phenotype, defined by elevated JAK/STAT signaling. Last, we identified HMCN1 as, in our view, a novel ADAMTS12 substrate. HMCN1 perturbation in vitro confirmed that ADAMTS12-mediated HMCN1 cleavage controls cell migration.

Upon injury, fibroblasts are thought to detach and migrate from their perivascular niche to sites of injury, where they sustain tissue architecture via ECM deposition and, when uncontrolled, drive fibrosis (5, 7, 28). Although this concept stands unchallenged, the process of fibroblast migration is poorly understood, with few studies providing direct evidence for fibroblast detachment and migration in vivo (5, 7, 29). Here, we provide evidence that ADAMTS12 not only drives cell migration, but also controls JAK/STAT and TGF-β pathway activity as well as an injury-responsive–like fibroblast activation. On the basis of these observations, we hypothesize that ADAMTS12 serves as an autocrine switch that controls the initiation of fibroblast activation, including detachment and migration from the perivascular niche.

TGF-β signaling is considered the key node that reconciles different signaling pathways to drive myofibroblast differentiation and ultimately fibrosis (30). In vitro, we show that loss of *ADAMTS12* blunted TGF-β pathway activity as well as the effects of TGF-β signaling on migration, while ADAMTS12 expression, without additional TGF-β stimulation, was sufficient to rescue TGF-β pathway activity (Figure 5E and Figure 6C). These findings indicate that ADAMTS12 acted downstream of TGF-β, fine-tuning fibroblast migration and activation toward a distinct IR fibroblast subset. Of note, while further research is needed to dissect the nuances of ADAMTS12 as a downstream mediator of TGF-β signaling, we hypothesize that the observed increase in TGF-β pathway activity in spatial transcriptomics data (Figure 3I) of *Adamts12^–/–^* mice after MI is a compensatory feedback mechanism to compensate for decreased collagen deposition.

Transcriptomics profiling revealed that ADAMTS12 drives a distinct IR fibroblast signature characterized by high JAK/STAT signaling in vitro and in vivo. These findings are reminiscent of profibrotic cues identified in myeloproliferative neoplasms, in which aberrant JAK/STAT signaling leads to fibrotic remodeling of the hematopoietic niche (31, 32). Interestingly, abrogation of JAK/STAT-mediated inflammatory loops has been shown to reduce fibrosis in this context (33, 34) and could potentially serve as a conceptual blueprint for targeting fibrosis beyond hematological neoplasms. Our data indicate that inactive ADAMTS12 also elevated JAK/STAT signaling in comparison with *ADAMTS12-*KO cells, albeit to a much lower extent than catalytically active ADAMTS12. Taking into consideration that the 2 point mutations strongly reduce but do not abrogate ADAMTS12 catalytic activity, it is possible that expression of inactive ADAMTS12 either drives JAK/STAT signaling via residual catalytic activity or that ADAMTS12 drives JAK/STAT signaling via both catalytic-dependent and -independent pathways. While the comparison of JAK/STAT pathway activity in catalytically active to inactive ADAMTS12 cells corroborates that active ADAMTS12 is the primary driver of JAK/STAT signaling, further research is needed to distinguish potential pleiotropic effects of ADAMTS12. Several studies have investigated the role of ADAMTS12 in the context of cancer development. In line with our findings, previous studies have linked ADAMTS12 expression to cancer-associated fibroblasts (35), while Lie et al. identified ADAMTS12 as a driver of migration (36). Similar to these findings, we report an expression pattern of *ADAMTS12* that was restricted to activated fibroblasts, which drove fibroblast migration in an autocrine manner. In contrast to prior studies that reported an exaggerated inflammatory response with loss of *Adamts12* (15, 16), we did not find aberrant inflammation in *Adamts12^–/–^* mice in the context of fibrosis (Figure 2B and Supplemental Figure 3, A, H, and I). In vitro, PROGENy inferred decreased TNF and NF-κB pathway activity in human PDGFRβ cells with KO of *ADAMTS12*, indicating that loss of *Adamts12* did not drive inflammation in fibroblasts at baseline (Figure 5C and Figure 6D). Similarly, scRNA-Seq kidney and spatial transcriptomics heart data indicated that (myo)fibroblasts were the major source of *ADAMTS12* in homeostasis and fibrosis (Figure 1, E–J, Figure 3, D and E, Figure 4, A and B, Supplemental Figure 1, E–L, Supplemental Figure 2, A–F, and Supplemental Figure 4A). As our data do not allow conclusions regarding the role and expression of ADAMTS12 in other contexts, we therefore consider ADAMTS12 to not be a driver of aberrant inflammation in the context of fibrosis. Interestingly, cell-cell interaction analysis with CrossTalkeR revealed an increase in fibroblast (ligand)–myeloid (receptor) crosstalk with genetic loss of *Adamts12* (Supplemental Figure 4, L and M). Moreover, PageRank analysis identified fibroblast-expressed CD200 and myeloid-expressed CD200R, the corresponding CD200 receptor, among the top downregulated ligands/receptors in *Adamts12^–/–^* mice (Supplemental Figure 4O). CD200/CD200R was previously identified as a critical signaling pathway for attenuation of immune responses. As such, it is possible that loss of *Adamts12* with subsequent loss of CD200/CD200R fibroblast-macrophage signaling leads to a dysfunctional attenuation of inflammation in response to other stimuli, i.e., in infection and autoimmune disease, but not fibrosis. This hypothesis would reconcile our findings with previously reported ADAMTS12 phenotypes of aberrant inflammation.

In summary, our findings identify ADAMTS12 as a critical checkpoint during active fibrotic remodeling, licensing injury-responsive fibroblast activation and migration. Taking into account the expression pattern of ADAMTS12, with strongest upregulation at approximately 1–2 weeks after injury, we hypothesize that ADAMTS12 is critical for the initial activation and subsequent expansion of fibroblasts during the incipience of fibrosis.

## Methods

### Sex as a biological variable.

Our study examined male and female animals, and similar findings are reported for both sexes.

### Mice.

Gli1CreERt2 (JAX stock 007913) and Rosa26tdTomato (JAX stock 007909) were purchased from The Jackson Laboratory. *Adamts12^–/–^* mice were developed by C. Lopez-Otin (25). Genotyping of mice was performed by PCR. Mice (1–5 mice) were housed together with unlimited access to water and food on a 12-hour light/12-hour dark cycle, at 20°C under specific pathogen–free conditions at RWTH Aachen University. As reported by Hour et al. previously (25), *Adamts12^–/–^* mice were normally fertile with normal lifespans and no overt phenotype. In addition, 6- to 8-month-old *Adamts12^–/–^* mice showed no baseline phenotype compared with WT mice in H&E stainings of major organs (heart, lung, liver, kidney, muscle, spleen), as evaluated by a trained pathologist (Supplemental Figure 10).

### UUO.

For inducible fate tracking, 8-week-old male Gli1CreER tdTomato mice (*n* = 3) received tamoxifen 3 times by gavage (10 mg p.o.) followed by a washout period of 21 days. Gli1CreER tdTomato mice as well as *Adamts12^–/–^* mice (*n* = 6; 2 females, 4 males; 20–24 weeks of age) and WT (*n* = 7; 4 females, 3 males; 18–24 weeks of age) underwent UUO surgery and contralateral sham surgery as previously described (37). In brief, mice were anesthetized with 120 mg/kg BW ketamine and 16 mg/kg BW xylazine. After induction of narcosis, the left flank was incised and the left ureter ligated at the level of the lower pole with two 5.0 sutures (Mersilene). For the sham surgery, an isolated flank incision was placed on the contralateral right flank of the same animal. For analgesia, 200 mg/kg BW in 100 μL NaCl solution was administered subcutaneously 30 minutes prior to surgery. After surgery, metamizole (1.25 mg/mL) was added to the drinking water in combination with 1% sucrose for 72 hours. Mice were subsequently sacrificed via cardiac puncture following ketamine/xylazine narcosis. A small incision was made in the right ventricle, after which the left ventricle was perfused with 20 mL of 4°C PBS to remove residual blood from the vasculature.

### MI.

To compare cardiac fibrosis between WT and *Adamts12^–/–^* mice 56 days after MI, 11- to 17-week-old *Adamts12^–/–^* mice underwent left anterior descending coronary artery ligation (*n* = 11; 6 females, 5 males) or sham surgery (*n* = 9; 4 females, 5 males). As specifically requested by the regional authorities (LANUV-NRW, Germany), we had to use the same group of WT sham- and MI-operated mice, as in our previous study, in which WT and *Cxcl4^–/–^* animals were subjected to sham or MI operation (38). For Picrosirius red stainings, heart sections from WT and *Adamts12^–/–^* mice were stained anew as described in the Supplemental Methods. For echocardiography, data on the WT mice was reanalyzed together with echocardiographic data on *Adamts12^–/–^* mice by a blinded trained professional, as described below. For Visium spatial transcriptomics experiments, 8-week-old female *Adamts12^–/–^* (*n* = 4) and WT (*n* = 4) mice were used. MI surgeries were performed as previously described (39). Mice were anesthetized using 2%–2.5% isoflurane. For analgesia, metamizole (200 mg/kg BW in 100 μL NaCl solution) was administered subcutaneously 30 minutes prior to surgery in addition to local analgesia by subcutaneous and intercostal injection of bupivacaine (2.5 mg/kg BW). After induction of narcosis, mice were intubated and ventilated with oxygen by mouse respirator (Harvard Apparatus). A Left thoracotomy was performed, and MI was induced by ligature of the left anterior descending artery using 0/7 silk (Seraflex, IO0517IZ). For the sham surgery, an isolated left thoracotomy without left anterior descending coronary artery ligation was performed. After ligation, the ribs, muscle layer, and skin incision were closed via suture, and Metamizole was administered for 3 days via the drinking water (1.25% mg/mL, 1% sucrose). Mice were sacrificed 7 or 56 days after MI, as described above.

### Echocardiography.

Echocardiography was performed 2 days before, 28 after, and 56 days after surgery for the initial MI experiment. For spatial transcriptomics, echocardiography was performed 7 days after MI surgery. Imaging was performed by a trained professional on a small animal ultrasound imager (Vevo 2100 and MX550D transducer, FUJIFILM, Visualsonics). Mice were anesthetized with isoflurane during the procedure. To assess the ejection fraction, the Simpson method was used by manual image annotation using VevoLab Software (Fujifilm VisualSonics).

### FACS and Affymetrix microarray.

Kidneys were minced into small, approximately 1 mm^2^ slices and transferred to a digestion solution containing 25 μg/mL Liberase TL (Roche) and 50 μg/mL DNase (MilliporeSigma) in RPMI (Gibco, Thermo Fisher Scientific) in a C-tube (Miltenyi Biotec). Tissue was processed on a gentleMACS (Miltenyi Biotec) using the program Spleen 4 and subsequently digested for 30 minutes at 37°C while shaking at 300 rpm before processing again on the gentleMACS using the Spleen 4 program. The resulting suspension was passed through a 70 μm cell strainer (Falcon), washed with 45 mL cold PBS, and centrifuged for 5 minutes at 500*g* at 4°C. Cells were counted using a hemocytometer with trypan blue staining. Overall cell viability was greater than 80%. Isolated cells were resuspended in FACS buffer (1% FBS in PBS) on ice at a final concentration of 1 × 10^7^ cells/mL and filtered using a 40 μm cell strainer (Falcon). Live, single cells were isolated by FACS using a FACSAria II instrument (BD) and gated for Gli1-tdTomato^+^, DAPI^–^ cells. On average, it took 5–6 hours from obtaining the biopsies to preparing the single-cell suspensions. After Gli1 cell isolation, an Affymetrix GeneChip Mouse Genome 430 2.0 Array was performed according to the manufacturer’s instructions.

### RNA-ISH staining and image analysis.

ISH was performed using the RNAscope Multiplex Detection Kit V2 (RNAscope, 323100) on formalin-fixed, paraffin-embedded tissue following the manufacturer’s protocol. Target retrieval was performed for 30 minutes. The following probes were used for the RNAscope assay: Mm-Adamts12 400531-C1, Mm-PDGFRβ 411381-C3, Hs-PDGFRβ 548991-C1, Hs-COL1α1 401891-C2, and Hs-ADAMTS12 507691-C3. For the UUO and MI time course, 7 images were acquired (for kidney cortex areas only) in a randomized fashion, using the ×40 objective of a Nikon A1R confocal microscope. Stained spots were counted after splitting images into their original channels and background subtraction (rolling ball radius = 10.0 pixels) using ImageJ (NIH) (37, 40). For analysis of the human tissue microarray, 3 *Z*-stacks were acquired from the renal cortex in randomly selected areas. Using ImageJ (37, 40), *Z*-stacks were overlaid as *Z*-Projects and channels were split. Cells were segmented and classified by a trained algorithm using the object classification workflow of ilastik (41).

### RT-qPCR.

For tissue samples, snap-frozen tissue was shredded using a Mixer Mill, and RNA was extracted using the RNeasy Mini Kit (74106, QIAGEN) according to the manufacturer’s instructions. For cell culture samples, cells were lysed and homogenized using QIAshredder spin columns (79656, QIAGEN), and RNA was isolated as described above. Total RNA (200 ng) was reverse-transcribed with the High-Capacity cDNA Reverse Transcription Kit (4368813, Applied Biosystems). RT–qPCR was performed in duplicate using SYBR Green Master Mix (Bio-Rad) and the CFX Connect Real-Time System (Bio-Rad). The cycle protocol was 95°C for 2 minutes, 40 cycles of 5 seconds at 95°C, and 30 seconds at 60°C, followed by a final 5 seconds at 95°C. *Gapdh* served as a housekeeping gene. Data were analyzed using the 2^–Ct^ method. The primers used are listed in Supplemental Table 16.

### Immunofluorescence staining and image analysis.

Cryosections (5 μm) were blocked for 30 minutes with 10% BSA followed by a 1-hour incubation with the primary antibody. After washing 3 times for 5 minutes in PBS and subsequent incubation with secondary antibodies for 30 minutes, the slides were washed, counterstained with DAPI (Roche, 1:10,000), and mounted with Immu-Mount (9990402, Epredia). For each sample, 4 images of the renal cortex were acquired using the ×40 objective of a Nikon A1R confocal microscope in a randomized fashion. For quantification, images were split into channels, and the positive area was determined by spectral thresholding using ImageJ (37, 40). The following antibodies were used: anti–mouse PDGFRβ (ab32570, 1:100, Abcam), anti–mouse CD31 (553370, 1:200, BD Biosciences), AF488 donkey anti-rabbit (711-545-152, 1:200, Jackson ImmunoResearch), and AF647 donkey anti-rat (712-605-153, 1:200, Jackson ImmunoResearch).

### IHC staining.

After deparaffinization of 2 μm paraffin sections, antigen unmasking was performed by heating sections 3 times for 5 minutes in citric acid–based antigen unmasking solution (H-3300, Vector Laboratories). Slides were blocked by incubation with 3% hydrogen peroxide for 3 minutes and avidin/biotin (VEC-SP-2001, Vector Laboratories) for 10 minutes each, followed by a 1-hour incubation with the primary antibody, washing 3 times in PBS, and subsequent incubation with the secondary antibody. Detection was carried out using the DAB Substrate Kit (SK-4100, Vector Laboratories). Slides were counterstained for hematoxylin, dehydrated, and mounted. For each sample, 7 representative images of the renal cortex were acquired using the ×40 objective of a bright-field microscope (BZ-9000, Keyence). The following antibodies were used: anti–mouse Col1 (1310-01, 1:100, SouthernBiotech) and biotinylated horse anti-goat (BA-9500, 1:300, Vector Laboratories).

### Spatial transcriptomics.

Spatial gene expression profiling (Visium, 10X Genomics, PN-1000187), including library construction of 10 μm OCT-embedded murine heart sections, was performed according to the manufacturer’s instructions and as described previously (21). The optimal lysis duration was determined to be approximately 18 minutes. Bright-field images were taken using a FRITZ scanner microscope. After library construction, libraries were sequenced on a NovaSeq 6000 System (Illumina) as recommended by 10X Genomics.

### Analysis of a reference spatial transcriptomics human MI dataset.

Data on *ADAMTS12* expression and cell-type annotation were retrieved from a previously published dataset by our group (21) and plotted using Seurat’s SpatialDimPlot and SpatialFeaturePlot function. For *ADAMTS12* quantification, *ADAMTS12* expression was summed by zone and cell type, as described above.

### CRISPR/Cas9 vector construction, virus production, and transduction.

CRISPR/Cas9 vector construction, virus production, and transduction were performed as described before (2). In summary, *ADAMTS12*-specific guide RNA (forward 5′-CACCGAACATCATAGATCACTCCGG-3′; reverse 5′-AAACCCGGAGTGATCTATGATGTTC-3′) and a control nontargeting guide (NTG) RNA were subcloned into pL-CRISPR.EFS.GFP (57818, Addgene) using BsmBI restriction digestion. For lentiviral particle production, HEK293T cells were cotransfected with the generated pL-CRISPR.EFS.GFP NTG or *ADAMTS12* plasmids and the packaging plasmids (psPAX2: 12260, Addgene; pMD2.G: 12259, Addgene) using TransIT-LT (Mirus). Forty-eight hours after transfection, viral supernatant was harvested, clarified by centrifugation, and supplemented with 10% FCS and polybrene (H9268-5G, MilliporeSigma; final concentration of 8 μg/mL). PDGFRβ^+^ cells were subsequently transduced by a 48-hour incubation with the viral supernatant. To generate single clones, eGFP^+^ cells were single-cell sorted into a 96-well plate. To determine specific mutation events, single-cell colonies were assessed by PCR amplification of the *ADAMTS12* CRISPR target site and Sanger sequencing of the PCR product as well as RT-qPCR analysis for *ADAMTS12* expression. Predicted potential off-target effects for *INTS9*, *TTC41P*, and *ARHGAP* were excluded by PCR amplification and Sanger sequencing of predicted potential off-target sites.

### Bulk RNA library construction.

RNA was extracted as described above. For cDNA and library construction of WT versus *ADAMTS12*-KO libraries, we used the MGIEasy RNA Library Prep Set (MGI, 1000006384) in combination with the MGIEasy rRNA Depletion Kit (MGI, 1000005953) according to the manufacturer’s instructions. After library construction and quality control using the AgilentTapeStation System, libraries were sequenced on a DnbSeq-G400 system, targeting a read depth of 25,000,000 reads/library. For cDNA and library construction of *ADAMTS12*-KO versus active or inactive ADAMTS12-expressing samples, we used the NEBNext Ultra II Directional RNA Library Prep Kit (New England Biolabs [NEB], E7760L) according to the manufacturer’s instructions. After quality control on an AgilentTapeStation, samples were sequenced on an Illumina NovaSeq, targeting a read depth of 25,000,000 reads/library.

### Migration tracking.

Cells were seeded in a flat, clear-bottomed, 96-well plate (89626, ibidi) covered with Matrigel (11553620, Corning) in DMEM containing 5% FCS, 1% penicillin/streptomycin, and 0.2% MycoZap (Lonza, catalog VZA-2022). For the comparison of WT and ADAMTS12-KO cells, after 24 hours of incubation in starvation medium with 0.5% FCS, 50% confluent cells were stimulated with vehicle or 10 ng/mL TGF-β in CO_2_-independent medium (18045054, Gibco, Thermo Fisher Scientific). For the analysis of active or inactive ADAMTS12-overexpressing cells and the siRNA knockdown of HMCN1, no TGF-β stimulation was performed. After 24 hours of stimulation, cell autofluorescence was captured every 10 minutes for 18–24 hours in a 37°C incubation chamber with a Nikon A1R confocal microscope. Images that were not focused correctly were excluded. Cell segmentation was performed using the pixel classification workflow of ilastik, and prediction maps were exported for each time point. The resulting time stacks were aligned, and cell coordinates and mean speed were calculated using the ImageJ plugins StackReg (42) and TrackMate (43). Speeds were weighted by the length of each track. Trajectory maps were calculated and plotted using ggplot2 (44) in R (45) (Supplemental Figure 6E). Results were consistent across 3 independent experiments.

### Transfection with scrambled and HMCN1 siRNA.

Transfection with HMCN1 siRNA (L-013514-00-0010, Horizon Discovery) and nonspecific siRNA (D-001810-10-05, Horizon Discovery) was performed according to the manufacturer’s instructions (DharmaFECT Transfection Reagents, siRNA transfection protocol). In short, cells were seeded in a clear-bottomed, 96-well plate (89626, ibidi) at a density of 20,000 cells/well in DMEM containing 5% FCS. The following day, HMCN1 siRNA, nonspecific siRNA, and DharmaFECT transfection reagent were diluted in serum-free media and incubated for 5 minutes at room temperature. Subsequently, the diluted transfection reagent was mixed with siRNA dilutions and incubated for 20 minutes at room temperature. After this, mixed siRNA/transfection reagent was diluted into 5% FCS medium, and after removing the culture medium, 200 μL transfection media were added to each well. After 24 hours of incubation, the media were changed to a CO_2_-independent medium (18045054, Gibco, Thermo Fisher Scientific; 5% FCS), and migration tracking was performed as described above. For RT-qPCR validation of siRNA knockdown, 60,000 cells were seeded in each well of a 6-well plate, transfected with nonspefific or HMCN1 siRNA as described above, and incubated for 24 hours in culture medium (5% FCS). Subsequently, cDNA was extracted and RT-qPCR was performed as described above.

### ECM and kidney sample preparation for MS analysis.

To obtain ECM, 4 × 10^5^ WT or *ADAMTS12*-KO cells were seeded in 10 cm dishes and cultured overnight. Next, cells were serum starved (DMEM, 0.5% FCS) for 24 hours and then stimulated with 10 ng/mL TGF-β (100-21-10UG, Peprotech) to induce ECM production. After 72 hours, ECM was isolated according to an ECM isolation protocol by Hellewell et al. (46). After removing the cell culture medium and washing cells with PBS, cells were removed by incubation with 3 mL 20 mM ammonium hydroxide at room temperature. After 5 minutes, 20 mL deionized H_2_O was added, before removing the diluted ammonium hydroxide and solubilized cell solution. The remaining ECM layer was then washed 4 times with deionized H_2_O. Complete cell removal was confirmed using a microscope, before harvesting and denaturing the ECM by adding 4% SDS in 100 mM Tris-HCl, pH 8.5. For each replicate, eight 10 cm dishes were pooled. Proteins were precipitated overnight with ice-cold acetone (80% final concentration). The next day, precipitates were spun down for 30 minutes at full speed and washed twice in 80% acetone. Pellets were resuspended in 2% sodium deoxycholate (SDC) in 100 mM Tris-HCL, pH 8.5. For the proteome analysis of kidney material after UUO, kidney pieces from WT and *Adamts12*^—/—^ mice were snap-frozen after PBS perfusion and homogenized and lysed in 2% SDC in 100 mM Tris-HCL, pH 8.5, using the BeatBox (Preomics). Protein concentrations of all samples were normalized using the BCA protein assay kit (Thermo Fisher Scientific), and proteins were reduced with 10 mM tris(2-carboxyethyl)phosphine (TCEP) and alkylated with 40 mM 2-chloroacetamide (CAA). After overnight digestion with LysC and Trypsin (1:100, enzyme/protein, w/w) at 37°C, peptides were cleaned using styrenedivinylbenzene reverse phase sulfonate stage tips (Thermo Fisher Scientific).

### Chromatography and MS.

Samples were loaded onto 50 cm columns packed in-house with C18 1.9 μM ReproSil particles (Dr. Maisch GmbH) using the EASY-nLC 1000 system (Thermo Fisher Scientific) coupled to the mass spectrometer (Exploris 480, Thermo Fisher Scientific). A homemade column oven maintained the column temperature at 60°C. Peptides were eluted with a 120-minute gradient starting at 5% buffer B (80% ACN, 0.1% formic acid) followed by a stepwise increase to 30% over 95 minutes, 60% over 5 minutes, 95% over 10 minutes, and 5% over 10 minutes at a flow rate of 300 nL/min. For the analysis of WT and *ADAMTS12*-KO PDGFRβ^+^ cell proteomes, a data-independent acquisition MS method was used in which 1 full scan (300–1,650 *m/z*, *R* = 120,000 at 200 *m/z*) at a target of 3 × 10^6^ ions was first performed, followed by 48 windows with a resolution of 15,000, in which precursor ions were fragmented with higher-energy collisional dissociation (fixed collision energy 27%) and analyzed with a customized AGC target and maximum injection time in profile mode using positive polarity. For the analysis of WT and *Adamts12^–/–^* kidney proteomes, samples were measured in data-dependent acquisition with a (TopN) MS method, in which 1 full scan (300–1,650 *m/z*, *R* = 60,000 at 200 *m/z*) at a target of 3  × 10^6^ ions was first performed, followed by 15 data-dependent tandem MS (MS/MS) scans with higher-energy collisional dissociation (target 10^5^ ions, maximum injection time at 28 ms, isolation window 1.4 *m/z*, normalized collision energy of 30%, and *R* = 15,000 at 200 *m/z*). Dynamic exclusion of 30 seconds was enabled.

### Cloning of overexpression constructs, virus production, and transduction.

The coding sequence (CDS) for a human ADAMTS12-1xHA-fusion protein was ordered as 2-codon optimized gBlocks (N-terminal part and C-terminal part-1xHA, IDT). The 2 fragments were blunt-end cloned into pSC-B-amp/kan (StrataClone Blunt PCR Cloning Kit; 240207) and subsequently transferred into Stbl3 cells (Invitrogen, Thermo Fisher Scientific, C737303). The N-terminal half of *ADAMTS12* was then transferred from pSC into pMIG (Addgene plasmid 9044, a gift from William Hahn) via Xho-EcoRI digestion. Afterwards, the C-terminal-part including the HA-Tag was introduced via EcoRI digestion. Sequencing was used to verify the error-free insertion of the CDS encoding the fusion protein of *ADAMTS12* coupled to an HA tag. To create a catalytically inactive *huADAMTS12* pMIG vector, the Q5 Site-Directed Mutagenesis Kit (New England Biolabs [NEB], E0554) was used according to the manufacturer’s instructions and with the primer combination 5′-CACAATTGCCcaagcgCTAGGACACAG-3′ and 5′-AAAGCCAGAGGGAGTCCC-3′ to induce H465Q-E466A mutations as previously described (25). Amphotrophic retroviruses were generated by transfection of HEK293T cells using TransIT-293 transfection reagent (Mirus, no. 2700) and the helper plasmid pUMVC (packaging plasmid) and pMD2.G (pseudotyping plasmid). The transduction of PDGFRβ cells was carried out by incubating them for 48 hours with the virus supernatant from transfected HEK293T cells. Successfully transduced target cells were enriched by FACS of GFP^+^ cells.

### IP of HMCN1 and in vitro digestion assays using recombinant ADAMTS12.

Total cell lysates (TCL) from cells grown to 80% confluence were made using lysis buffer (50 mM Tris-HCl, pH 7.5, 150 mM NaCl, 1 mM EDTA, 1% NP-40, 2 mM TCEP, and 10% glycerol) containing Complete EDTA-Free Protease Inhibitors (Roche, 11836170001) and PhosSTOP Phosphatase Inhibitors (Roche, 4906845001). Seven percent of the TCL was saved as a loading control. The residual lysate was used for IP using 50 μL ProteinG Sepharose 4 Fast Flow suspension (Cytiva, 17-0618-01) and 2 μg anti-HA antibody (BioLegend, 901533) for 1 hour at 4°C. Following this, IP mixtures were split in half again, washed 3 times with lysis buffer, and equilibrated by washing 3 times with ADAMTS12 digestion buffer.

For in vitro digestions, the pellets were dissolved in 30 μL ADAMTS12 buffer supplemented with vehicle or 90 ng recombinant ADAMTS12 (cistim) for digestion of IP lysates and 90 or 180 ng recombinant ADAMTS12 (cistim) for digestion of supernatant from RPE cells (ATCC, CRL-2302), and the mixtures were incubated for 12 hours at 37°C. Recombinant ADAMTS12 activity was verified by parallel digestion of 1,000 ng recombinant COMP (R&D Systems, 3134-CPB-050). The denatured samples were separated by SDS-PAGE and transferred onto a nitrocellulose membrane for Western blot analysis. The following primary antibodies were used for detection: anti-ADAMTS12 (Invitrogen, Thermo Fisher Scientific, PA5-68084, 1:1,000), anti-HMCN1 (MilliporeSigma, HPA051677, 1:2,000) or polyclonal rabbit Anti-HMCN1 (raised against a recombinantly expressed and purified HMCN1 fragment containing the first thrombospondin type I domain including the nidogen G2F domain), and anti-COMP (Abcam, ab231977, 1:1,000). The light chain–specific anti–rabbit IgG from Jackson ImmunoResearch (211-031-171; 1:5,000) was used as a secondary antibody.

### Western blot analysis of murine samples.

Murine kidney tissue was lysed using RIPA lysis buffer containing complete protease inhibitor cocktail and phosSTOP phosphatase inhibitor (Roche) for 30 minutes at 4°C. After centrifugation at 10,000*g* for 30 minutes at 4°C, the protein concentration of supernatants was quantified. TCL (30 μg) was separated by SDS-PAGE (the concentration of acrylamid was chosen by the size of the protein to be detected) and transferred to nitrocellulose. The membranes were incubated with specific primary antibodies overnight at 4°C with gentle shaking, followed by HRP-conjugated secondary antibodies (Vector Laboratories, PI-2000-1 and PI-1000-1) for 1 hour at room temperature. Protein bands were visualized using the Pierce ECL Western Blotting – Substrate and ChemiDoc Touch Imaging System (Thermo Fisher Scientific) and quantified with Image Lab Software (Bio-Rad). The primary antibodies used are listed in Supplemental Table 14.

### Statistics and reproducibility.

Data are presented as the mean ± SD. For analysis of multiple groups, a 1- or 2-way ANOVA with Tukey’s post hoc test was applied as indicated. For analysis of 2 groups, an unpaired, 2-tailed *t* test was used. A paired, 2-tailed *t* test was only used for the comparison of *ADAMTS12* expression in PDGFRβ^+^/COL1A1^+^ and PDGFRβ^–^/COL1A1^–^ cells in the human tissue microarray. Correlations were calculated using Pearson’s correlation coefficient. Statistical analyses were performed using GraphPad Prism 9.3.0 (GraphPad Software). A *P* value of less than 0.05 was considered statistically significant. All cell culture experiments, with the exception of bulk RNA-Seq, siRNA, and mass spectrometry, were reproduced in 3 independent experiments.

### Study approval.

All animal experiments were approved by regional authorities (Landesamt für Natur, Umwelt und Verbraucherschutz Nordrhein-Westfalen, Recklinghausen, Germany). All human tissue protocols were approved by the local ethics committee of the University Hospital RWTH Aachen. All patients provided informed consent, and the study was conducted in accordance with the Declaration of Helsinki.

### Code availability.

All custom scripts used in this publication are available in the following GitHub repository: https://github.com/lastprog/ADAMTS12

### Data availability.

Microarray, spatial sequencing, and bulk RNA-Seq datasets are available at zenodo (https://zenodo.org/records/12506371). Values for all data points in graphs are reported in the Supporting Data Values file.

Mass spectrometry proteomics data are available in the ProteomeXchance Consortium (PXD040152) database: https://www.ebi.ac.uk/pride/archive/projects/PXD040152 DEGs are presented in Supplemental Tables 1, 8, and 11–13.

## Author contributions

KH, LK, and RK designed the study, interpreted the data, and wrote the manuscript. RKS, FP, AW, SH, LDL, JH, BDH, and JJ advised on data interpretation and edited the manuscript. The microarray was performed by RK. LK and KH analyzed the microarray, bulk RNA-Seq, and human CKD data with support from HK. Gene-regulatory network analysis was performed by FS and KH, and LK, SM, and GJLS performed UUO surgeries. MI surgeries, echocardiography, and analysis were performed by DS, LK, KH, and QL. LK, BMK, and PB performed Picrosirius red and H&E staining. LK, QL, CSSM, and LZ performed ISH, IHC, and IF stainings under the supervision of KH. Analysis of Visium data was performed by DM, LK, SM, and KH under the supervision of SH. LK induced CRISPR-mediated *ADAMTS12*-KO and catalytically active and inactive *ADAMTS12* overexpression under supervision of SZ and KH. Western blotting were performed by SZ. LK performed live cell imaging experiments with the help of LZ. ECM isolation was performed by KH. Mass spectrometry was performed by MCT and MM. LK performed all other cell culture experiments with QL and LZ. ADAMTS12 chimeras were synthesized by MB and MV. Digestion assays were performed by SL, GS, SZ, and SK. All authors read and approved the final manuscript.

## Supplementary Material

Supplemental data

Unedited blot and gel images

Supplemental table 1

Supplemental table 10

Supplemental table 11

Supplemental table 12

Supplemental table 13

Supplemental table 14

Supplemental table 15

Supplemental table 16

Supplemental table 2

Supplemental table 3

Supplemental table 4

Supplemental table 5

Supplemental table 6

Supplemental table 7

Supplemental table 8

Supplemental table 9

Supplemental video 1

Supplemental video 2

Supporting data values

## Version 1 09/17/2024

Electronic publication
