# Supplementary material for: ADAMTS12 promotes fibrosis by restructuring extracellular matrix to enable activation of injury-responsive fibroblasts
Source: J Clin Invest. 2024 Sep 17;134(18):e170246. doi: 10.1172/JCI170246 (PMC11405035; doi:10.1172/JCI170246)
Supplement: Supplemental data [file jci-134-170246-s066.pdf]

Supplemental information

ADAMTS12 promotes fibrosis by restructuring extracellular matrix to enable activation of injury-responsive fibroblasts

Konrad Hoeft, Lars Koch, Susanne Ziegler, Ling Zhang, Steffen Luetke, Maria C. Tanzer, Debashish Mohanta, David Schumacher, Felix Schreibing, Qingqing Long, Hyojin Kim, Barbara M. Klinkhammer, Carla Schikarski, Sidrah Maryam, Mathijs Baens, Juliane Hermann, Sarah Krieg, Fabian Peisker, Laura De Laporte, Gideon J.L. Schaefer, Sylvia Menzel, Joachim Jankowski, Benjamin D. Humphreys, Adam Wahida, Rebekka K. Schneider, Matthias Versele, Peter Boor, Matthias Mann, Gerhard Sengle, Sikander Hayat, Rafael Kramann

Supplemental methods

Microarray analysis

Microarray gene expression was quantified using the R package affy (v.1.72) (1) aligning probes to the mouse genome *Mouse4302.db* and normalizing gene expression using the Robust-Multichip Average (RMA-) function. After principal component analysis, the R package limma (v.3.44.1) (2) was used to test differential gene expression between UUO and sham conditions using the RunLimma function. In case that two or more microarray probes mapped to the same gene, duplicate genes were removed. PROGENy and DoRothEA transcription factor analyses were performed as described below.

scRNA and snRNA analysis

For analysis of *ADAMTS12* expression we used scRNA-seq datasets of human PDGFRB^{+} cells (Mesenchyme enriched) from 8 human kidneys and CD10^{-} cells (Proximal Tubule depleted) from 15 human kidneys that was recently published by our group (3). In addition we assessed *ADAMTS12* expression in a large human kidney scRNA-seq dataset, which was generated by the "Kidney Precision Medicine Project" (KPMP) (4). Quality control, data integration and annotation are described in the original publications (3, 4). *ADAMTS12*, *ACTA2*, *PDGFRB* and *POSTN* expressions were analyzed using *Seurat* (v4.1.1). For analysis of *Adamts12* expression in mouse kidneys after AKI we used a publicly-available snRNA-seq time course of mouse kidneys after ischemia-reperfusion injury (IRI). The annotated dataset was provided by the original authors (5).

Human kidney tissue microarray

Human tissue Microarrays (hTMA) were generated as described before (3). In short, paraffin-embedded, formalin-fixed kidney specimens from 42 non-tumorous human kidney samples were obtained from the Eschweiler/Aachen biobank. The age of the patients ranged from 30 years to 86 years with a mean of 66.22 years (standard deviation of 12.97 years). One 2 mm core was taken from a randomly selected area from each kidney sample using the TMArrayer (Pathology Devices, Beecher Instruments). Each core was arrayed into a recipient block, from which 2 µm thick sections were cut. All patients gave informed consent in accordance with the Declaration of Helsinki.

Hematoxylin-Eosin and Picro-Sirius Red staining and quantification

Hematoxylin and eosin staining was performed on FFPE sections from different organs from male and female *Adamts12*$^{-/-}$ animals. Picro-Sirius Red staining was performed on human tissue microarrays and mouse hearts using the Morphisto Sirius Red Staining Kit (13425, Morphisto) following the manufacturer's instructions. For human tissue microarray stainings 7 images were acquired from the renal cortex in a randomized fashion, using the 40x objective of a brightfield microscope (BZ-9000, Keyence, IHC) and fibrosis was automatically quantified using ImageJ (6, 7). For MI experiments whole slides were scanned using the Aperio Slide Scanner (Leica Biosystems). Fibrosis was automatically quantified with the Aperio eSlide Manager. MI scar size was quantified by manual annotation of a trained pathologist.

Gene regulatory network inference

For inference of gene regulatory networks (GRNs) we used a publicly available kidney data set, which was generated by the "Kidney Precision Medicine Project" (KPMP) using the SLAM-seq2 protocol (4). Thus, the data set provided matched snRNA-seq and snATAC-seq information for each cell, which improves inference of GRNs. The raw count matrices were downloaded from the Gene Expression Omnibus database (GSE183273). Using the cell type annotations provided by KPMP, we subsetted count matrices of fibroblast populations. Subsequently, the snRNA-seq data was processed using *Seurat* (version 5.0.1) and the snATAC-seq data was processed using *Signac* (version 1.12.0), and a combined object was created. The combined Seurat object was used as input for GRN inference using *scMEGA* (version 1.0.2) (8). Trajectory inference is a critical step during GRN construction using

scMEGA and is based on supervised trajectory inference implemented in *ArchR (9)*, which requires the user to define an origin and endpoint for the inferred trajectory. As the quality of GRNs is highly influenced by the underlying trajectory, we aimed to define cell types and thus origin and end points of the trajectory in a cluster-independent and biologically meaningful manner. Here, we calculated a myofibroblast signature for each cell using the *AddModuleScore* function implemented in *Seurat* in combination with two well-defined myofibroblast gene sets, (1) the *TRAVAGLINI_LUNG_MYOFIBROBLAST_CELL* gene set (10) and (2) the *NABA_CORE_MATRISOME* gene set (11). The resulting score values were then used as the X- and Y-coordinates for a myofibroblast-specific embedding of all cells in the data set. Myofibroblasts were defined as cells with a positive core matrisome score and a myofibroblast signature of more than 50% of the maximum score. Subsequently fibroblast-to-myofibroblast trajectories were inferred using *ArchR* (version 1.0.2) and the quality of the inferred trajectories was confirmed by investigation of the expression of known myofibroblast marker genes along pseudotime. Finally, transcription factor activity was inferred using *chromVAR* (version 1.22.1) (12) and GRNs were constructed following the standard workflow for scMEGA. For the selection of transcription factors, a correlation cutoff of 0.4 was used. For the selection of genes, a variation cutoff of 0.9 and a cutoff of 0.05 for the adjusted p-value were selected. For visualization of the sub-GRN for BACH1 and JUNB, the edges were filtered for transcription factor-gene pairs with a correlation above 0.8.

CrossTalkeR ligand receptor analysis

To determine cell-cell communication, Ligand Receptor (LR) analysis was performed using the CrossTalkeR (version 1.4.0) package in R. CrossTalkeR internally uses LIANA for each condition (i.e. case and control) listed in the dataset. LIANA version 0.1.13 was used along with Omnipath database (version 3.10.1) of known ligand receptors to generate LR pairs. LIANA uses different methods like Natmi, Connectome, Logfc, sca, Cellphonedb from which LR pairs were selected on the basis of p-values (< 0.01), for each case and condition. The final cell-cell interaction report on WT vs *Adamts12*$^{-/-}$ basis was generated using the predefined CrossTalkeR function generate_report().

Spatial gene expression - data analysis workflow Spatial transcriptomics pre-processing

Spaceranger (version 1.3.0) was used to align sequencing output. First, the *mkfastq* function was used to convert the bcl files to fastq files. These fastq files along with the tissue images were then used as inputs to the *count* function to align the fastq files to a reference genome (version mm10-2020), and perform tissue and automated fiducial alignment. Quality control (QC) was then performed on the individual count matrices. Specifically, spots with mitochondrial read counts > 60%, and total read counts < 400, were filtered out. Furthermore, genes that were detected in < 3 spots were also filtered out. This resulted in a total of 1511, 1290, 1278, and 1205 number of spots for each slide. The two Knockout samples and two wild-type samples were integrated using *Harmony* (13) and sample ID as the batch variable. Here, the top 2000 highly variable genes were identified using the *Seurat_v3* method, and PCs were calculated using the *RunPCA* method in Seurat. For Harmony (13) the top 20 PCs were used. The integrated data was clustered using the Leiden algorithm in Scanpy (14). Clustering with a resolution of 0.5 was used for all subsequent analyses. Here, 6 clusters/zones were found and marker genes were calculated using the *FindAllMarkers* function (version 4.3.0) using the MAST algorithm. The clusters/zones were then manually annotated.

Cell-type deconvolution

Tangram (15) was used for cell-deconvolution of each spot. Here, our previously published human heart cell-atlas with 191795 cells was used as the reference dataset (16). Cell-type deconvolution was able to identify the major 7 cell-types (Fibroblast, Endothelial,

Cardiomyocytes, Myeloid, Pericytes, Neuronal, vSMCs) that were observed in the reference dataset.

Characterization of infarction zones

To quantify spatial *Adamts12* expression, we summed the normalized expression per zone and cell type per spot. For assessing changes in cell type composition, we scaled the cell type probability score per spot to 100% and plotted its mean and log2-fold change by cell type and zone. P-values were determined using an unpaired t-test. Differentially expressed genes within the ischemic zone were imputed using the MAST algorithm of Seurats FindAllMarkers function. Gene set enrichment analysis was performed based on significantly upregulated genes (adjusted p-value<0.05) using *gprofiler2* (version 0.2.1) (17). Reactome pathways with a term size greater than 10 were selected (18). Biological processing GO Terms were recognized at a term size lower than 2000 genes to exclude nonspecific terms. PROGENy (version 1.20.0) pathway and DoRothEA transcription factor analysis was carried out as described below.

Fibroblast subset estimation and mapping to spatial data

To estimate fibroblast subsets in our spatial transcriptomic data, we integrated the spatial gene expression dataset with our previously published scRNA-seq dataset of murine fibroblasts (19) in heart failure using Seurat (v4.1.1). After normalization and variance stabilization using *SCTransform,* we integrated and estimated fibroblast subset scores using the *FindTransferAnchors* and *TransferData* functions from Seurat. Fibroblast subset scores were corrected for the overall fibroblast probability per spot by multiplying the initially estimated fibroblast Tangram prediction score with the fibroblast subset score for each spot. Significance levels were determined using an unpaired t-test, and p-values were corrected for multiple hypothesis correction.

Generation of a human PDGFRB$^+$ cell line

A human PDGFRB$^+$ cell line was generated as described before (3). Briefly, PDGFRB$^+$ cells were isolated from the renal cortex of a 71-year-old male individual using magnetic-activated cell sorting (MACS) and immortalized using SV40LT and HTERT.

Cell maintenance

Cells were cultured in DMEM media (31885-023, Gibco), substituted with 5% FCS, 1% Penicillin Streptomycin (15140122, Gibco) and 0.2% MycoZap (VZA2022, Lonza). Cells were split at 70–80% confluency using 0.5 mM Trypsin (25300054, Thermo Scientific) and cell aggregates were reseeded by diluting 1:2.

Bulk RNA Analysis

Bulk RNA data was preprocessed as recommended by the nf-core nextflow pipeline (version 21.04.1) using nf-core/rnaseq (version 3.1) (20), star (version 2.7.9a) for read alignment (21), salmon (version 1.5.0) for read quantification (22), trimgalore (version 0.6.6) for read trimming, and gencode (version 38) for gene annotation (23). For differential gene expression analysis, lowly expressed genes, “rRNA” ,”tRNA” and “mtRNA” were filtered out. Subsequently, differentially expressed genes were calculated using DESeq2 (version 1.320) (24). Of note, we did not observe a relevant compensatory upregulation of ADAMTS12 homologues, such as ADAMTS7 (Supplemental Figures 7J, 9G, Supplemental Table 12).

Pathway RespOnsive GENes (PROGENy) for activity inference

For gene expression data obtained from spatial transcriptomics, PROGENy pathway activity was inferred based on genes sorted by their average log2-fold change, using the

murine version of PROGENy (version 1.16.0). PROGENy pathway analysis of Microarray and bulk RNA sequencing data were performed as described previously (25). In summary, pathway activity scores were imputed based on the results of differential expression analysis in bulk or Microarray data. A null-distribution was subsequently generated by repeated permutation (10,000x) of t-values and original pathway scores were scaled to their respective null distribution to generate a normalized pathway enrichment score (NES). For scRNA-seq inference of PROGENy pathway activity in the human KPMP dataset we subsetted interstitial cells and performed analysis as recommended by a benchmark study using the top 500 most responsive genes (26).

DoRothEA Transcription Factor inference

Transcription factor activity was inferred based on t-values for the microarray and bulk RNA sequencing data and for spatial sequencing analysis based on the average log2-fold change obtained from differential expression analysis, using DoRothEA (version 1.6.0) and a collection of transcription factor targets together with VIPER (version 1.28.0) as previously described (25). For imputing transcription factor activity, Dorothea regulons with confidence levels A, B, and C were used.

Gene set enrichment analysis (GO-Terms)

GO-Terms were calculated based on the top 500 DEG sorted by t-value using gprofiler2 (version 0.2.1) (17). Biological processing GO Terms were recognized at a term size lower than 2000 genes to exclude nonspecific terms.

Scoring ActvsKO and ActvsInact signatures in Fibroblast scRNA-Seq data

To score ActvsKO and ActvsInact activation signatures in a fibroblast scRNA-seq dataset (19) we used the function *AddModuleScore* (Seurat v4.0) using significantly upregulated genes (adj. p-value <0.5) derived from differential gene expression analysis of either active ADAMTS12 expressing cells vs *ADAMTS12*-KO cells, or active vs inactive ADAMTS12 expressing cells as described above. For statistical comparison of scores significance levels were calculated using a one-way ANOVA with Tukey's post-hoc test.

Broad-spectrum metalloproteinase inhibition using Batimastat

60.000 cells per well were seeded in 6 well plates and incubated for 24 hours in 5% FCS media. Batimastat (S7155, Selleck Chemicals) was diluted in DMSO according to the manufacturer's instructions and subsequently added to the media for a final concentration of 20 nM. After 24 hours cells were harvested and RT-qPCR was performed as described above.

Quantification and statistical analysis of mass spectrometry data

MS raw files were processed by the Spectronaut software version 15 (Biognosys (27)) using directDIA with default settings. The human (42,351 entries, additional 57,749 entries, 2021) uniport FASTA database as a forward database was used. N-terminal acetylation and methionine oxidations were set as variable modifications, and cysteine carbamidomethylation was set as fixed modification. Enzyme specificity was set as C-terminal to Arginine and Lysine as expected, using Trypsin and LysC as proteases and a maximum of two missed cleavages. All bioinformatic analyses were done using Perseus software (version 1.6.2.2) (28). Quantified proteins were filtered for at least 100% of valid values among three biological replicates in at least one condition. Missing values were imputed, and significantly up- or down-regulated proteins were determined by the Student's t-test (two-sided) (FDR = 0.05).

For the experiment measured in DDA mode, MS raw files were processed by the MaxQuant (29) version 2.1.4.0 and fragments lists were searched against the human UniProt

FASTA database (March 2021) with cysteine carbamidomethylation as a fixed modification and N-terminal acetylation and methionine oxidations as variable modifications. The false-discovery rate (FDR) was set to less than 1% at the peptide and protein levels and specified a minimum length of seven amino acids for peptides. Enzyme specificity was set as semispecific including cleavage C-terminal to arginine and lysine as expected using trypsin and lysC as proteases and a maximum of two missed cleavages.

Gene set enrichment analysis of mass spectrometry data

GO-Terms were calculated based on significantly down-regulated proteins (WT vs *Adamts12*$^{-/-}$, q.value < 0.05, log2FC<0) using gprofiler2 (version 0.2.1) (17). Biological processing GO Terms were recognized at a term size lower than 2000 genes to exclude nonspecific terms. For analysis of DB matrisome (11), fgsea (version 1.20.0) gene set enrichment analysis was performed with default parameters using significantly up- and downregulated proteins (WT vs *Adamts12*$^{-/-}$, q.value < 0.05) as input.

Immunoprecipitation of ADAMTS12, in vitro digestion assay to examine COMP degradation and Western Blotting

Total cell lysates from cells grown to 80% confluency were made using lysis buffer (50 mM Tris-HCl pH 7.5, 150 mM NaCl, 1 mM EDTA, 1% NP-40, 2 mM TCEP and 10% glycerol) containing Complete EDTA-free Protease Inhibitors (Roche #11836170001) and PhosSTOP Phosphatase Inhibitors (ROCHE #4906845001). 7% of TCL were saved as loading control. The residual lysate was used for immunoprecipitation using 50 µl of ProteinG Sepharose 4 Fast Flow suspension (Cytiva #17-0618-01) and 2 µg anti-HA antibody (BioLegend #901533) for 1 hour at 4°C. The resulting pellets were washed three times with lysis buffer and equilibrated for COMP-digestion assay by additional washing with ADAMTS12 digestion buffer (50 mM Tris–HCl pH 7.5, 100 mM NaCl, 5 mM $CaCl_2$, 2 mM $ZnCl_2$, Brij-35 0.05%). Subsequently, the pellets were resuspended in 30 µl ADAMTS12 digestion buffer containing 1 µg recombinant human COMP (R&D #3134-CPB-050) and incubated at 37°C for 12 hours. After COMP digestion for 12 hours, 4x Laemmli sample buffer (BIO-RAD #1610747) was added and the samples were cooked at 95°C for five minutes. Loading controls (see above) and digested samples were resuspended in 8% SDS-Page. For Western Blotting the following antibodies were used: Anti-HA (abcam, #ab9110, 1:4000, for detection of HA-tagged ADAMTS12), Anti-ß-Tubulin (Sigma-Aldrich #T8328, 1:1000, used as loading control), Anti-GFP (Abcam, #ab6556, 1:5000, control for integration of pMIG vector which co-expresses eGFP), Anti-COMP (abcam, #ab231977, 1:1000).

Synthesis of ADAMTS12

A rat/human *ADAMTS12* chimera (rat AA 1-244/human AA 241-543) with a C-terminal 3xFLAG Tag was cloned into the mammalian pcDNA3.4 expression vector (Genscript). Expi293 cells (Life Technologies, A14635) at a density of 2.5 x 10^6 cells/mL were transfected with 1 mg/L of vector plasmid DNA using Expifectamine transfection reagent (Life Technologies, A14525). 72 hours post transfection, the supernatant of the Expi293 cell culture was collected via centrifugation at 4000 rpm for 10 min, neutralized with 50mM Tris pH8.0, 5 mM $CaCl_2$, 10 µM $ZnCl_2$ and centrifuged again at 4000 rpm for 10 min. The supernatant was incubated overnight at 4°C with anti-FLAG M2 affinity gel (Sigma-Aldrich A2220) equilibrated with buffer A (50 mM Tris pH 8.0, 300 mM NaCl, 10% glycerol, 5 mM $CaCl_2$, 10 µM $ZnCl_2$). The gel was collected and washed with 10 bed volumes of buffer A and bound proteins were eluted with buffer A supplemented with 150 ng/µl FLAG peptide (Sigma-Aldrich F4799). Fractions containing ADAMTS12 protein were identified via SDS PAGE, concentrated and stored at -80°C.

References

1. Gautier L, et al. affy--analysis of Affymetrix GeneChip data at the probe level. *Bioinformatics*. 2004;20(3):307–315.
2. Ritchie ME, et al. limma powers differential expression analyses for RNA-sequencing and microarray studies. *Nucleic Acids Res*. 2015;43(7):e47.
3. Kuppe C, et al. Decoding myofibroblast origins in human kidney fibrosis. *Nature*. 2021;589(7841):281–286.
4. Lake BB, et al. An atlas of healthy and injured cell states and niches in the human kidney. *Nature*. 2023;619(7970):585–594.
5. Kirita Y, et al. Cell profiling of mouse acute kidney injury reveals conserved cellular responses to injury. *Proc Natl Acad Sci U S A*. 2020;117(27):15874–15883.
6. Fabian SL, et al. Hedgehog-Gli pathway activation during kidney fibrosis. *Am J Pathol*. 2012;180(4):1441–1453.
7. Schneider CA, Rasband WS, Eliceiri KW. NIH Image to ImageJ: 25 years of image analysis. *Nat Methods*. 2012;9(7):671–675.
8. Li Z, et al. scMEGA: single-cell multi-omic enhancer-based gene regulatory network inference. *Bioinform Adv*. 2023;3(1):vbad003.
9. Granja JM, et al. ArchR is a scalable software package for integrative single-cell chromatin accessibility analysis. *Nat Genet*. 2021;53(3):403–411.
10. Travaglini KJ, et al. A molecular cell atlas of the human lung from single-cell RNA sequencing. *Nature*. 2020;587(7835):619–625.
11. Shao X, et al. MatrisomeDB: the ECM-protein knowledge database. *Nucleic Acids Res*. 2020;48(D1):D1136–D1144.
12. Schep AN, et al. chromVAR: inferring transcription-factor-associated accessibility from single-cell epigenomic data. *Nat Methods*. 2017;14(10):975–978.
13. Korsunsky I, et al. Fast, sensitive and accurate integration of single-cell data with Harmony. *Nat Methods*. 2019;16(12):1289–1296.
14. Wolf FA, Angerer P, Theis FJ. SCANPY: large-scale single-cell gene expression data analysis. *Genome Biol*. 2018;19(1):1–5.
15. Biancalani T, et al. Deep learning and alignment of spatially resolved single-cell transcriptomes with Tangram. *Nat Methods*. 2021;18(11):1352–1362.
16. Kuppe C, et al. Spatial multi-omic map of human myocardial infarction. *bioRxiv*. 2020;2020.12.08.411686.
17. Kolberg L, et al. gprofiler2 -- an R package for gene list functional enrichment analysis and namespace conversion toolset g:Profiler [preprint]. *F1000Research*. 2020;9:709.
18. Gillespie M, et al. The reactome pathway knowledgebase 2022. *Nucleic Acids Res*. 2022;50(D1):D687–D692.
19. Peisker F, et al. Mapping the cardiac vascular niche in heart failure. *Nat Commun*. 2022;13(1):3027.
20. Ewels P, et al. *The nf-core framework for community-curated bioinformatics pipelines*. 2022.
21. Dobin A, et al. STAR: ultrafast universal RNA-seq aligner. *Bioinformatics*. 2013;29(1):15 21.
22. Patro R, et al. Salmon provides fast and bias-aware quantification of transcript expression. *Nat Methods*. 2017;14(4):417–419.
23. Frankish A, et al. GENCODE reference annotation for the human and mouse genomes. *Nucleic Acids Res*. 2019;47(D1):D766–D773.
24. Love MI, Huber W, Anders S. Moderated estimation of fold change and dispersion for RNA-seq data with DESeq2. *Genome Biol*. 2014;15(12):550.

25. Gleitz HFE, et al. Increased CXCL4 expression in hematopoietic cells links inflammation and progression of bone marrow fibrosis in MPN. *Blood*. 2020;136(18):2051–2064.
26. Holland CH, et al. Robustness and applicability of transcription factor and pathway analysis tools on single-cell RNA-seq data. *Genome Biol*. 2020;21(1):36.
27. Bruderer R, et al. Extending the limits of quantitative proteome profiling with data-independent acquisition and application to acetaminophen-treated three-dimensional liver microtissues. *Mol Cell Proteomics*. 2015;14(5):1400–1410.
28. Tyanova S, et al. The Perseus computational platform for comprehensive analysis of (prote)omics data. *Nat Methods*. 2016;13(9):731–740.
29. Cox J, Mann M. MaxQuant enables high peptide identification rates, individualized p.p.b.-range mass accuracies and proteome-wide protein quantification. *Nat Biotechnol*. 2008;26(12):1367–1372.

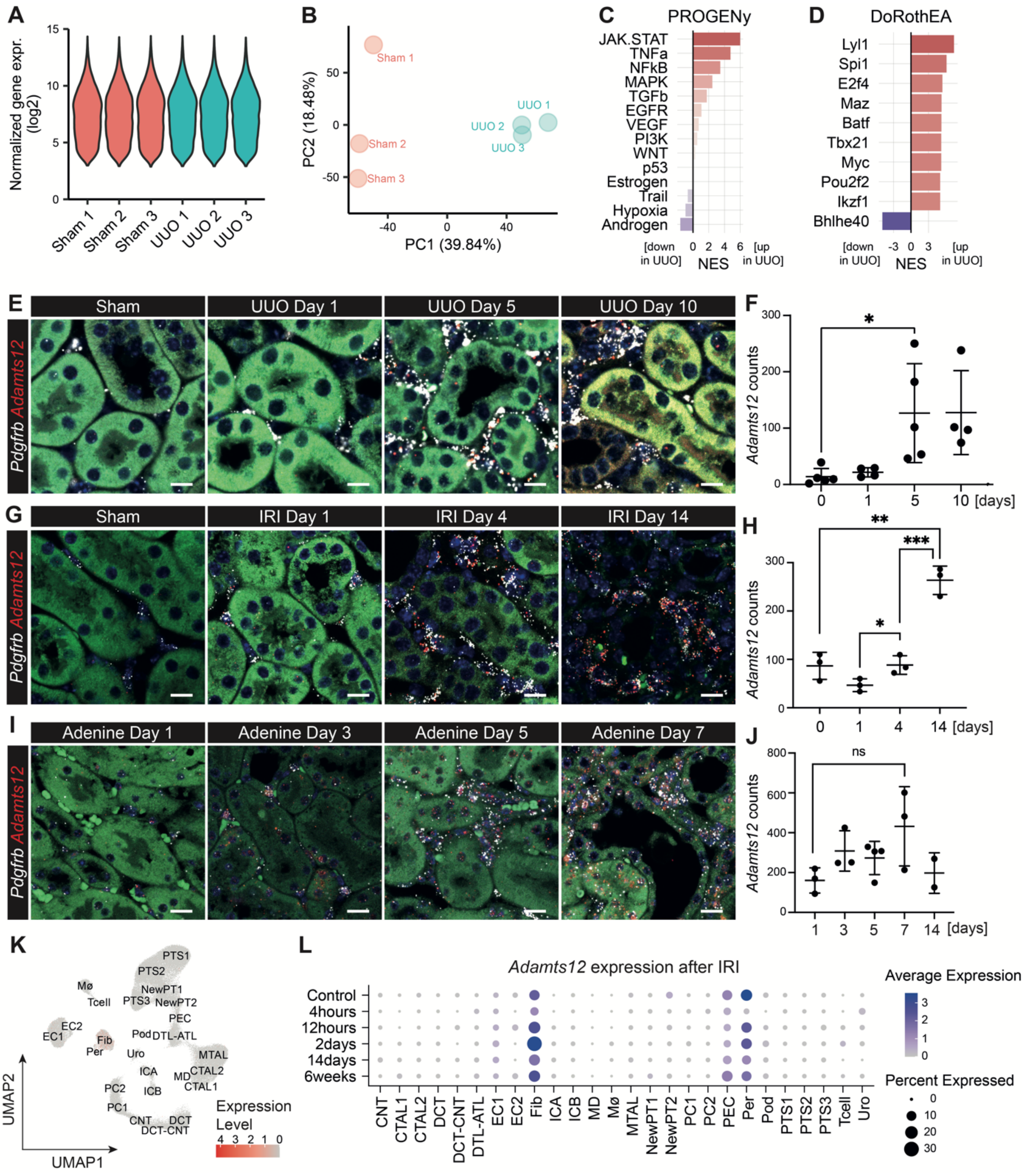

Supplemental Figure 1: *Adamts12* is specifically upregulated in fibroblasts after injury. (A) Normalized logarithmic intensities stratified by sample (n=3 per group). **(B)** Principal component analysis of kidney Gli1^{+} cells after sham or UUO surgery. **(C)** PROGENy pathway analysis of kidney Gli1^{+} cells after sham or UUO surgery. **(D)** Transcription factor (TF) activity prediction based on TF regulons using DoRothEA. **(E, G, I)** ISH for *Pdgfrb* and *Adamts12* in murine kidneys at different time points after unilateral ureter obstruction (UUO), ischemia reperfusion injury (IRI) or adenine nephropathy. Scale bar: 10µm. **(F, H, J)** Quantification of *Adamts12* ISH expression in kidneys at different time points after UUO, IRI or adenine nephropathy. (n=2-5 per group, one-way ANOVA with Tukey's post hoc) **(K)** Featureplot of *ADAMTS12* gene expression in a previously published snRNA-seq dataset of kidney IRI (5). Labels refer to cell-types (see Supplemental table 2). **(L)** Dotplot showing *Adamts12* expression at different timepoints after IRI.

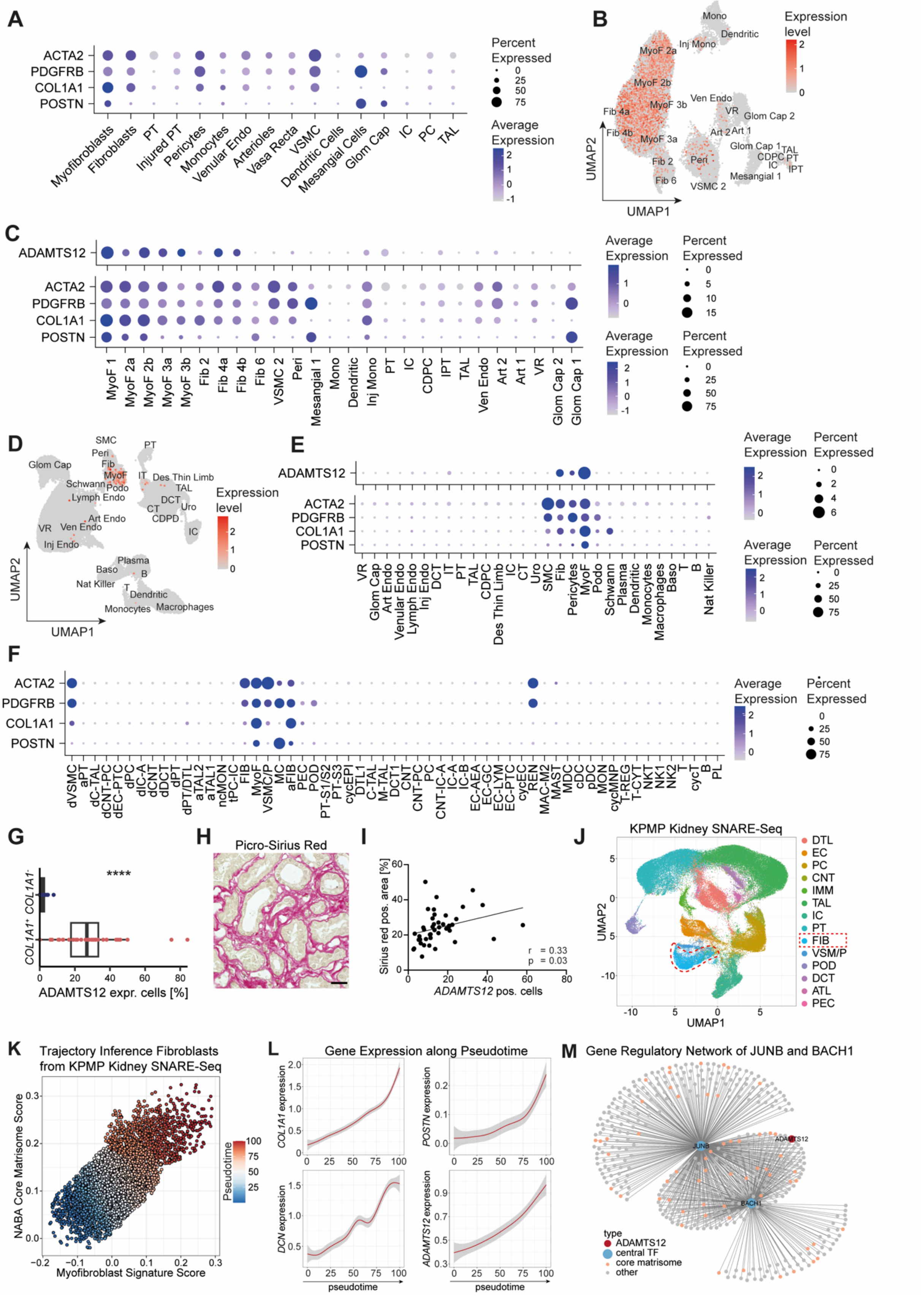

Supplemental Figure 2: *ADAMTS12* expression in human kidney disease.
Dotplot of *ACTA2*, *PDGFRB*, *COL1A1 and POSTN* gene expression in PDGFRB positive cells isolated from human kidney nephrectomies. Abbreviations: PT: Proximal tubule, Injured PT: Injured proximal tubule, Venular Endo: Venular endothelium, VSMC: Vascular smooth muscle

cells, Glom Cap: Glomerular Capillaries, IC: Intercalated cells, PC: Principal cells, TAL: Thick ascending loop of Henle. **(B)** Featureplot of *ADAMTS12* gene expression in human kidney PDGFRB cells shown also in (A) at the highest annotation level. Labels refer to cell types (see Supplemental table 4). **(C)** Dotplot of *ADAMTS12, ACTA2*, *PDGFRB*, *COL1A1 and POSTN* gene expression in PDGFRB cells shown in (B) at the highest annotation level. **(D)** Featureplot of *ADAMTS12* gene expression in a published scRNA-seq dataset of CD10 negative cells (proximal tubule depleted) isolated from human kidney nephrectomies. Labels refer to cell types (see Supplemental Table 4). **(E)** Dotplot of *ADAMTS12*, *ACTA2*, *PDGFRB*, *COL1A1* and *POSTN* gene expression in a dataset of CD10 negative cells shown in (D). **(F)** Dotplot of *ACTA2*, *PDGFRB*, *COL1A1* and *POSTN* gene expression in a previously published KPMP scRNA-seq dataset (see Supplemental Table 5 for cell type abbreviations). **(G)** Quantification of the % of *ADAMTS12*$^+$ cells in *COL1A1*$^+$ or *COL1A1*$^-$ cells (n=43, p<0.0001, two tailed paired t-test). **(H)** Representative image of a picrosirius red staining of human kidneys. Scale bar: 25 µm. **(I)** Pearson correlation of the number of *ADAMTS12*$^+$ cells with ECM as determined by spectral thresholding analysis of picrosirius red stainings shown in H (n=43). **(J)** UMAP embedding of human kidney cells from a SNARE-Seq kidney dataset of the KPMP Project. Fibroblast (circled in red) were subsetted for trajectory inference analysis. **(K)** *ArchR t*rajectory of subsetted fibroblasts from a SNARE-Seq KPMP kidney dataset in a myofibroblast specific embedding based on core matrisome and myofibroblast gene expression. Cells are labeled according to the assigned *ArchR* pseudotime. **(L)** *COL1A1*, *POSTN*, *DCN* and *ADAMTS12* expression along pseudotime. **(M)** Subsetted gene regulatory network for *JUNB* and *BACH1*. Core matrisome genes (ECM collagens, glycoproteins, proteoglycans) are labeled in orange.

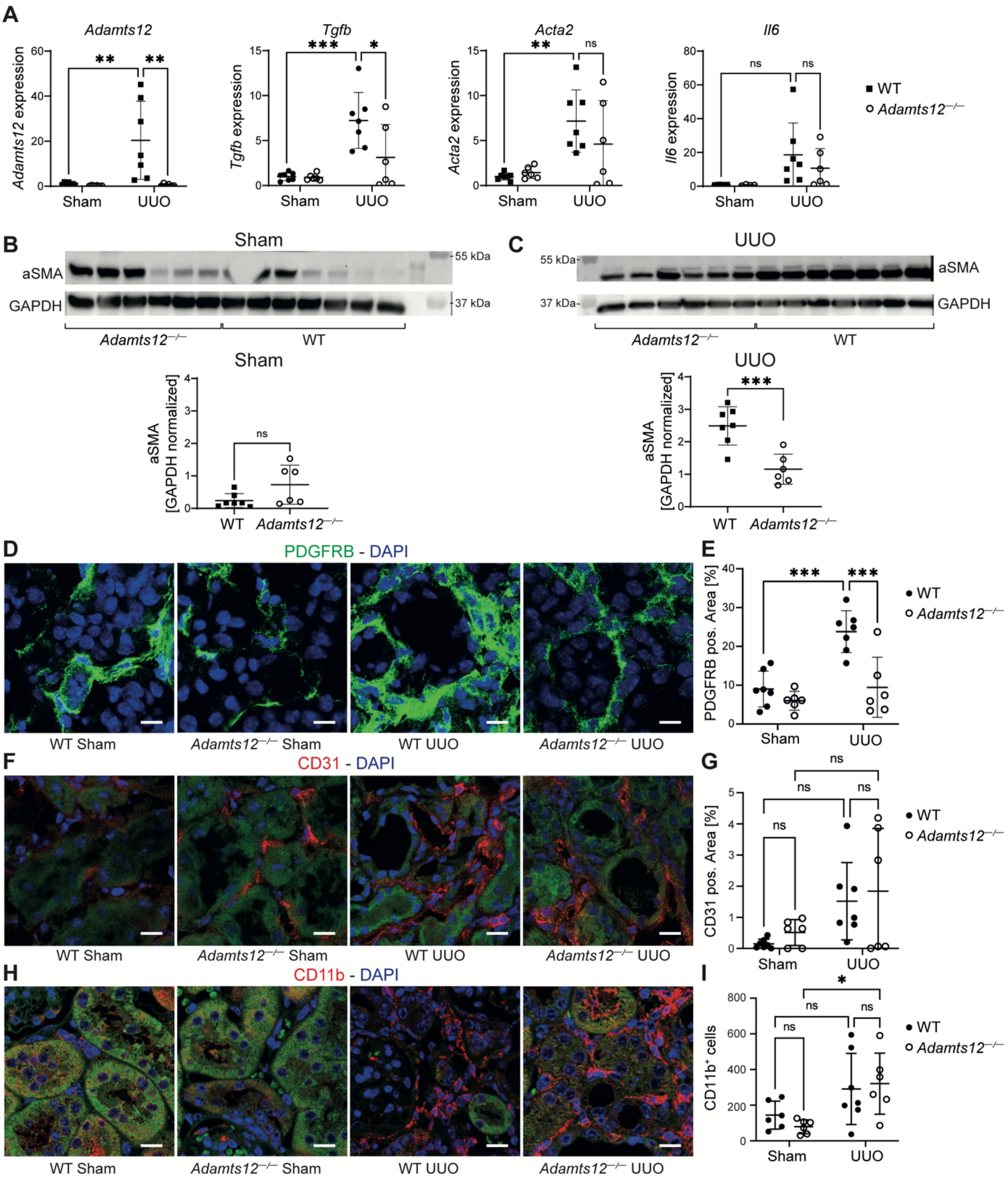

Supplemental Figure 3: Genetic loss of *Adamts12* ameliorates organ fibrosis.

(A) RT- qPCR for *Adamts12* ($p_{WT\ UUO\ vs.\ Adamts12-/-\ UUO}$= 0.0036), *Tgfb* ($p_{WT\ UUO\ vs.\ Adamts12-/-\ UUO}$= 0.0270), *Acta2* ($p_{WT\ UUO\ vs.\ Adamts12-/-\ UUO}$= 0.58) and *IL6* ($p_{WT\ UUO\ vs.\ Adamts12-/-\ UUO}$= 0.69) in kidney tissue of WT and *Adamts12*$^{-/-}$ mice after UUO surgery. (WT n=7, *Adamts12*$^{-/-}$ n=6) **(B)** aSMA Western Blot and quantification of kidneys from WT and *Adamts12*$^{-/-}$ mice after sham surgery. GAPDH Western Blot was used as a loading control. **(C)** aSMA Western Blot and quantification of kidneys from WT and *Adamts12*$^{-/-}$ mice after UUO surgery. GAPDH Western Blot was used as a loading control. **(D, F, H)** Representative images of PDGFRB, CD31 and CD11b immunofluorescence stainings. Scale bars: 25µm. **(E, G)** Quantification of PDGFRB and CD31 positive area based on immunofluorescence stainings shown in D and F (PDGFRb: $p_{WT\ UUO\ vs.\ Adamts12-/-\ UUO}$=0.0004, CD31: $p_{WT\ UUO\ vs.\ Adamts12-/-\ UUO}$=0.96). **(I)** Quantification of CD11b positive cells based on immunofluorescence stainings shown in H

($p_{WT\ UUO\ vs.\ \mathit{Adamts12}—/—\ UUO}$=0.98). For **(B)** and **(C)** an unpaired t-test was performed. All other comparisons were performed by two-way ANOVA with Tukey's post hoc.

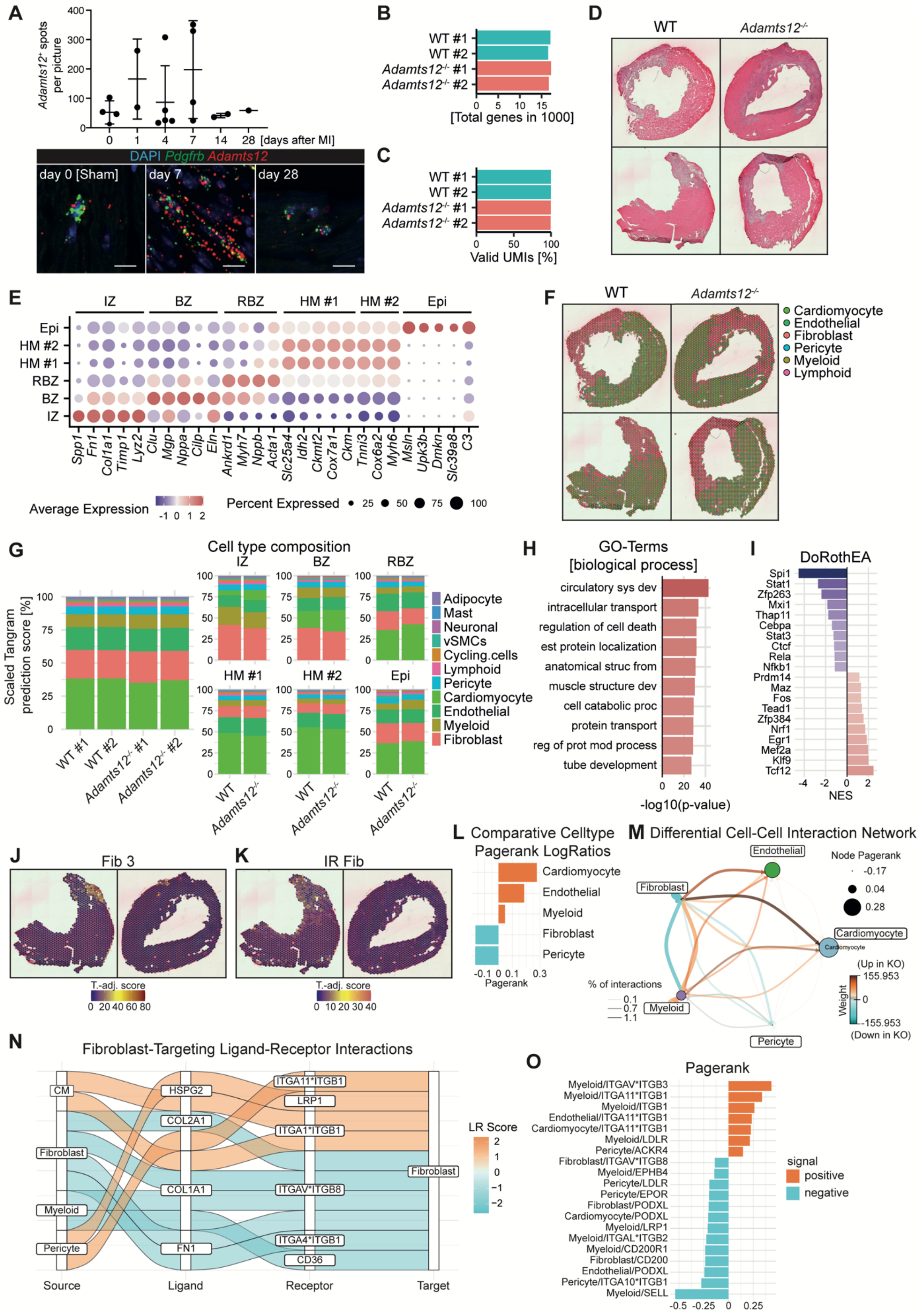

Supplemental Figure 4: Visium spatial transcriptomics of WT and *Adamts12*$^{-/-}$ mice after myocardial infarction.

(A) Automated Quantification of ISH time course for *Adamts12* and *Pdgfrb* in murine hearts 0 (sham), 1, 4, 7, 14 and 28 days after MI. Representative pictures of day 0 (sham), day 7 and

day 28 are shown. Scale bars: 10µm (n=1-5 per group). **(B)** Total detected genes for each spatial transcriptomic sample (n=2 per group). **(C)** Percentage of valid UMIs for each spatial transcriptomic sample. **(D)** HE staining of WT and *Adamts12*$^{-/-}$ hearts selected for spatial transcriptomics. **(E)** Top 5 marker genes for each spatial zone. **(F)** Featureplot of cell type assignment. Spots show the cell type with the highest Tangram Prediction score. **(G)** Scaled Tangram cell type prediction scores stratified by sample and zone. **(H)** Top enriched Biological Process GOs in IZ of *Adamts12*$^{-/-}$ mice based on DEG shown in Figure 3G. Abbreviations: circulatory sys dev: circulatory system development, est protein localization: establishment of protein localization, anatomical struc from: anatomical structure formation involved in morphogenesis, muscle structure dev: muscle structure development, cell catabolic proc: cellular catabolic process, reg of prot mod process: regulation of protein modification process. **(I)** DoRothEA transcription factor activity in IZ based on DEG shown in Figure 3G. **(J)** Spatial Featureplot of Fibroblast 3 Tangram-adjusted prediction score in WT sample #2 and *Adamts12*$^{-/-}$ sample #2. T.-adj. score: Tangram-adjusted prediction score. **(K)** Spatial Featureplot of IR Fibroblast Tangram-adjusted prediction score in WT sample #2 and *Adamts12*$^{-/-}$ sample #2. **(L)** Differential (WT vs *Adamts12*$^{-/-}$) CrossTalkeR pagerank scores (*importance score*) of cell types in spatial transcriptomic sequencing of murine myocardial infarction. Cell types are assigned to spots based on tangram prediction scores. **(M)** Circular plot of differential cell-cell interactions (WT vs *Adamts12*$^{-/-}$) as predicted by CrossTalkeR analysis in spatial transcriptomic sequencing of murine myocardial infarction. Cell types are assigned to spots based on tangram prediction scores. **(N)** CrossTalkeR Sankey plot of differentially expressed ligand receptor interactions (WT vs *Adamts12*$^{-/-}$) in spatial myocardial infarction data, where fibroblasts are the receptor. **(O)** Differential (WT vs *Adamts12*$^{-/-}$) ligand and receptor CrossTalkeR pagerank scores.

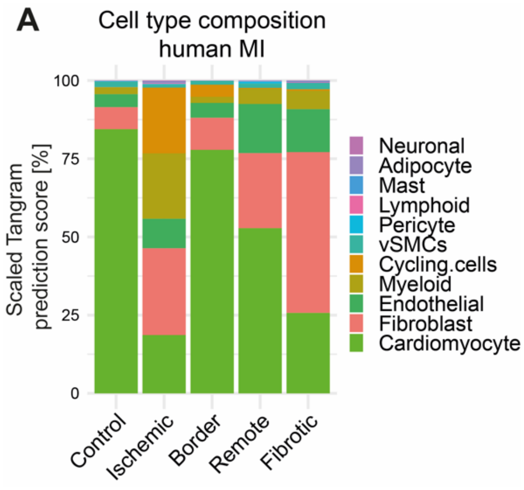

Supplemental Figure 5: *ADAMTS12* expression in spatial multi-omic map of human myocardial infarction.

(A) Cell type composition as determined by scaled Tangram cell type prediction scores.

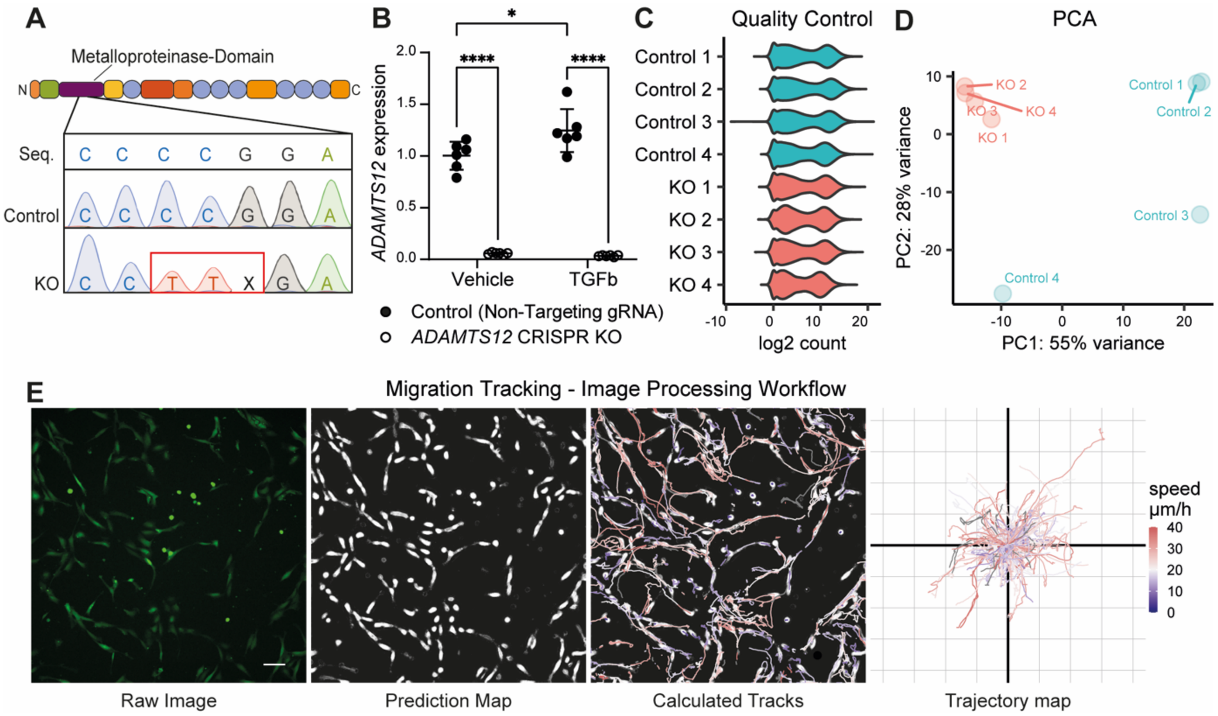

Supplemental Figure 6: CRISPR-Cas9 KO of *ADAMTS12* in human PDGFRB^{+} cells.
(A) Sequencing of the PCR-amplified *ADAMTS12* CRISPR target site in human PDGFRB^{+} kidney cells transduced with a non-targeting guide RNA (control) or an *ADAMTS12* CRISPR-Cas9 vector. Two point mutations and a base deletion were identified in *ADAMTS12*-KO cells. Seq: WT *ADAMTS12* sequence. **(B)** RT-qPCR for *ADAMTS12* (n=6 per group, $p_{\text{NTG TGFb vs. } ADAMTS12\ KO\ \text{TGFb}}<0.0001$, two-way ANOVA with Tukey's post hoc) in control and *ADAMTS12*-KO PDGFRB^{+} fibroblasts after treatment with TGFb or Vehicle. **(C)** Logarithmized gene counts of individual control and *ADAMTS12*-KO PDGFRB^{+} samples (n=4 per group). **(D)** Principal Component Analysis (PCA) of control and *ADAMTS12* KO PDGFRB^{+} samples. **(E)** Image processing workflow for migration tracking: Cell autofluorescence was captured every 10 min for 18-24 hours. Prediction maps were created, and cell tracks calculated. Cell tracks were overlaid starting from the origin. Scale bar: 100 µm.

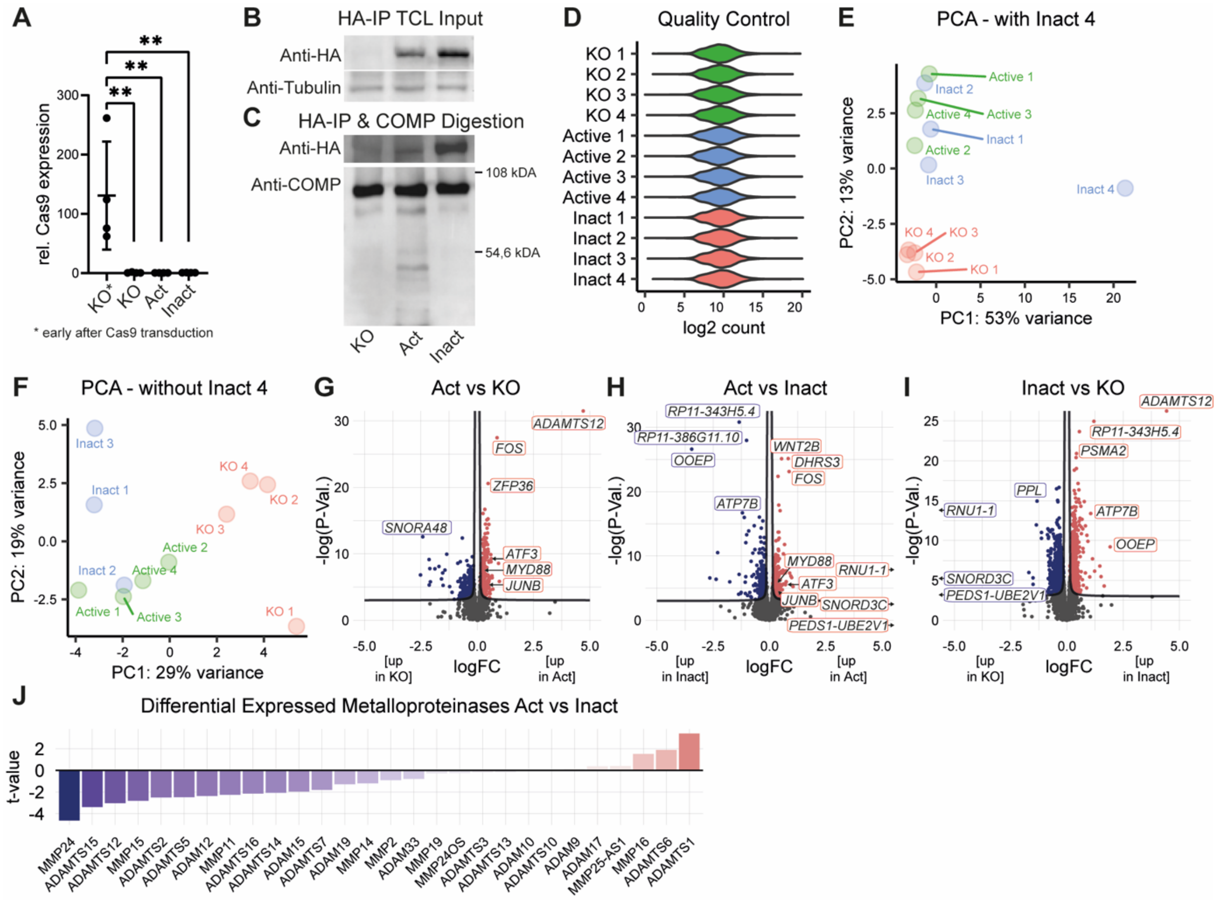

Supplemental Figure 7: Rescue of *ADAMTS12*-KO by overexpression of catalytically active and inactive *ADAMTS12*.

(A) Confirmation of *Cas9* silencing in *ADAMTS12*-KO PDGFRB^{+} cells. *Cas9* gene expression in *ADAMTS12*-KO cells shortly after *ADAMTS12* CRISPR-Cas9-plasmid transduction versus Cas9 expression at later time points (n=4 per group, $p_{\text{KO (before) vs. Active}}<0.0031$). **(B)** Western Blot for HA and Tubulin after HA-ADAMTS12 immunoprecipitation. TCL = Total Cell Lysate. **(C)** Western Blot for HA and COMP after digestion of COMP by active and inactive HA-ADAMTS12. Active, but not inactive ADAMTS12 leads to cleavage of COMP as detected by anti-COMP bands at 54,8 kDA after incubation with active, but not inactive ADAMTS12. **(D)** Gene counts of *ADAMTS12*-KO, active and inactive ADAMTS12 expressing samples after Log2 transformation (n=4 per group). **(E)** Principal Component Analysis of samples shown in D, including the outlier sample “Inactive 4”. **(F)** Principal Component analysis of samples shown in D, excluding the outlier sample “Inactive 4”. **(G)** Volcano Plot showing DEG of inactive ADAMTS12 expressing PDGFRB^{+} cells versus *ADAMTS12*-KO PDGFRB^{+} cells. **(H)** DEG in active vs inactive ADAMTS12 expressing PDGFRB^{+} cells. **(I)** DEG in inactive ADAMTS12 expressing PDGFRB^{+} cells vs *ADAMTS12*-KO PDGFRB^{+} cells. **(J)** Differentially expressed matrix-metalloproteinases in active vs inactive *ADAMTS12* overexpressing PDGFRB^{+} cells ordered by t-value.

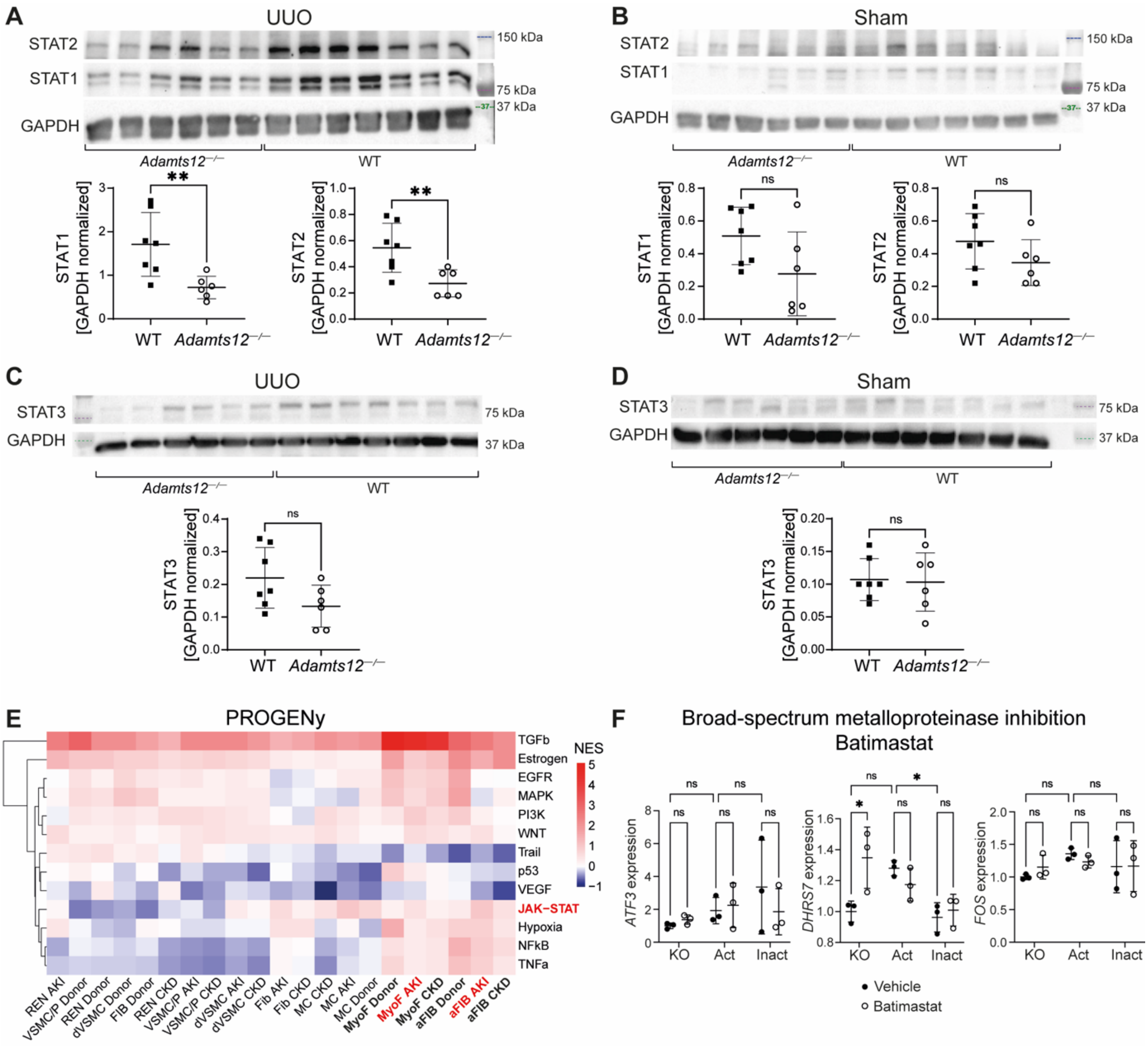

Supplemental Figure 8: Loss of Adamts12 abrogates JAK-STAT signaling in vivo.
(A) Western Blot and band density quantification of STAT1 and STAT2 in kidneys of WT and *ADAMTS12*$^{-/-}$ mice 10 days after UUO surgery (WT n=7, *Adamts12*$^{-/-}$ n=6, p_{STAT1}=0.0095, p_{STAT2}=0.0091) or **(B)** sham surgery (p_{STAT1}=0.08, p_{STAT2}=0.16). **(C-D)** Western Blot and band density quantification of STAT3 after UUO or sham surgery ($p_{STAT3, UUO}$=0.08, $p_{STAT3, Sham}$=0.86). **(E)** PROGENy pathway activity of interstitial cells subsetted from the published KPMP scRNA-seq human kidney dataset stratified by cell type and disease (donor control, AKI, CKD). **(F)** RT-qPCR of injury response fibroblasts marker genes *ATF3*, *DHRS7* and *FOS* after treatment with 20 nM Batimastat or Vehicle in *ADAMTS12* KO, active ADAMTS12 overexpressing and inactive ADAMTS12 overexpressing PDGFRB^{+} cells (n=3 per group). For **(A-D)** an unpaired t-test was performed. For **(F)** a two-way ANOVA with Tukey's post hoc was performed.

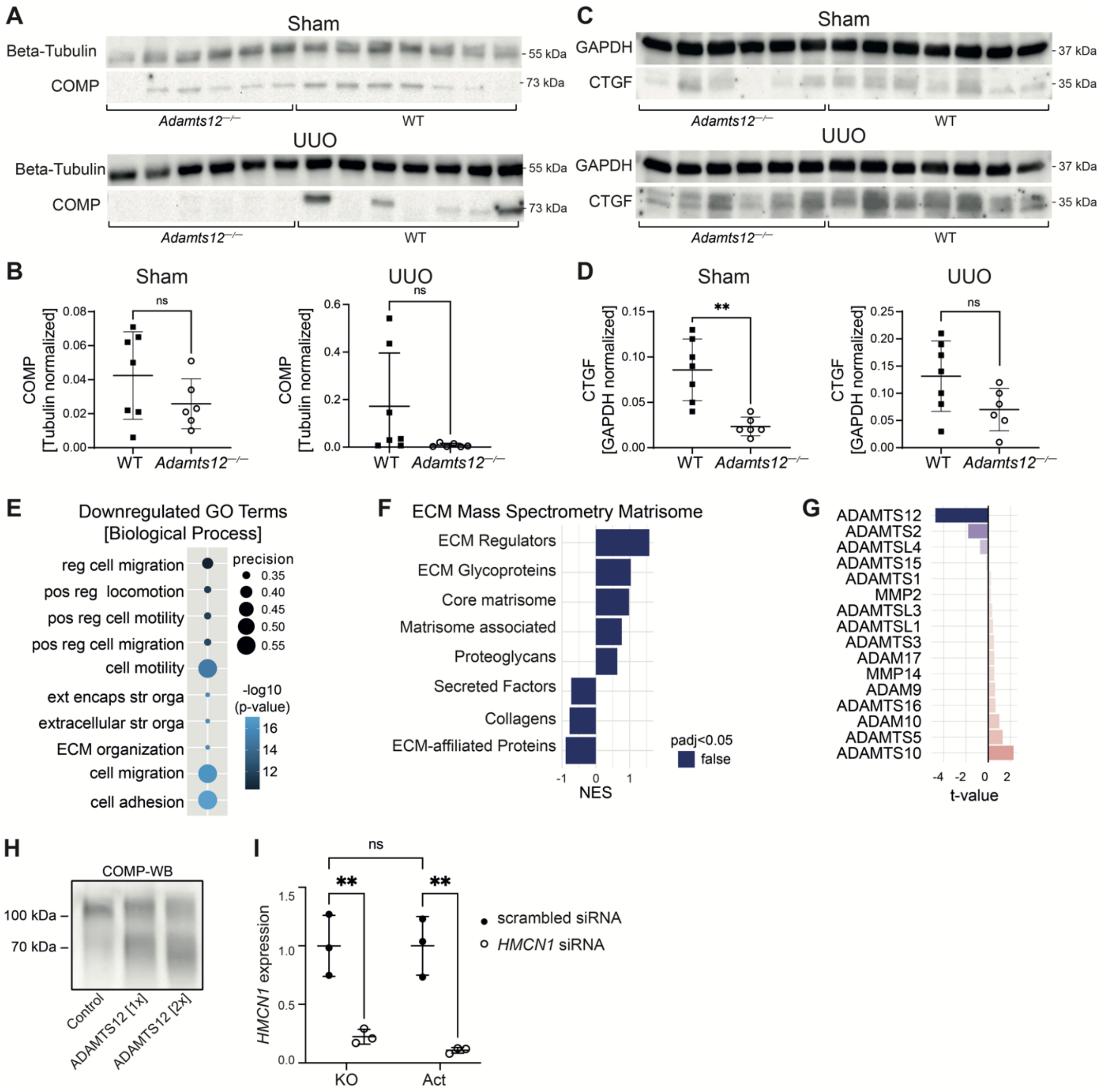

Supplemental Figure 9: HMCN1 is a substrate of ADAMTS12 that facilitates ADAMTS12-induced migration.

(A) Western Blot of COMP in kidneys from WT and *Adamts12*$^{-/-}$ mice 10 days after sham or UUO surgery (WT n=7, *Adamts12*$^{-/-}$ n=6). **(B)** Band density of COMP shown in A normalized by Beta-Tubulin (p_{Sham}=0.19, p_{UUO}=0.10). **(C)** Western Blot of CTGF in above mentioned kidneys. **(D)** Band density of CTGF shown in C normalized by GAPDH (p_{Sham}=0.0013, p_{UUO}=0.068). **(E)** GO-terms based on top downregulated proteins in ECM of WT vs ADAMTS12-KO PDGFRb^{+} cells. **(F)** DB Matrisome gene set enrichment in ECM of WT vs ADAMTS12-KO PDGFRb^{+} cells. **(G)** Top differentially expressed matrix metalloproteinases in the ECM of WT vs KO PDGFRb^{+} cells. **(H)** Western Blot of recombinant COMP after digestion with 90 (1x) or 180 ng (2x) ADAMTS12 (p_{Sham}=0.24, p_{UUO}=0.002815). **(I)** RT-qPCR of HMCN1 after knockdown with HMCN1 or scrambled siRNA in *ADAMTS12*-KO or active *ADAMTS12* expressing cells (n=3 per group, p_{KO}=0.0038, p_{Act}=0.0015, two-way ANOVA with Tukey's post hoc). Unless otherwise specified comparisons were performed with an unpaired t-test.

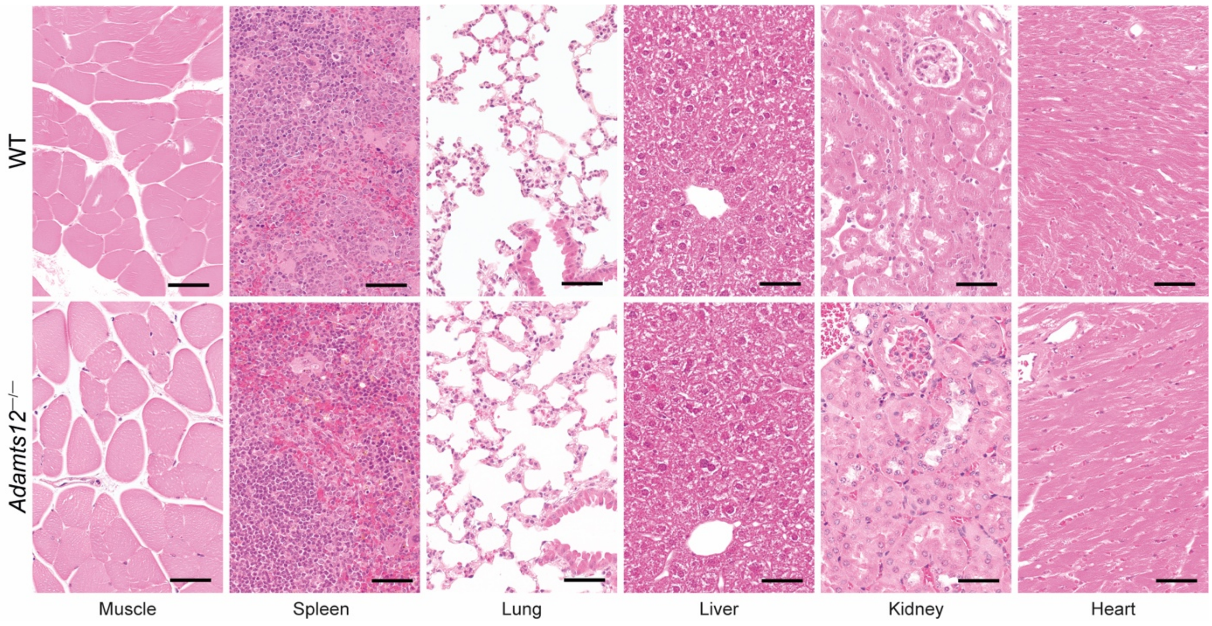

Supplemental Figure 10: Representative HE stainings of hearts, kidneys, livers, lungs, spleens and muscle of WT and *Adamts12*$^{-/-}$ mice. Scale bar: 50 µm. n=6 per group.
