# Supplementary material for: ADAMTS12 promotes fibrosis by restructuring extracellular matrix to enable activation of injury-responsive fibroblasts
Source: J Clin Invest. 2024 Sep 17;134(18):e170246. doi: 10.1172/JCI170246 (PMC11405035; doi:10.1172/JCI170246)
Supplement: Unedited blot and gel images [file jci-134-170246-s067.pdf]

Full unedited Western Blots for Figure 6B

Anti-HA Western Blot

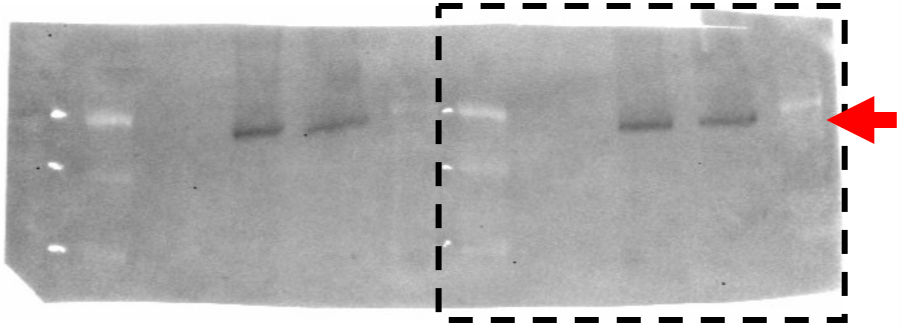

Anti-Tubulin Western Blot

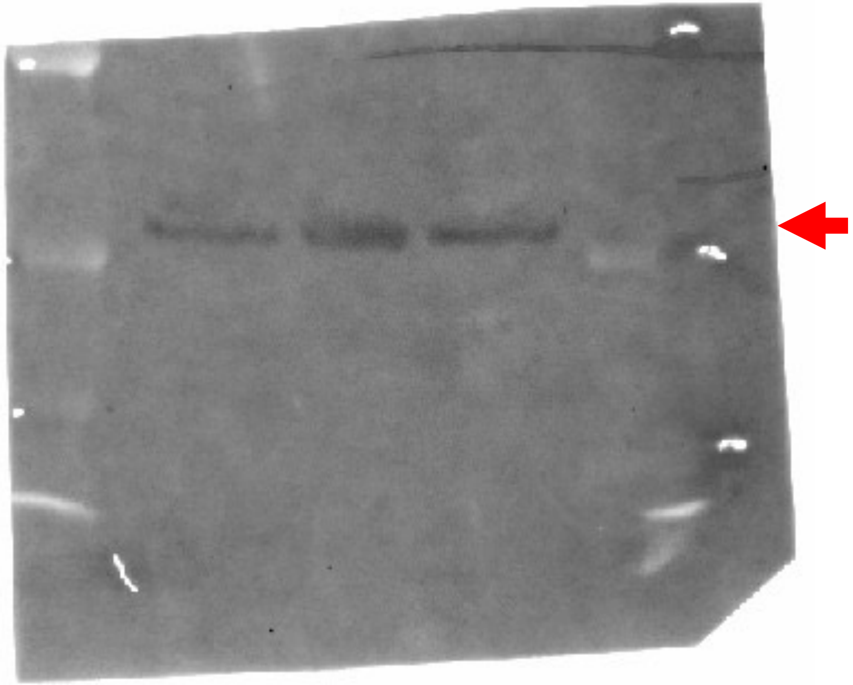

Anti-GFP Western Blot

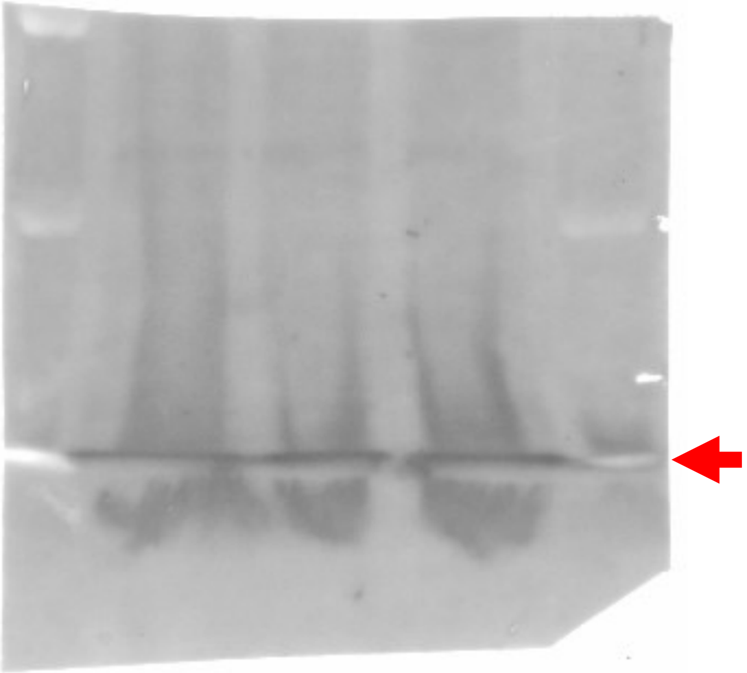

Full unedited Western Blots for Figure 7B

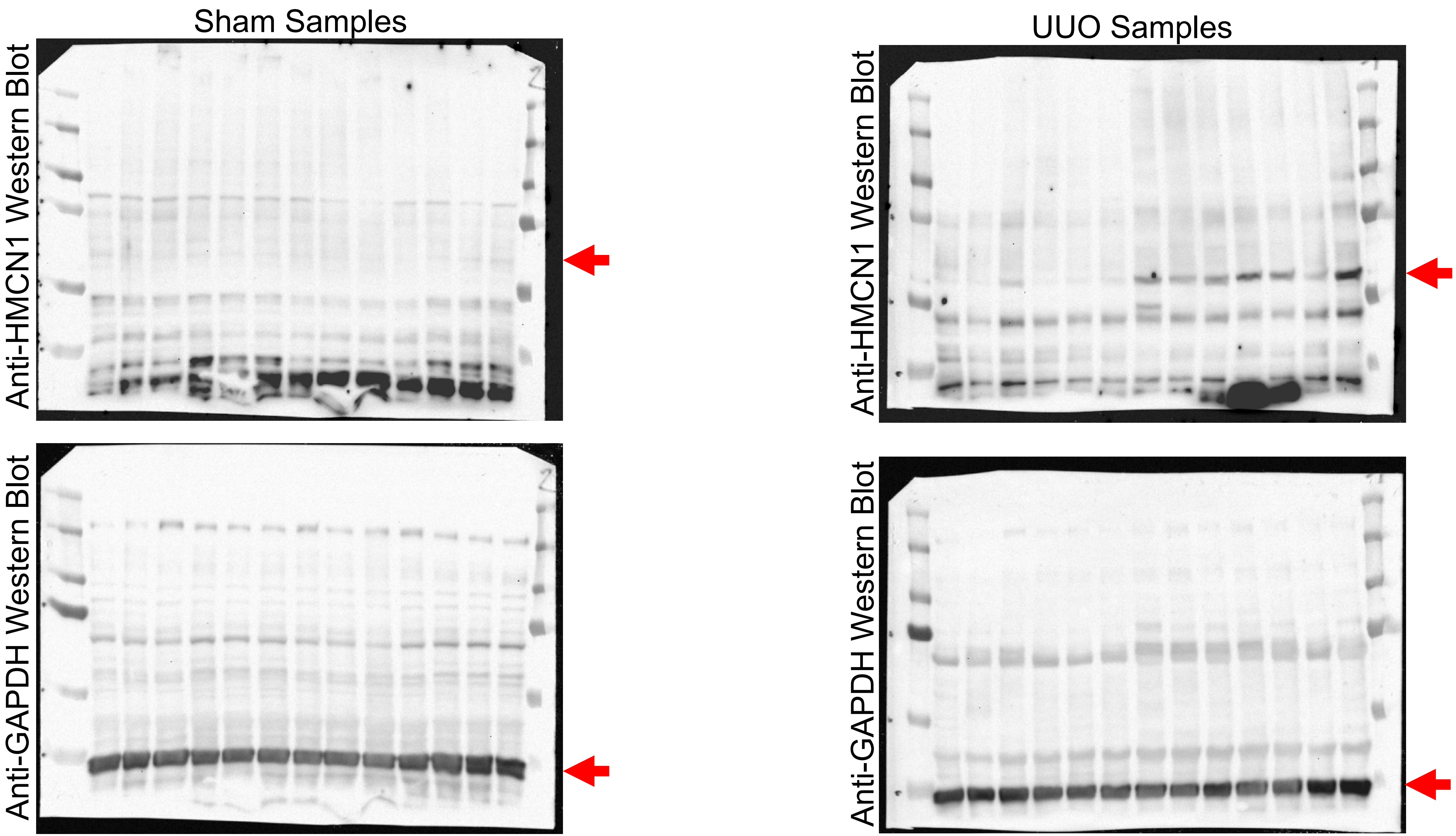

Full unedited Western Blots for Figure 7D

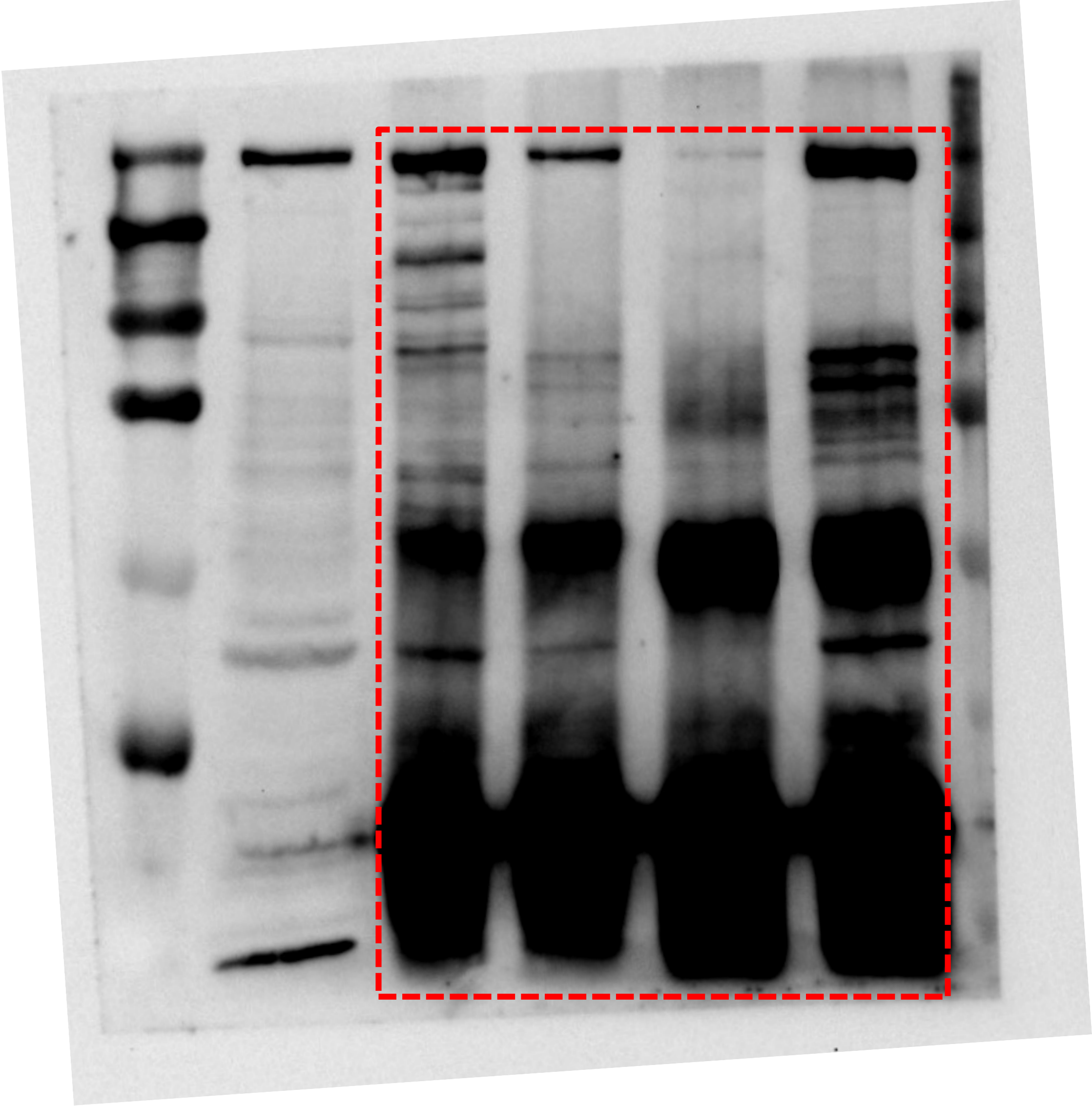

Full unedited Western Blots for Figure 7E

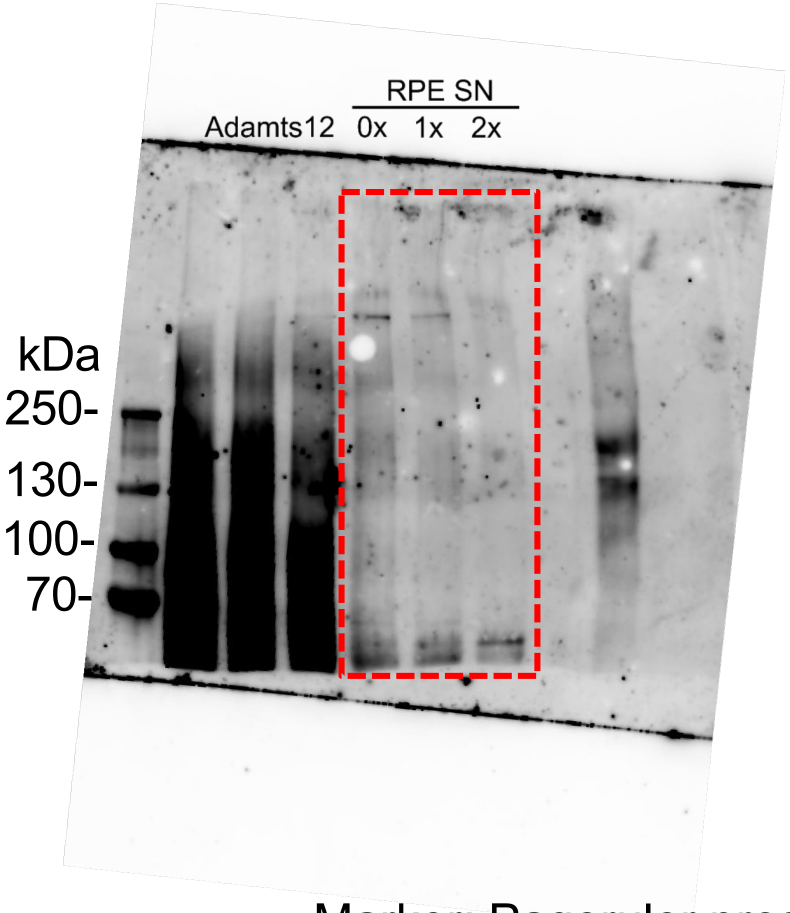

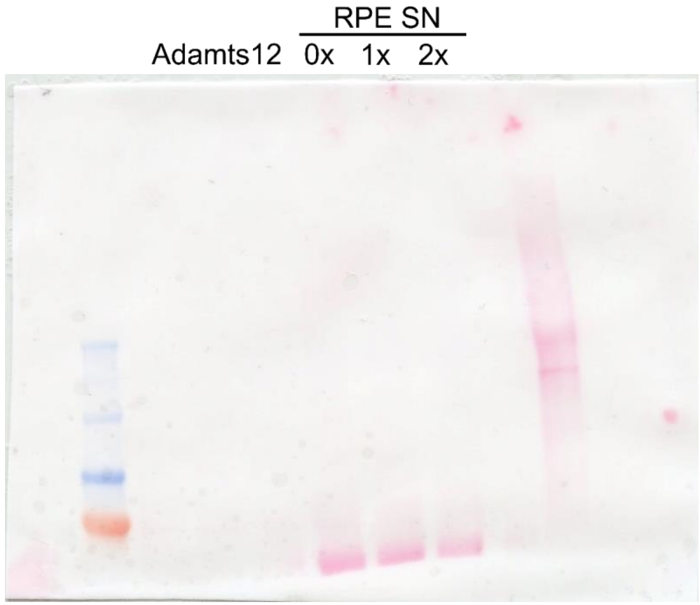

Marker: Pageruler prestained plus + unreduced TG

Full unedited Western Blots for Supplemental Figure 3B

Anti-aSMA Western Blot

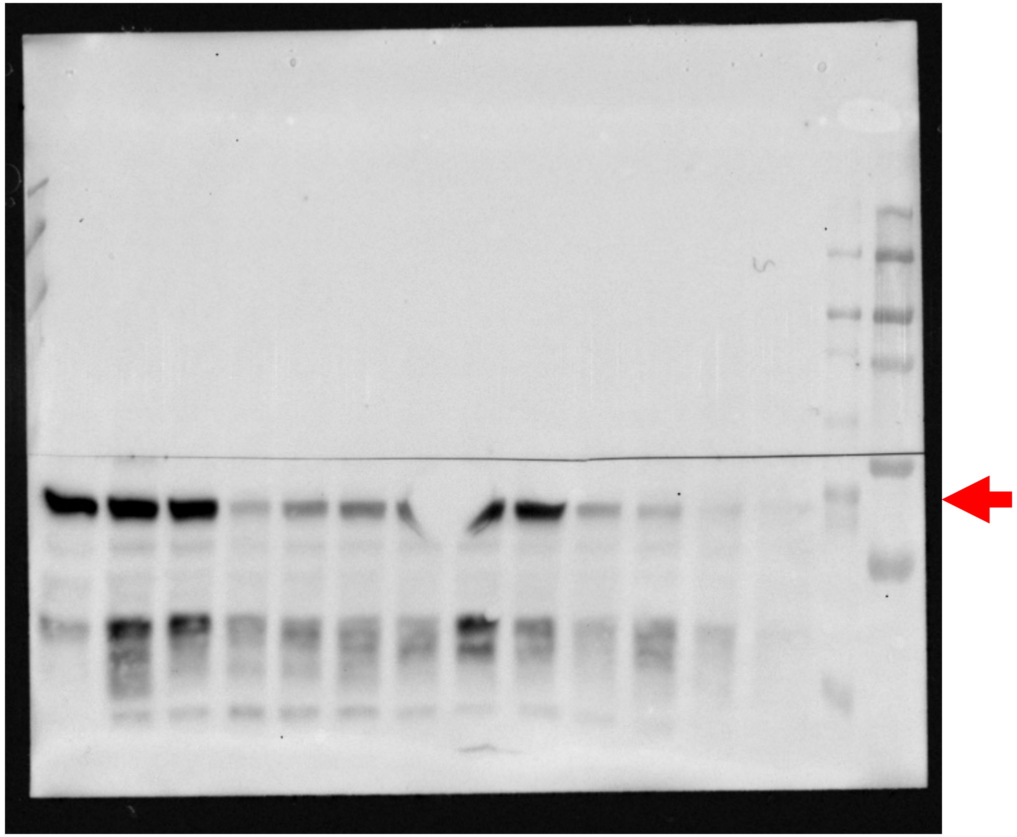

Anti-GAPDH Western Blot

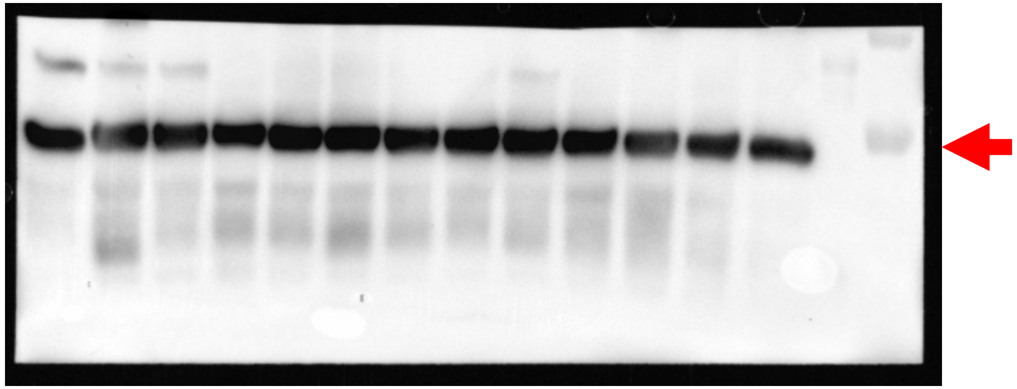

Full unedited Western Blots for Supplemental Figure 3C

Anti-aSMA Western Blot

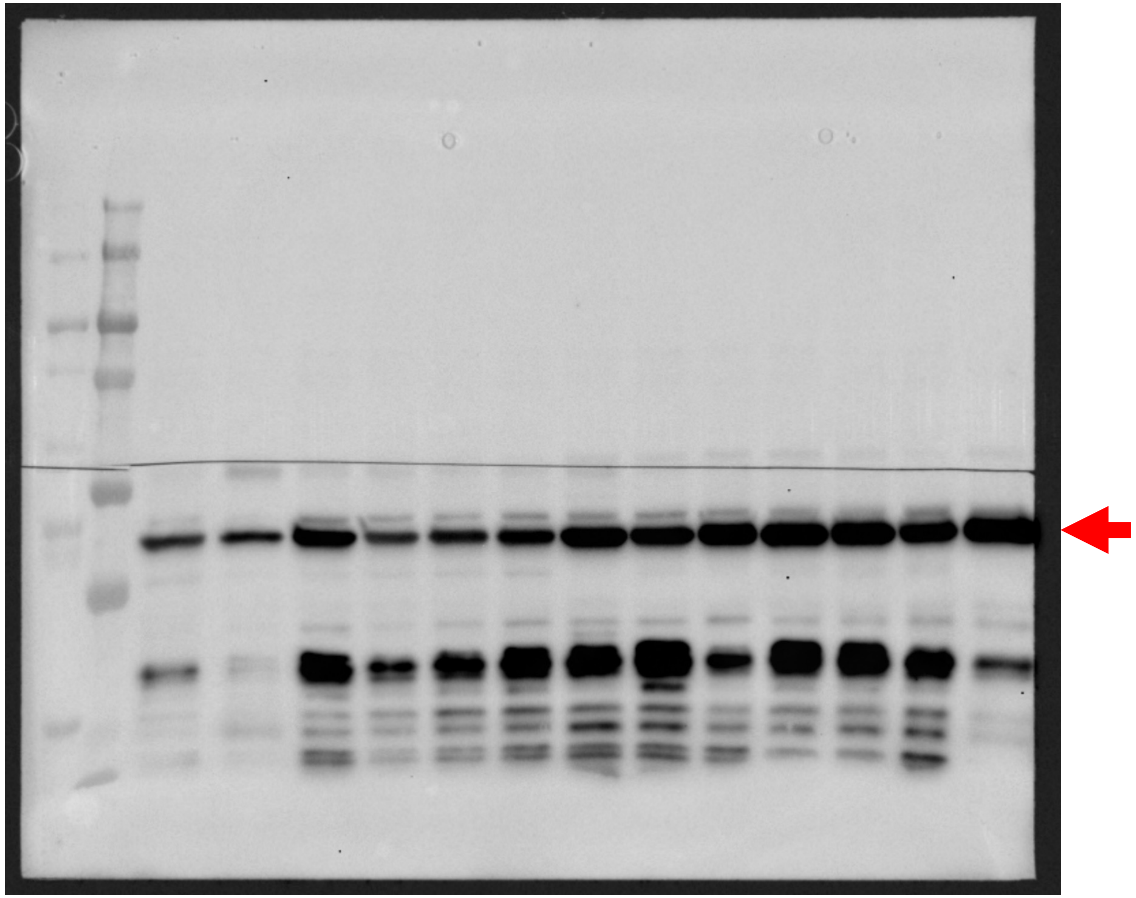

Anti-GAPDH Western Blot

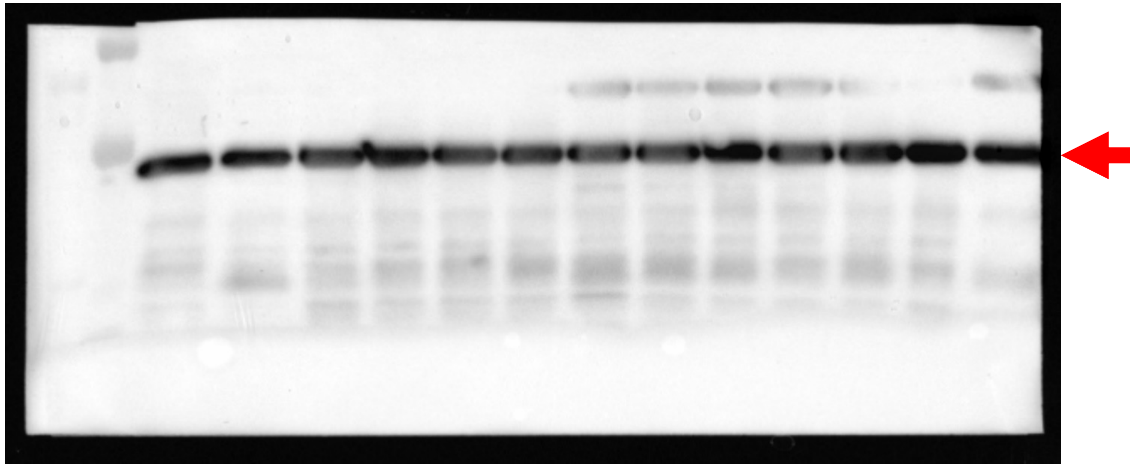

Full unedited Western Blots for Supplemental Figure 7B

Anti-HA Western Blot

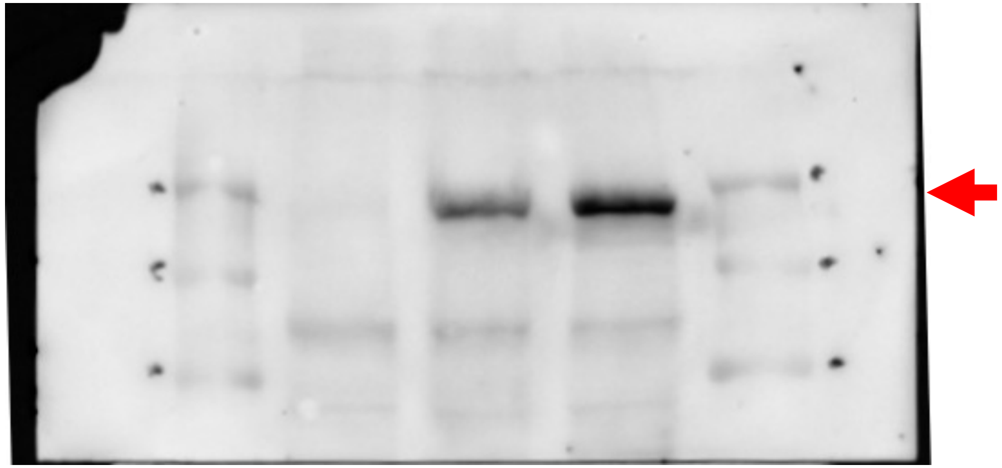

Anti-Tubulin Western Blot

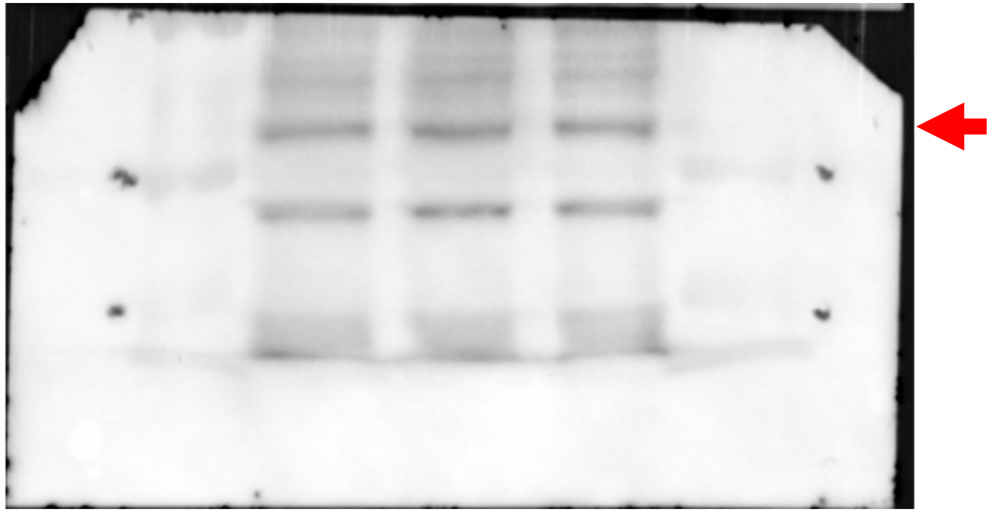

Full unedited Western Blots for Supplemental Figure 7C

Anti-HA Western Blot

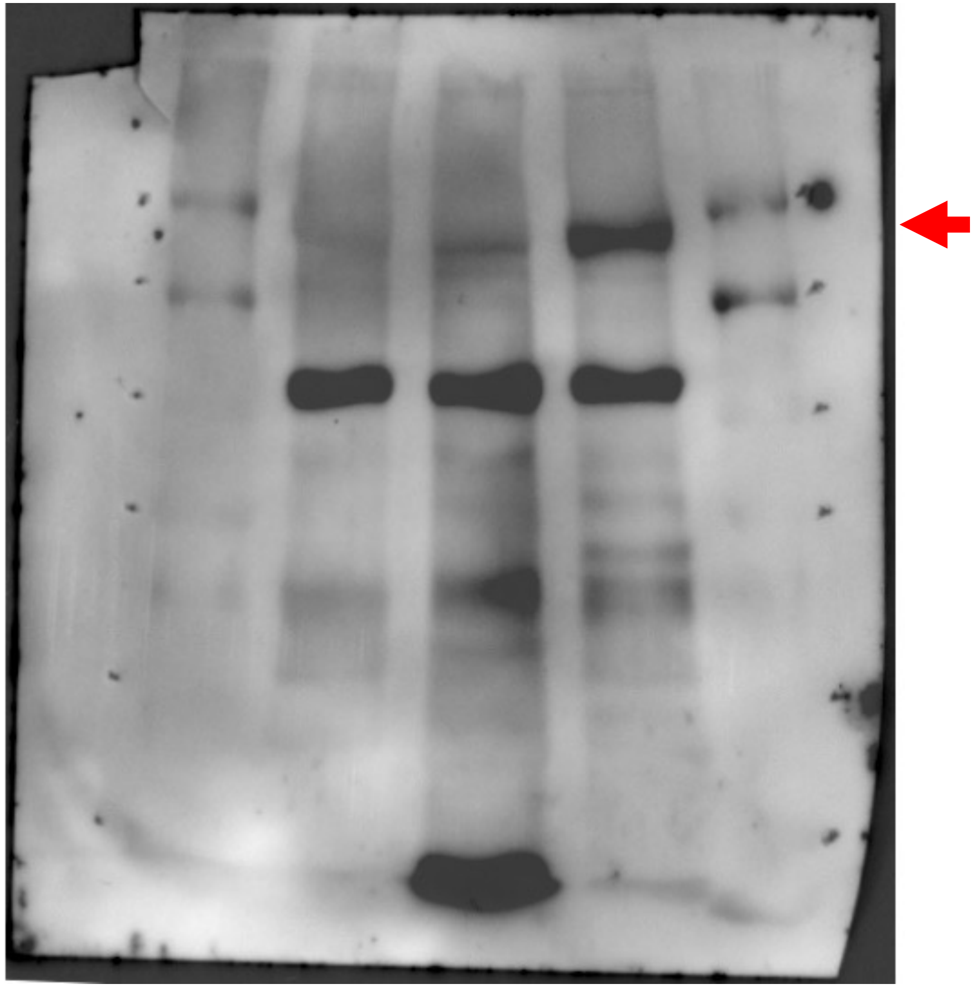

Anti-COMP Western Blot

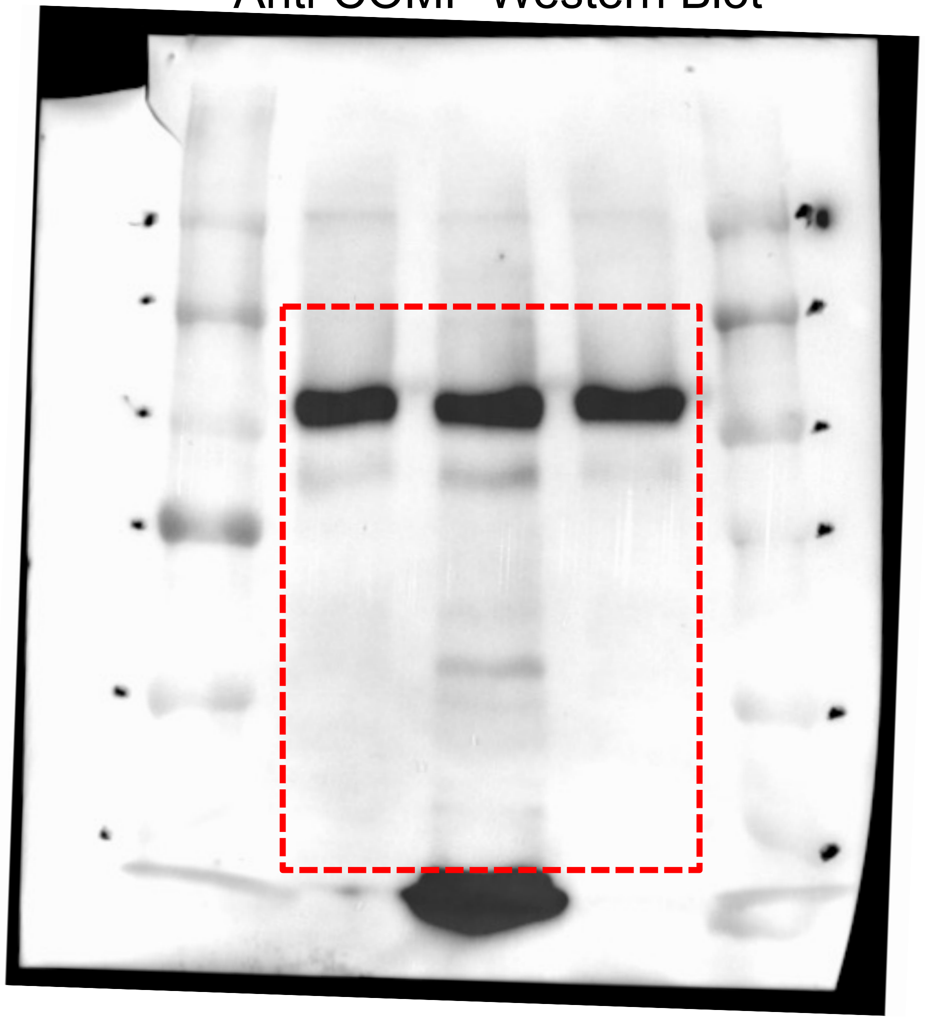

Full unedited Western Blots for Supplemental Figure 8A

Anti-STAT1 Western Blot

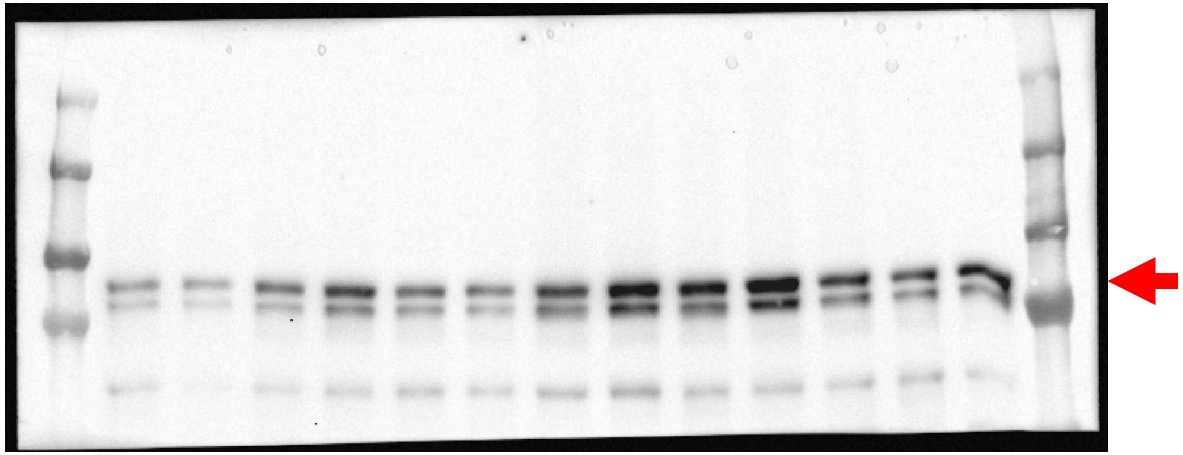

Anti-STAT2 Western Blot

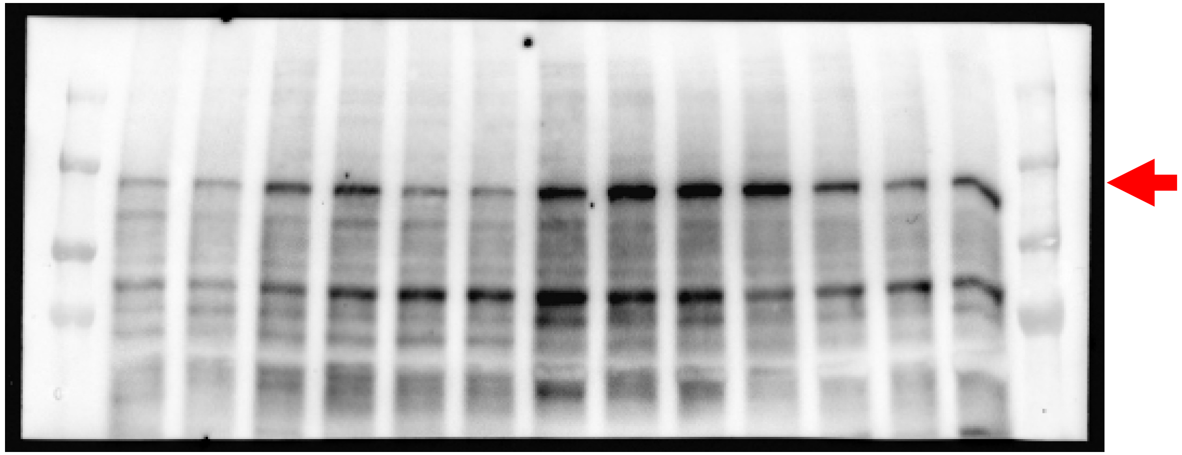

Anti-GAPDH Western Blot

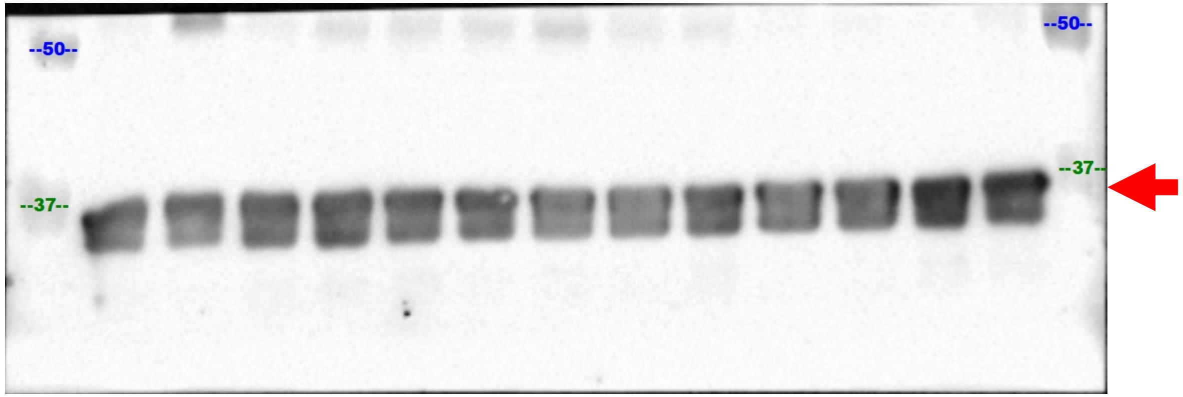

Full unedited Western Blots for Supplemental Figure 8B

Anti-STAT1 Western Blot

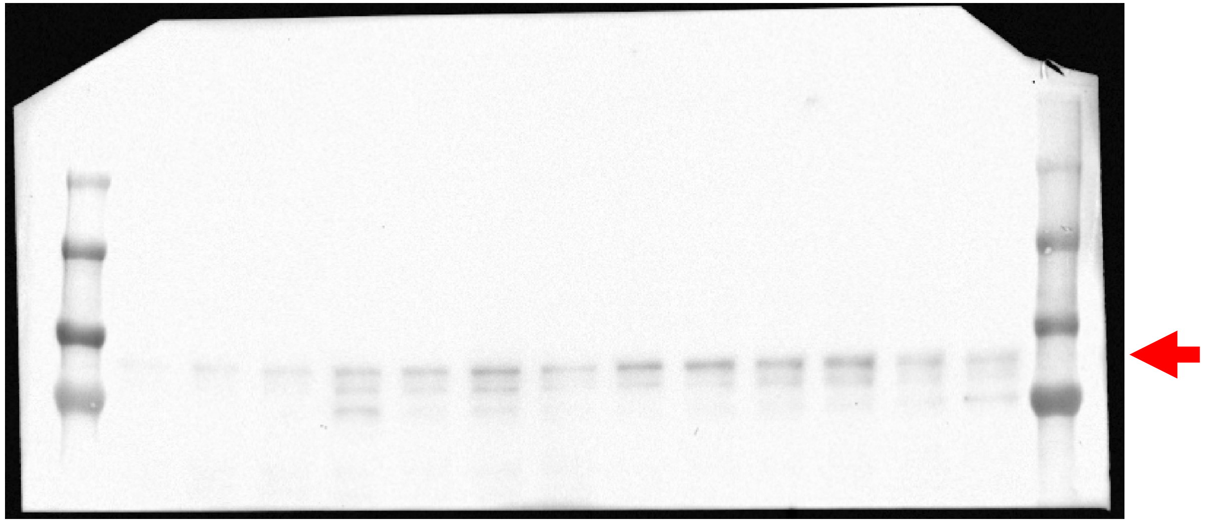

Anti-STAT2 Western Blot

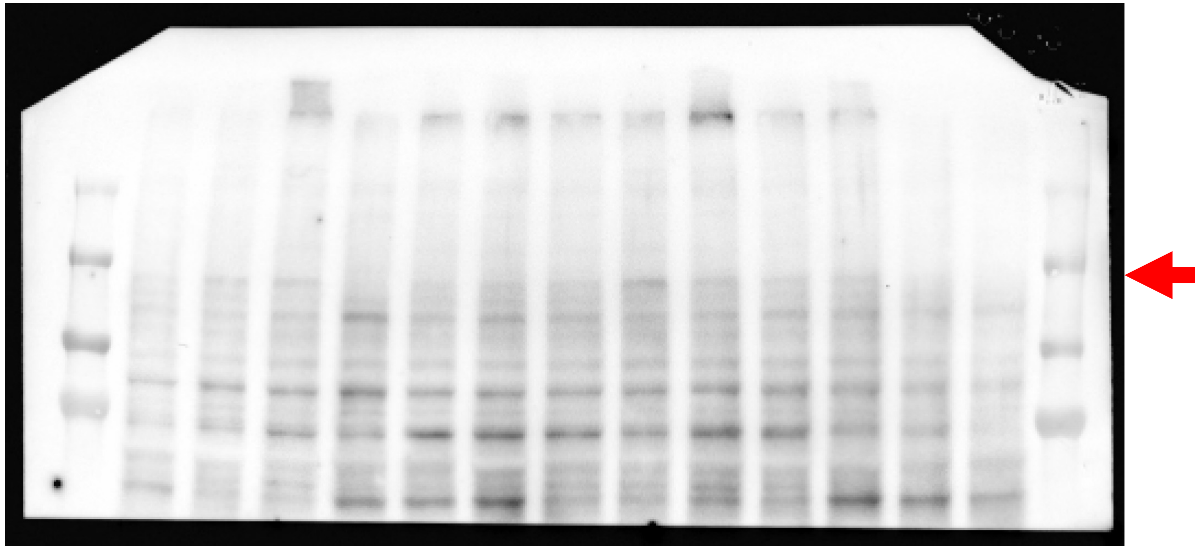

Anti-GAPDH Western Blot

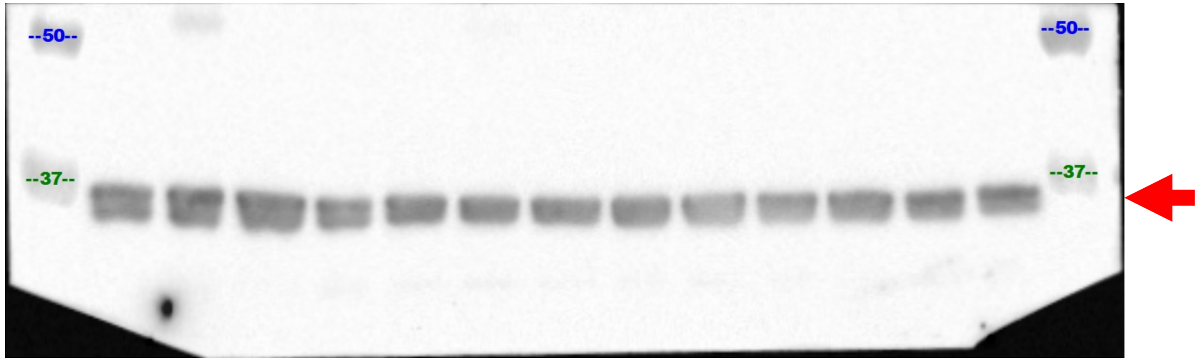

Full unedited Western Blots for Supplemental Figure 8C

Anti-STAT3 Western Blot

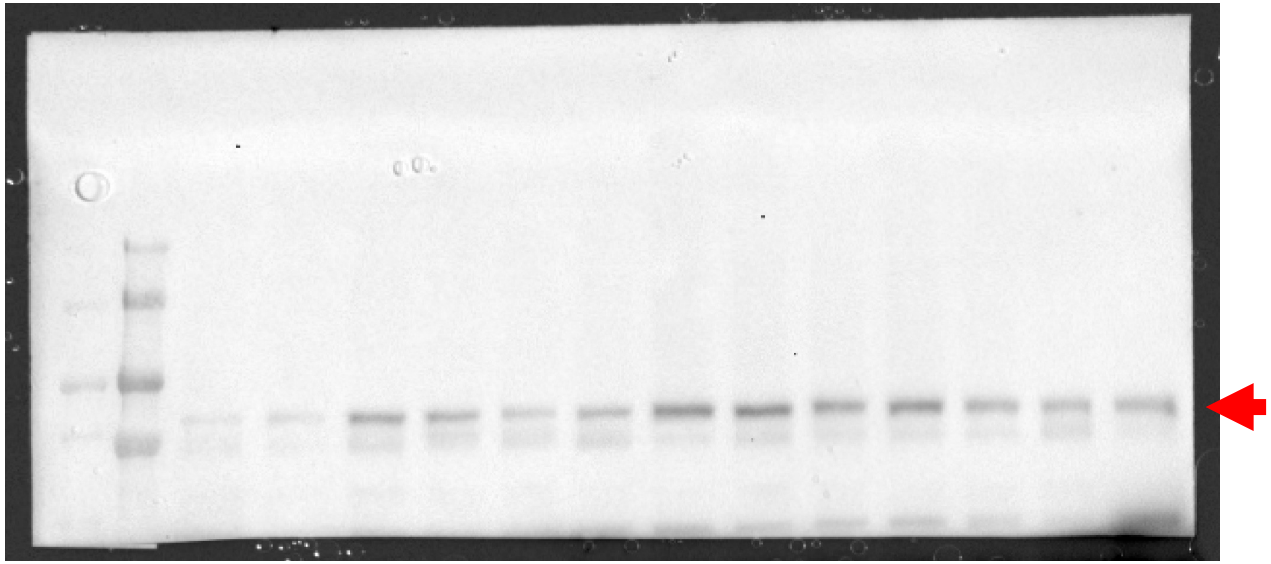

Anti-GAPDH Western Blot

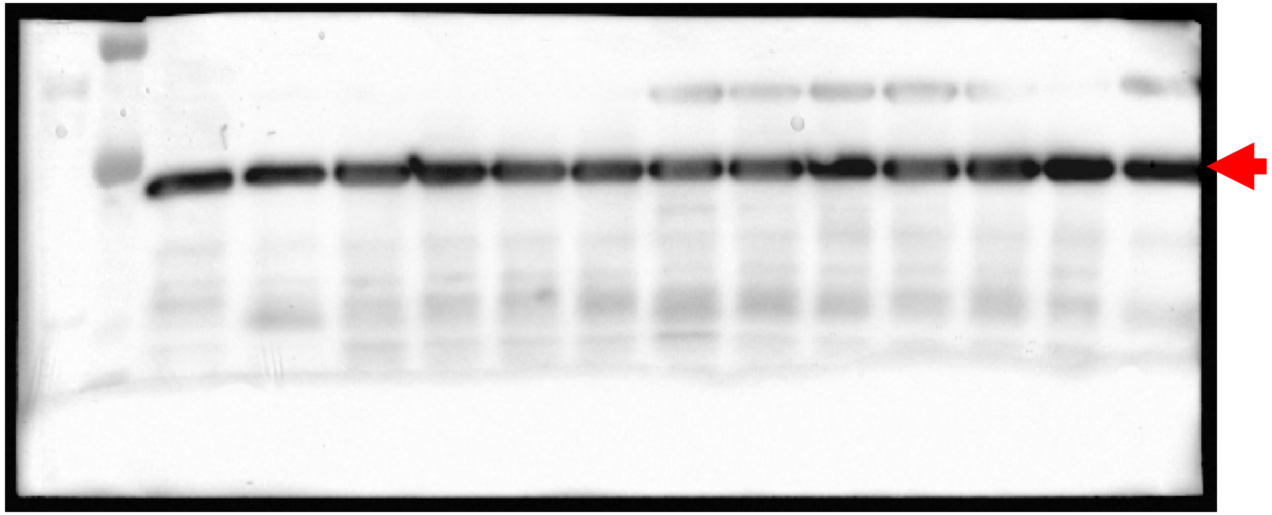

Full unedited Western Blots for Supplemental Figure 8D

Anti-STAT3 Western Blot

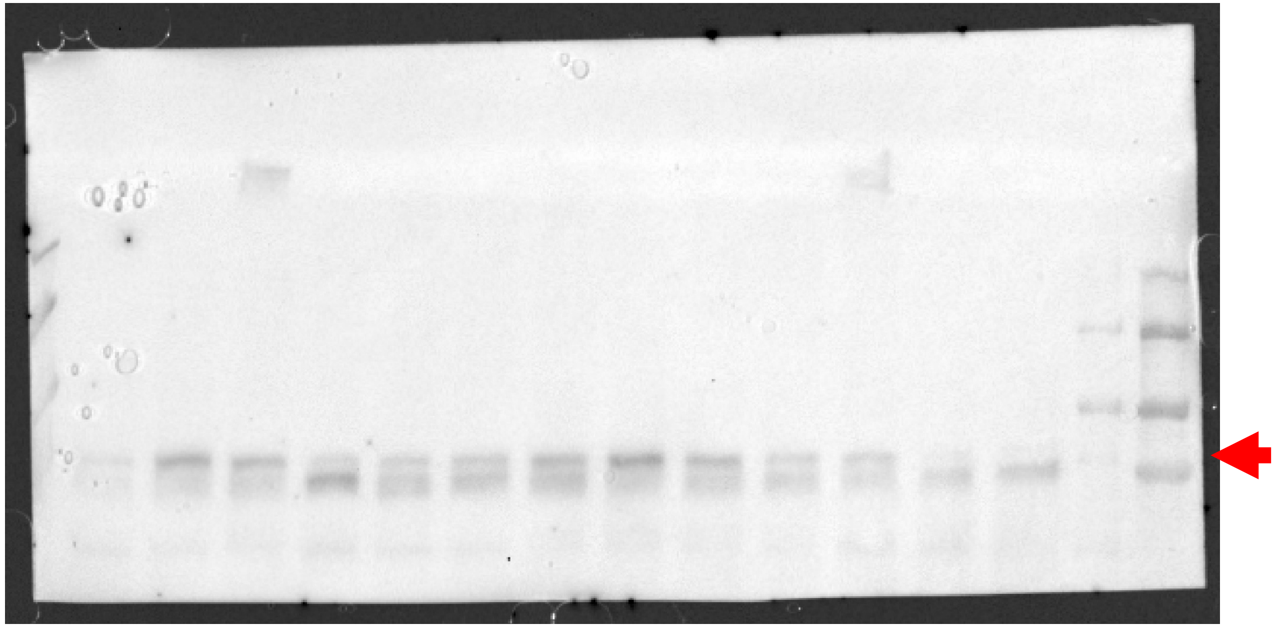

Anti-GAPDH Western Blot

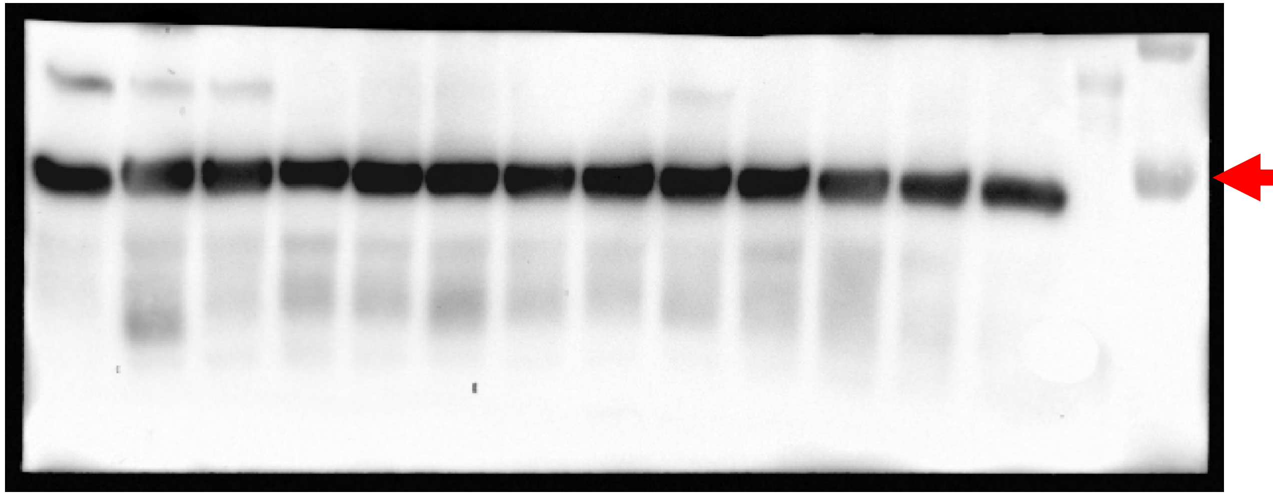

Full unedited Western Blots for Supplemental Figure 9A

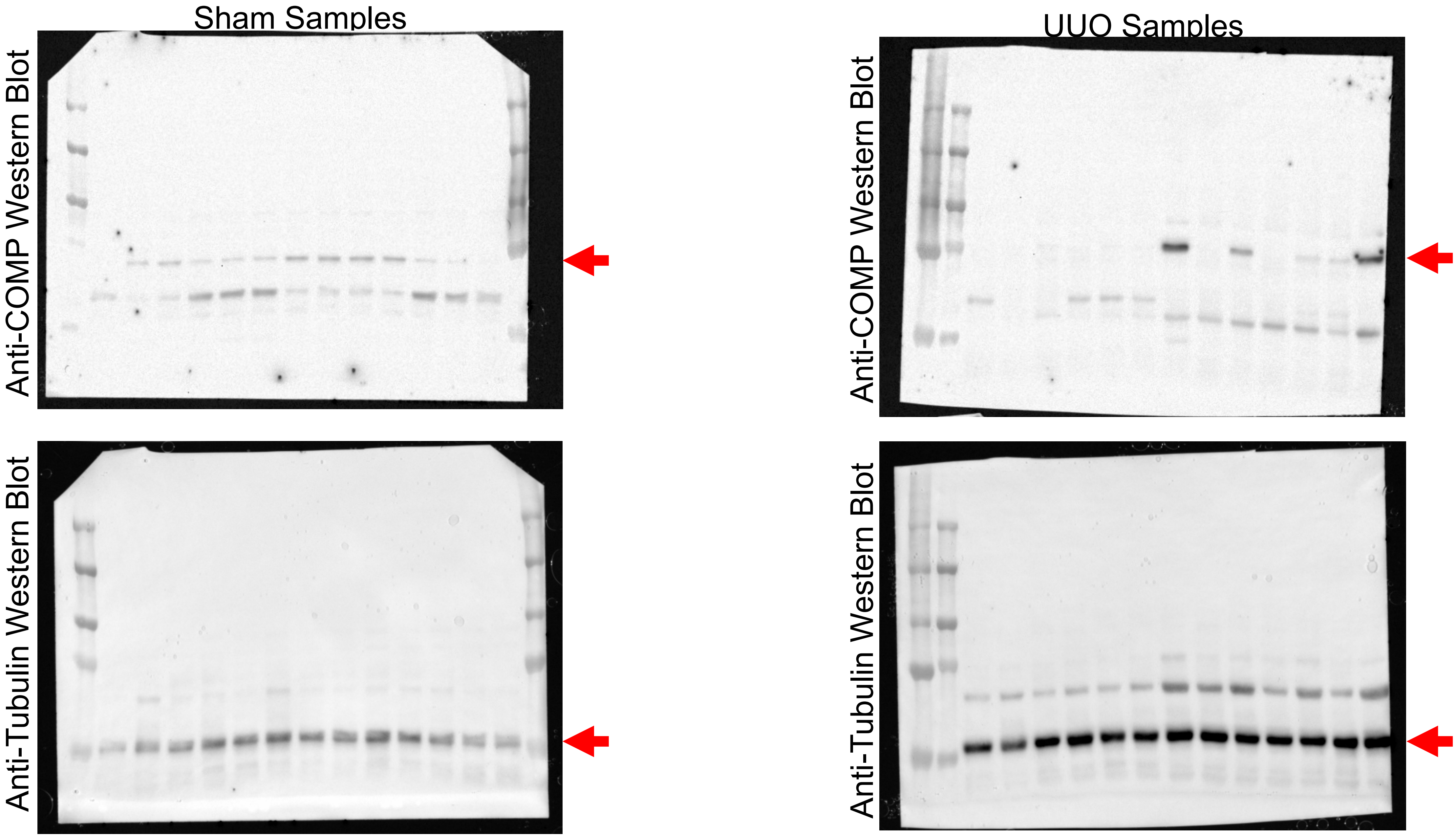

Full unedited Western Blots for Supplemental Figure 9C

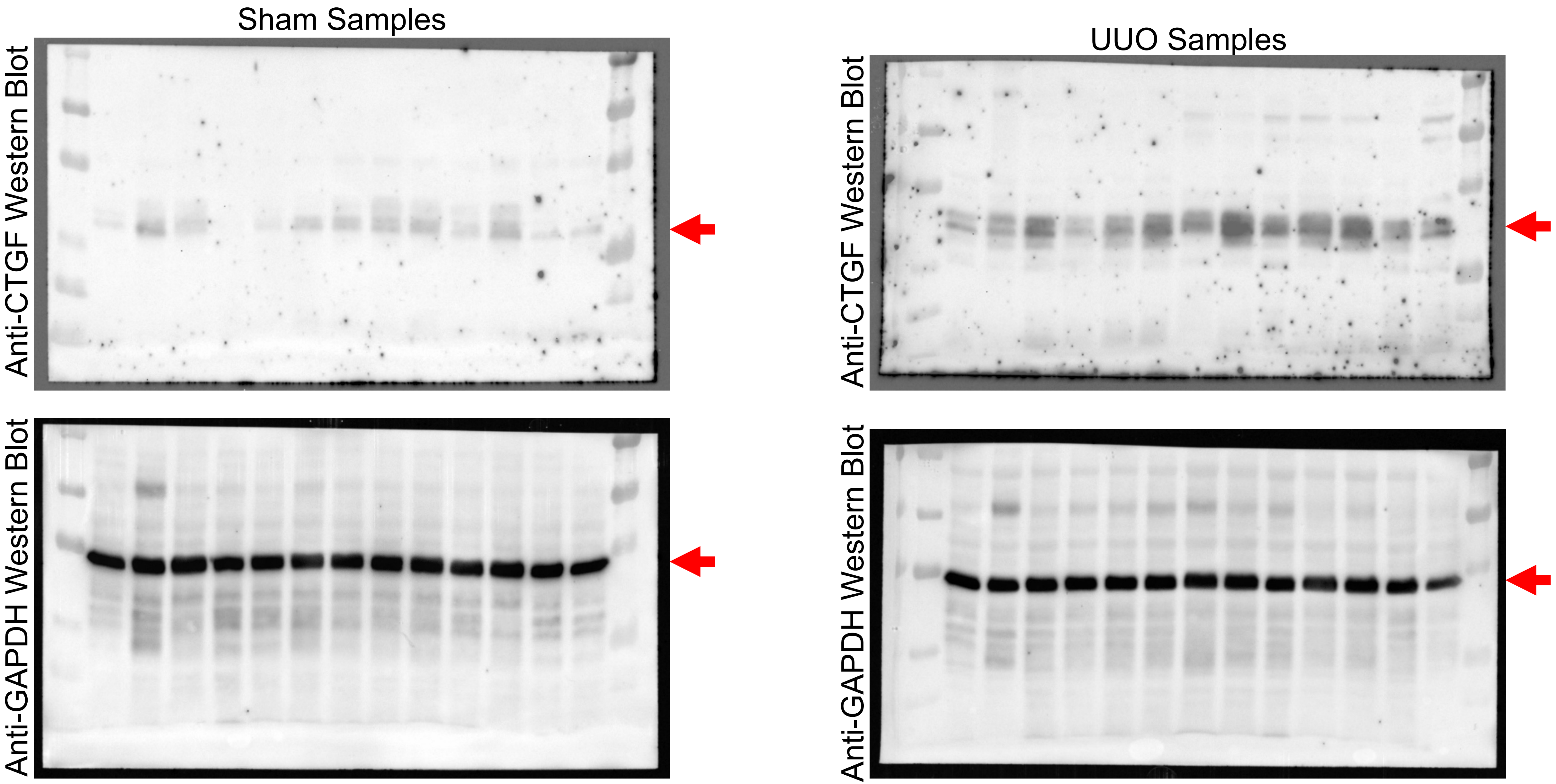

Full unedited Western Blots for Supplemental Figure 9H

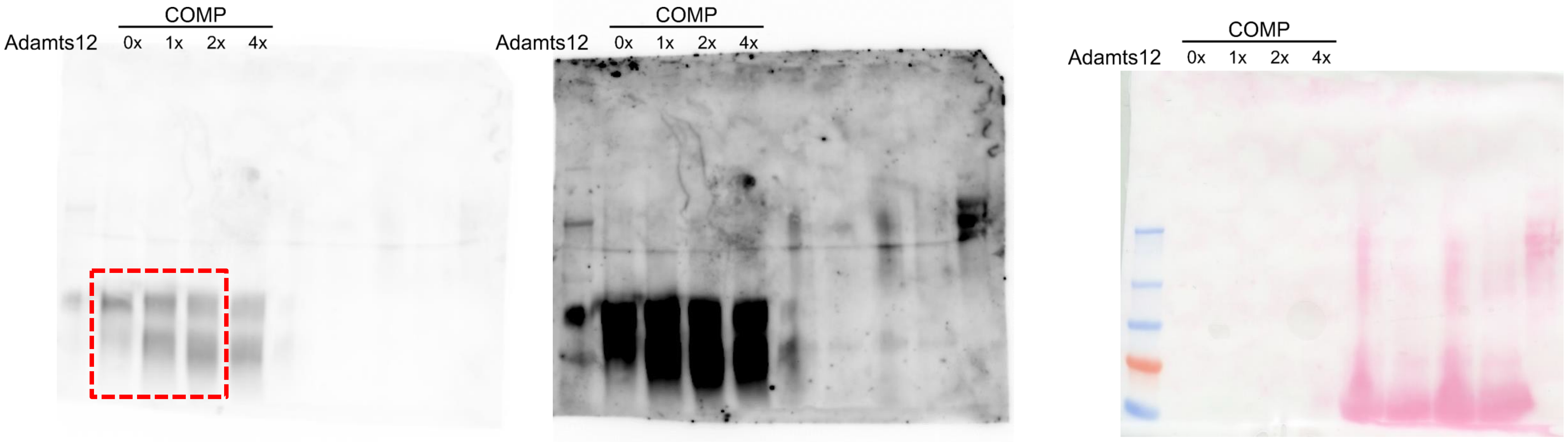

Marker: Pageruler prestained plus + unreduced TG
